# Supplementary figures and images for: Drug repurposing for aging research using model organisms
Source: Aging Cell. 2017 Jun 16;16(5):1006–15. doi: 10.1111/acel.12626 (PMC5595691; doi:10.1111/acel.12626)

**A***Drosophila*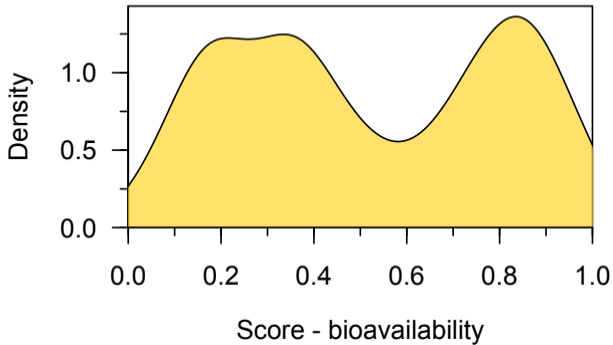**B***C. elegans*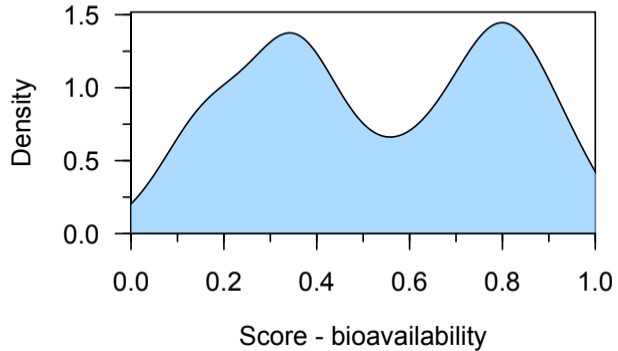

Supplement: Supplementary file 1 — Fig. S1 Density distribution of score without bioavailability term. [file ACEL-16-1006-s001.pdf]
