# Supplementary material for: Drug repurposing for aging research using model organisms
Source: Aging Cell. 2017 Jun 16;16(5):1006–15. doi: 10.1111/acel.12626 (PMC5595691; doi:10.1111/acel.12626)
Supplement: Supplementary file 2 — Fig. S2 Comparison of 47X and genistein (GEN) in binding pocket of MIF. [file ACEL-16-1006-s002.pdf]

Supplement: Drug repurposing for ageing research using model organisms

Supporting Information S7

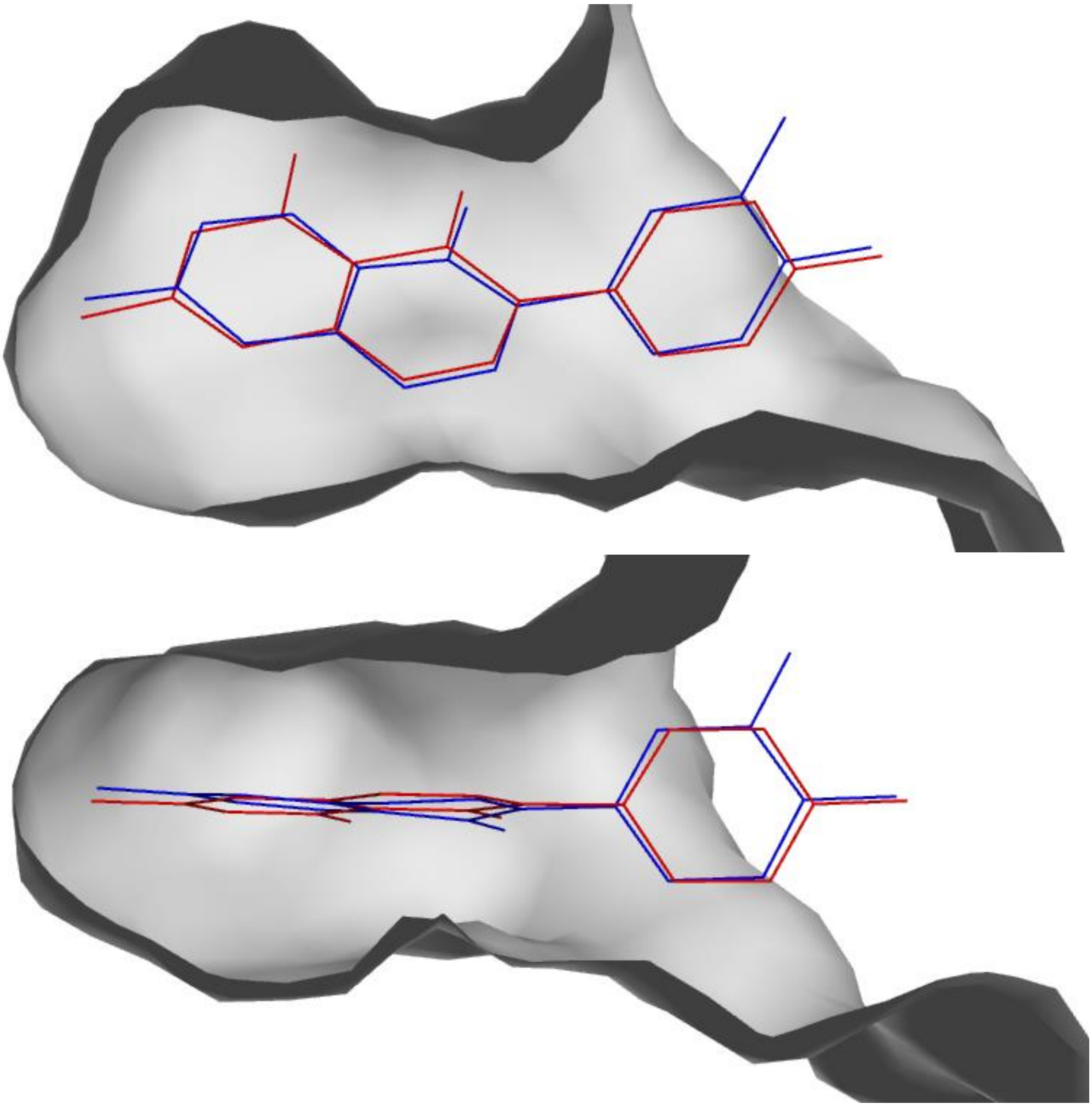

Binding pocket of 47X (blue) on Macrophage migration inhibitory factor (MIF; PDB 3L5R) overlaid with docked Genistein (PDB Hetcode GEN, red). Clearly visible is the high chemical similarity between GEN and 47X. GEN has slightly higher predicted bioavailability in C. elegans compared to 47X (GEN: Burns score = 8.75, Bioavailability score = 0.91; 47X: Burns score = 6.47, Bioavailability score = 0.8). However, this is contrasted by the more than 100-fold lower predicted binding affinity (GEN: RF-Score = 4.13, Binding score = 0.3; 47X: RF-Score = 6.67; Binding score = 0.842).
