# Supplementary material for: Drug repurposing for aging research using model organisms
Source: Aging Cell. 2017 Jun 16;16(5):1006–15. doi: 10.1111/acel.12626 (PMC5595691; doi:10.1111/acel.12626)
Supplement: Supplementary file 4 — Table S2 Table of Top 15 scoring compounds for D. melanogaster and C. elegans targeting at least one protein implied in aging not targeted by a higher ranked compound. [file ACEL-16-1006-s004.pdf]

Supplement: Drug repurposing for ageing research using model organisms

Top 15 scoring compounds for *D. melanogaster* targeting at least one protein implied in ageing not targeted by a higher ranked compound.

Rank	PDB HET Code	Name*	Targets**	global identity	binding site identity	Ageing Implication	Domain conservation	Binding site conservation	Binding affinity	Bio-availability	Lipinski Loss	Promiscuity Loss	Purchasability Bonus	Drug approval Bonus	Final Score
1	1N1	Dasatinib(Sprycel)	BMX, BTK, MK14	67%	100%	1.	0.96	1.	0.95	0.9	0.	0.	0.1	0.1	1.00
2	CT5	NA	HSP90α	77%	92%	0.98	0.97	1.	0.95	0.9	0.	0.	0.1	0.08	0.99
5	TAK	Dorsomorphin	AMPKα2	56%	100%	1.	0.97	1.	0.9	0.9	0.	0.	0.1	0.08	0.96
6	STI	Imatinib(Gleevec)	ABL1, MK14	67%	94%	1.	0.97	0.99	0.88	0.9	0.	0.	0.1	0.1	0.95
7	P01	Purvalanol	S6Kα1	46%	100%	1.	0.96	1.	0.89	0.9	0.	0.	0.1	0.08	0.95
9	JNF	NA	MK10	62%	100%	1.	0.97	1.	0.87	0.9	0.	0.	0.1	0.08	0.94
14	GVP	NA	AKT2	49%	78%	1.	0.96	0.91	0.93	0.9	0.	0.	0.1	0.08	0.91
15	NIL	Nilotinib(Tasigna)	ABL1, MK11	61%	100%	1.	0.97	1.	0.93	0.9	-0.1	0.	0.1	0.1	0.91
17	X6K	PI-103	mTOR	52%	100%	1.	0.96	1.	0.93	0.9	0.	0.	0.1	0.	0.91
18	B96	Doramapimod	MAPK9, PTK2β, MAPK14	67%	94%	1.	0.97	0.99	0.96	0.9	-0.1	0.	0.1	0.08	0.91
20	RRC	Seliciclib	CDK5	78%	100%	1.	0.97	1.	0.91	0.9	0.	0.	0.1	0.	0.89
32	537	NA	MAPK8	66%	100%	1.	0.97	1.	0.79	0.9	0.	0.	0.1	0.08	0.86
40	EBI	BMS-754807	IGF1R	23%	94%	1.	0.93	0.95	0.93	0.9	0.	0.	0.1	0.	0.84
51	ZS4	NA	PI3Kδ	37%	94%	1.	0.91	0.94	0.95	0.9	0.	0.	0.1	0.	0.83
70	21B	Nortriptyline	DAT	100%	100%	0.79	0.98	1.	0.87	0.9	0.	0.	0.1	0.1	0.81

Top 15 scoring compounds for *C. elegans* targeting at least one protein implied in ageing not targeted by a higher ranked compound.

Rank	PDB HET Code	Name*	Targets**	global identity	binding site identity	Score	Ageing Implication	Domain conservation	Binding site conservation	Binding affinity	Bio-availability	Lipinski Loss	Promiscuity Loss	Purchasability Bonus	Drug approval Bonus
1	STI	Imatinib(Gleevec)	ABL1, MAPK14	62%	91%	1.	1.	0.96	0.99	0.88	0.97	0.	0.	0.1	0.1
2	NIL	Nilotinib(Tasigna)	ABL1, MAPK11	59%	83%	0.91	1.	0.95	0.96	0.93	0.95	-0.1	0.	0.1	0.1
3	GVP	NA	AKT2	49%	78%	0.86	1.	0.95	0.95	0.93	0.81	0.	0.	0.1	0.08
5	X6K	PI-103	mTOR	31%	100%	0.71	1.	0.94	1.	0.93	0.69	0.	0.	0.1	0.
8	JBI	NA	MK10	45%	93%	0.68	1.	0.97	1.	0.91	0.83	-.05	0.	0.	0.
9	TAK	Dorsomorphin	AMPKα2	51%	91%	0.66	1.	0.97	0.92	0.9	0.6	0.	0.	0.1	0.08
14	D94	NA	IGF1R	20%	75%	0.57	1.	0.87	0.83	0.9	0.95	-.05	0.	0.	0.
15	YI0	NA	HSP90α	74%	86%	0.56	0.98	0.97	0.92	0.88	0.74	0.	0.	0.	0.
17	IXM	Indirubin-3'-Monoxime	GSK3β	74%	91%	0.55	1.	0.97	0.97	0.8	0.51	0.	0.	0.1	0.08
21	47X	NA	MIF	33%	75%	0.53	0.81	0.85	0.93	0.84	0.8	0.	0.	0.1	0.
23	LW4	Sotrastaurin	PKCα	66%	81%	0.53	0.62	0.96	0.91	0.95	0.83	0.	0.	0.1	0.
24	351	NA	IR	20%	82%	0.53	1.	0.86	0.86	0.96	0.88	-0.1	0.	0.	0.
27	VX6	Tozasertib	AURKB	49%	100%	0.52	0.79	0.94	1.	0.93	0.6	0.	0.	0.1	0.
28	ZS4	NA	PI3Kδ	23%	82%	0.52	1.	0.76	0.89	0.95	0.65	0.	0.	0.1	0.
41	12C	NA	CHK1	30%	88%	0.45	0.56	0.88	0.87	0.94	0.67	0.	0.	0.1	0.08
