# Supplementary material for: Drug repurposing for aging research using model organisms
Source: Aging Cell. 2017 Jun 16;16(5):1006–15. doi: 10.1111/acel.12626 (PMC5595691; doi:10.1111/acel.12626)
Supplement: Supplementary file 7 — Data S1 Zip‐Archive of all report cards. [file ACEL-16-1006-s007.zip › RC_022.pdf]

# 022

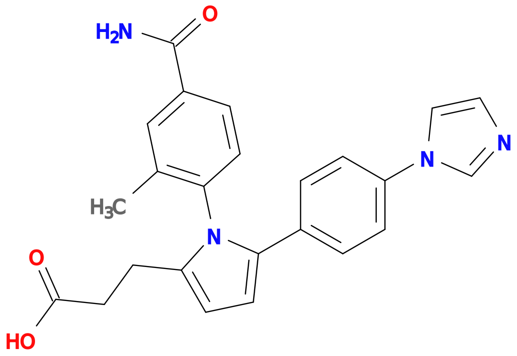


**Database identifiers**
ChEMBLCompound CHEMBL1738699

## Ranking

| | Rank | Score |
|---|---|---|
| Drosophila | 278/697 | 0.597 |
| C. elegans | 388/591 | 0.147 |

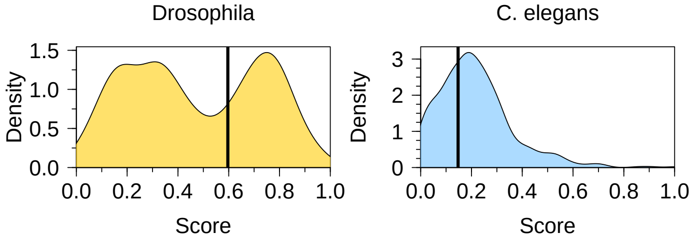


| | Ageing implication | Domain conservation | Binding site conservation | Binding affinity | Bioavailability | Lipinski | Promiscuity | Purchasability | Drug approval | **Total** |
|---|---|---|---|---|---|---|---|---|---|---|
| Drosophila | 0.792 | 0.959 | 0.947 | 0.923 | (0.9) | 0.0 | -0.0 | 0.0 | 0.0 | 0.597 |
| C. elegans | 0.792 | 0.941 | 0.978 | 0.923 | 0.22 | 0.0 | -0.0 | 0.0 | 0.0 | 0.147 |

## Names

No synonyms found

## Roles

ChEBI entry None has no roles

## Status

| | |
|---|---|
| Approved drug (according to ChEMBL) | No |
| Number of Rule of 5 violations | 0 |
| Binding affinity to original target in log units (RF-Score prediction) | 7.48 |
| Burns *C. elegans* bioavailability prediction | -4.28 |

## Compound Target Characteristics

### Alcohol dehydrogenase class-3

Best gene implication in ageing for this target family came from gene P12711 annotated in UniProt release 2014_02. Annotation GO 7568 (aging) was Inferred from Expression Pattern

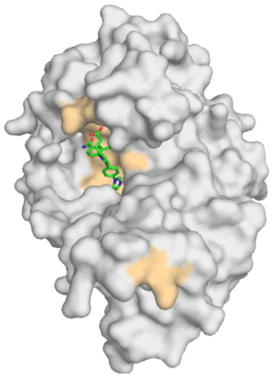

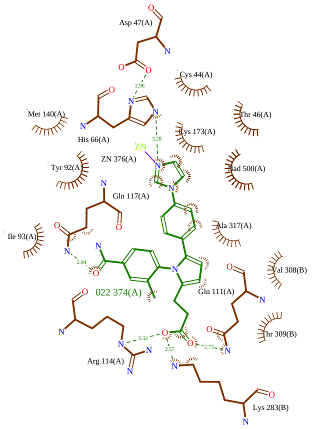


| protein | amino acids contacts (binding site) | | | | | | | | | | | | |
|---|---|---|---|---|---|---|---|---|---|---|---|---|---|
| PDB:3qj5:chainA:P11766 | C | T | H | Y | I | Q | R | Q | M | C | K | T | A |
| tr:Q6IRT1:Q6IRT1_HUMAN | C | T | H | Y | I | Q | R | Q | M | C | K | T | A |
| sp:P11766:ADHX_HUMAN | C | T | H | Y | I | Q | R | Q | M | C | K | T | A |
| sp:P12711:ADHX_RAT | C | T | H | Y | I | Q | R | Q | M | C | K | T | A |
| sp:P28474:ADHX_MOUSE | C | T | H | Y | I | Q | R | Q | M | C | K | T | A |
| tr:Q6P5I3:Q6P5I3_MOUSE | C | T | H | Y | I | Q | R | Q | M | C | K | T | A |
| sp:P46415:ADHX_DROME | C | T | H | Y | I | Q | R | Q | M | C | K | V | A |
| sp:Q17335:ADHX_CAEEL | C | T | H | Y | V | Q | R | Q | M | C | K | T | A |
| tr:Q965R0:Q965R0_CAEEL | C | T | H | Y | V | Q | R | Q | M | C | K | T | A |
| sp:P32771:FADH_YEAST | C | T | H | Y | T | G | R | Q | M | C | K | T | A |

| | whole protein | | domain-based | | contact-based | |
|---|---|---|---|---|---|---|
| protein | ident | simil | ident | simil | ident | simil |
| PDB:3qj5:chainA:P11766 | 1.0 | 1.0 | 1.0 | 1.0 | 1.0 | 1.0 |
| tr:Q6IRT1:Q6IRT1_HUMAN | 1.0 | 1.0 | 1.0 | 1.0 | 1.0 | 1.0 |
| sp:P11766:ADHX_HUMAN | 1.0 | 1.0 | 1.0 | 1.0 | 1.0 | 1.0 |
| sp:P12711:ADHX_RAT | 0.94 | 0.98 | 0.95 | 0.98 | 1.0 | 1.0 |
| sp:P28474:ADHX_MOUSE | 0.93 | 0.98 | 0.93 | 0.98 | 1.0 | 1.0 |
| tr:Q6P5I3:Q6P5I3_MOUSE | 0.93 | 0.98 | 0.93 | 0.98 | 1.0 | 1.0 |
| sp:P46415:ADHX_DROME | 0.7 | 0.9 | 0.72 | 0.91 | 0.92 | 0.95 |
| sp:Q17335:ADHX_CAEEL | 0.67 | 0.88 | 0.7 | 0.9 | 0.92 | 0.98 |
| tr:Q965R0:Q965R0_CAEEL | 0.43 | 0.59 | 0.42 | 0.57 | 0.92 | 0.98 |
| sp:P32771:FADH_YEAST | 0.6 | 0.84 | 0.63 | 0.87 | 0.85 | 0.86 |

### Fdh (FBgn0011768) associated phenotypes

heat sensitive, memory defective

(Information from FlyBase)

### Fdh (UniProt:P46415) annotation

**Function**: Class-III ADH is remarkably ineffective in oxidizing ethanol, but it readily catalyzes the oxidation of long-chain primary alcohols and the oxidation of S-(hydroxymethyl) glutathione.
**Cofactor**:Zn(2+)Note=Binds 2 Zn(2+) ions per subunit. ;

(Information from UniProt)

### H24K24.3 (UniProt:Q17335) annotation

**Function**: Class-III ADH is remarkably ineffective in oxidizing ethanol, but it readily catalyzes the

oxidation of long-chain primary alcohols and the oxidation of S-(hydroxymethyl) glutathione.
**Cofactor**: Zn(2+)Note=Binds 2 Zn(2+) ions per subunit. ;
**Subunit**: Homodimer.
**Subcellular location**: Cytoplasm

(Information from UniProt)

**CELE**$_{Y}50D4C.2(UniProt : Q965R0)annotation$
**Cofactor**: Zn(2+) Evidence=(RuleBase:RU361277);

(Information from UniProt)
