# Supplementary material for: Drug repurposing for aging research using model organisms
Source: Aging Cell. 2017 Jun 16;16(5):1006–15. doi: 10.1111/acel.12626 (PMC5595691; doi:10.1111/acel.12626)
Supplement: Supplementary file 7 — Data S1 Zip‐Archive of all report cards. [file ACEL-16-1006-s007.zip › RC_039.pdf]

# 039

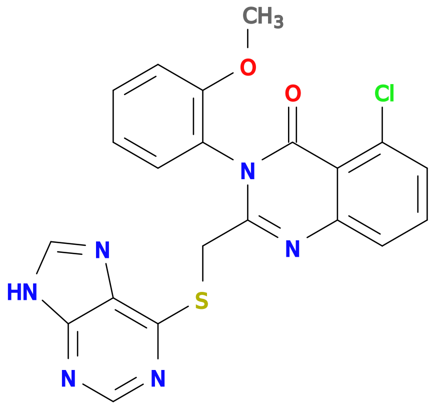

**Database identifiers**

| | |
|---|---|
| ChEMBLCompound | CHEMBL1213083 |
| DrugBank | DB06831 |

## Ranking

| | Rank | Score |
|---|---|---|
| Drosophila | 156/697 | 0.744 |
| C. elegans | 114/591 | 0.31 |

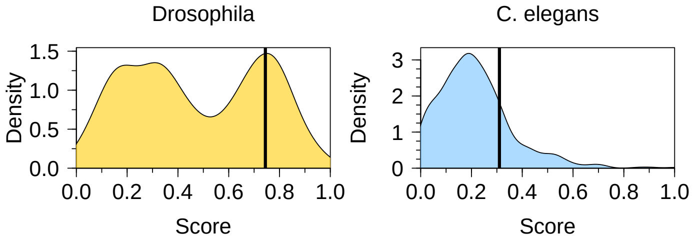


| | Ageing implication | Domain conservation | Binding site conservation | Binding affinity | Bioavailability | Lipinski | Promiscuity | Purchasability | Drug approval | **Total** |
|---|---|---|---|---|---|---|---|---|---|---|
| Drosophila | 1.0 | 0.913 | 0.859 | 0.949 | (0.9) | 0.0 | -0.0 | 0.0 | 0.075 | 0.744 |
| C. elegans | 1.0 | 0.761 | 0.754 | 0.949 | 0.433 | 0.0 | -0.0 | 0.0 | 0.075 | 0.31 |

## Names

No synonyms found

## Roles

ChEBI entry None has no roles

## Status

| | |
|---|---|
| Approved drug (according to ChEMBL) | No |
| Classification (according to DrugBank) | experimental |
| Number of Rule of 5 violations | 0 |
| Binding affinity to original target in log units (RF-Score prediction) | 7.92 |
| Burns *C. elegans* bioavailability prediction | 2.0 |

## Compound Target Characteristics

### Phosphatidylinositol 4,5-bisphosphate 3-kinase catalytic subunit delta isoform

Best gene implication in ageing for this target family came from gene Q94125 annotated in UniProt release 2014_02. Annotation GO 8340 (determination of adult lifespan) was Inferred from Mutant Phenotype

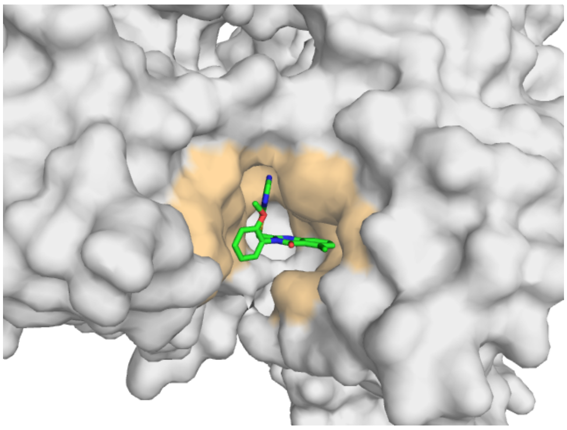

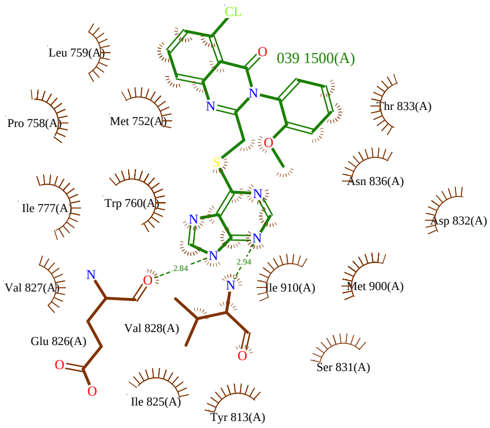


| protein | amino acids contacts (binding site) |
|---|---|
| PDB:2wxf:chainA:O35904 | T F M P L W I Y I E V V S D T N M I |
| tr:B7ZM44:B7ZM44_HUMAN | T F M P L W I Y I E V V S D T N M I |
| sp:O00329:PK3CD_HUMAN | T F M P L W I Y I E V V S D T N M I |
| tr:Q5SR50:Q5SR50_HUMAN | T F M P L W I Y I E V V S D T N M I |
| tr:D4A5Q1:D4A5Q1_RAT | T F M P L W I Y I E V V S D T N M I |
| tr:Q3TBW3:Q3TBW3_MOUSE | T F M P L W I Y I E V V S D T N M I |
| tr:Q3T9Y0:Q3T9Y0_MOUSE | T F M P L W I Y I E V V S D T N M I |
| sp:O35904:PK3CD_MOUSE | T F M P L W I Y I E V V S D T N M I |
| tr:Q3UDT3:Q3UDT3_MOUSE | T F M P L W I Y I E V V S D T N M I |
| tr:Q8CI98:Q8CI98_MOUSE | T F M P L W I Y I E V V S D T N M I |
| tr:B0QZL5:B0QZL5_MOUSE | T F M P L W I Y I E V V S D T N M I |
| tr:P91634:P91634_DROME | K V M P L W I Y I E V V A E T N M I |
| tr:H1ZY88:H1ZY88_DROME | K V M P L W I Y I E V V A E T N M I |
| tr:H1ZY91:H1ZY91_DROME | K V M P L W I Y I E V V A E T N M I |
| sp:Q94125:AGE1_CAEEL | I V L P L M I Y I E V V C K T E M I |

| protein | whole protein ident | simil | domain-based ident | simil | contact-based ident | simil |
|---|---|---|---|---|---|---|
| PDB:2wxf:chainA:O35904 | 1.0 | 1.0 | 1.0 | 1.0 | 1.0 | 1.0 |
| tr:B7ZM44:B7ZM44_HUMAN | 0.95 | 0.98 | 0.97 | 0.99 | 1.0 | 1.0 |
| sp:O00329:PK3CD_HUMAN | 0.95 | 0.98 | 0.97 | 0.99 | 1.0 | 1.0 |
| tr:Q5SR50:Q5SR50_HUMAN | 0.86 | 0.9 | 0.97 | 0.99 | 1.0 | 1.0 |
| tr:D4A5Q1:D4A5Q1_RAT | 0.89 | 0.9 | 0.9 | 0.91 | 1.0 | 1.0 |
| tr:Q3TBW3:Q3TBW3_MOUSE | 1.0 | 1.0 | 1.0 | 1.0 | 1.0 | 1.0 |
| tr:Q3T9Y0:Q3T9Y0_MOUSE | 1.0 | 1.0 | 1.0 | 1.0 | 1.0 | 1.0 |
| sp:O35904:PK3CD_MOUSE | 1.0 | 1.0 | 1.0 | 1.0 | 1.0 | 1.0 |
| tr:Q3UDT3:Q3UDT3_MOUSE | 1.0 | 1.0 | 1.0 | 1.0 | 1.0 | 1.0 |
| tr:Q8CI98:Q8CI98_MOUSE | 1.0 | 1.0 | 1.0 | 1.0 | 1.0 | 1.0 |
| tr:B0QZL5:B0QZL5_MOUSE | 1.0 | 1.0 | 1.0 | 1.0 | 1.0 | 1.0 |
| tr:P91634:P91634_DROME | 0.37 | 0.75 | 0.52 | 0.84 | 0.78 | 0.86 |
| tr:H1ZY88:H1ZY88_DROME | 0.37 | 0.75 | 0.52 | 0.84 | 0.78 | 0.86 |
| tr:H1ZY91:H1ZY91_DROME | 0.37 | 0.75 | 0.52 | 0.83 | 0.78 | 0.86 |
| sp:Q94125:AGE1_CAEEL | 0.23 | 0.62 | 0.34 | 0.72 | 0.61 | 0.75 |

**Pi3K92E (FBgn0015279) associated phenotypes**

RU486 conditional, cell autonomous, cell death defective, cell growth defective, cell non-autonomous, cell size defective, chemical resistant, chemical sensitive, decreased cell death, decreased cell growth, decreased cell number, decreased cell size, developmental rate defective, feeding behavior defective, heat sensitive, hyperplasia, increased cell growth, increased cell number, increased cell size, large body, locomotor behavior defective, long lived, neuroanatomy defective, neurophysiology defective, nutrition conditional, partially lethal - majority die, photoperiod response variant, planar polarity defective, short lived, size defective, small body, smell perception defective, somatic clone, some die during pupal stage, starvation stress response defective, stress response defective, wound healing defective

(Information from FlyBase)

**age-1 (WBGene00000090) associated phenotypes**

DMPP resistant, aging variant, brood size variant, cadmium resistant, dauer arrest variant, dauer constitutive, egg laying variant, extended life span, failure to hatch, fat content increased, hermaphrodite fertility variant, larval lethal, late stage egg laid, life span variant, organism physiology variant, organism temperature response variant, paraquat resistant, reproductive longevity extended, scrawny, sterile, transgene subcellular localization variant

(Information from WormBase)

**age-1 (UniProt:Q94125) annotation**

**Function**: Phosphatidylinositol 3-kinase homolog that regulates longevity and diapause. Could function in the development or neuroendocrine signaling of the dauer pathway (PubMed:8700226). Mediates susceptibility to enteropathogenic E.coli infection (PubMed:16091039). (PubMed:16091039, PubMed:8700226).
**Developmental stage**: Expressed both maternally and zygotically. (PubMed:8700226).

(Information from UniProt)
