# Supplementary material for: Drug repurposing for aging research using model organisms
Source: Aging Cell. 2017 Jun 16;16(5):1006–15. doi: 10.1111/acel.12626 (PMC5595691; doi:10.1111/acel.12626)
Supplement: Supplementary file 7 — Data S1 Zip‐Archive of all report cards. [file ACEL-16-1006-s007.zip › RC_0DU.pdf]

0DU

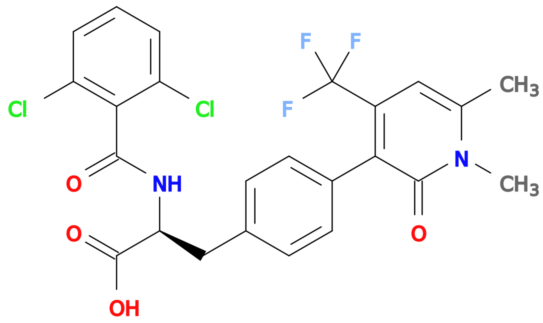

Database identifiers
ChEMBLCompound CHEMBL2332742

Ranking

	Rank	Score
Drosophila	348/697	0.432
C. elegans	503/591	0.067

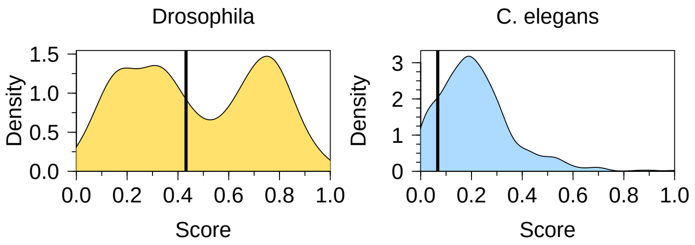

	Ageing implication	Domain conservation	Binding site conservation	Binding affinity	Bioavailability	Lipinski	Promiscuity	Purchasability	Drug approval	**Total**
Drosophila	1.0	0.905	0.742	0.88	(0.9)	-0.1	-0.0	0.0	0.0	0.432
C. elegans	1.0	0.894	0.908	0.88	0.235	-0.1	-0.0	0.0	0.0	0.067

Names

No synonyms found

Roles

ChEBI entry None has no roles

Status

Approved drug (according to ChEMBL)	No
Number of Rule of 5 violations	2
Binding affinity to original target in log units (RF-Score prediction)	6.99
Burns *C. elegans* bioavailability prediction	-2.96

Compound Target Characteristics

Integrin beta-7

Best gene implication in ageing for this target family came from gene P11584 annotated in UniProt release 2014_02. Annotation GO 8340 (determination of adult lifespan) was Inferred from Mutant Phenotype

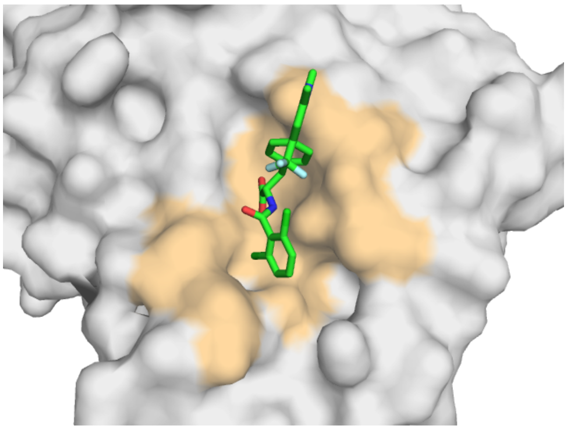

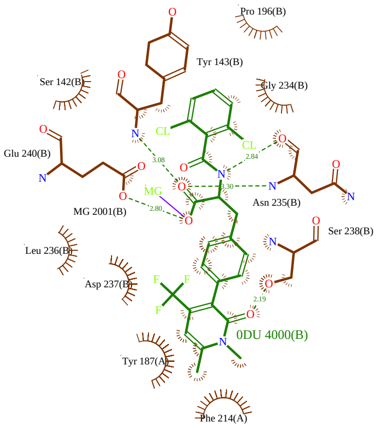

protein	amino acids contacts (binding site)
PDB:3v4v:chainB:P26010	S Y P P G N L D S E
tr:F5H6T4:F5H6T4_HUMAN	S Y P P G N L D S E
tr:B7Z506:B7Z506_HUMAN	S Y P P G N L D S E
sp:P26010:ITB7_HUMAN	S Y P P G N L D S E
tr:G3V7M2:G3V7M2_RAT	S Y P P G N L D S E
sp:P26011:ITB7_MOUSE	S Y P P G N L D S E
sp:P11584:ITBX_DROME	S K P E G N L D A E
sp:Q27874:PAT3_CAEEL	S Y P P G N L D A E

	whole protein		domain-based		contact-based	
protein	ident	simil	ident	simil	ident	simil
PDB:3v4v:chainB:P26010	1.0	1.0	1.0	1.0	1.0	1.0
tr:F5H6T4:F5H6T4_HUMAN	0.56	0.58	1.0	1.0	1.0	1.0
tr:B7Z506:B7Z506_HUMAN	0.78	0.78	0.96	0.96	1.0	1.0
sp:P26010:ITB7_HUMAN	1.0	1.0	1.0	1.0	1.0	1.0
tr:G3V7M2:G3V7M2_RAT	0.85	0.95	0.92	0.98	1.0	1.0
sp:P26011:ITB7_MOUSE	0.85	0.95	0.94	0.98	1.0	1.0
sp:P11584:ITBX_DROME	0.31	0.67	0.5	0.83	0.7	0.74
sp:Q27874:PAT3_CAEEL	0.35	0.74	0.47	0.81	0.9	0.91

mys (FBgn0004657) associated phenotypes

cell shape defective, flightless, germline clone, heat sensitive, lethal - all die before end of first instar larval stage, lethal - all die before end of pupal stage, neuroanatomy defective, neurophysiology defective, partially, partially lethal - majority die, reduced, rescuable maternal effect, smell perception defective, somatic clone, some die during pupal stage

(Information from FlyBase)

mys (UniProt:P11584) annotation

Function: Integrin alpha-PS1/beta-PS is a receptor for laminin. Integrin alpha-PS2/beta-PS is a receptor for Tig, wb and Ten-m. Contributes to endodermal integrity and adhesion between the midgut epithelium and the surrounding visceral muscle. Essential for migration of the primordial midgut cells and for maintaining, but not establishing, cell polarity in the midgut epithelium. The two beta subunits mediate midgut migration by distinct mechanisms: beta-PS requires rhea/talin and Itgbetanu does not. Required for rhea/talin correct cellular localization in the midgut. Required for many embryonic (dorsal closure and somatic muscle attachments) and post-embryonic developmental processes (attachment between cell layers of imaginal

disks, organization of ommatidial arrays and flight muscle development). Involved in the function and/or development of the olfactory system. Plays a role in timely border cell migration during oogenesis. (PubMed:10821184, PubMed:15469969, PubMed:19035354, PubMed:7924982, PubMed:7972082, PubMed:8119134, PubMed:9660786).
Subunit: Heterodimer of an alpha and a beta subunit. Beta-PS associates with either alpha-PS1, alpha-PS2, alpha-PS3, alpha-PS4 or alpha-PS5.
Subcellular location: Apical cell membrane (PubMed:19035354); Single-pass type I membrane protein (PubMed:19035354). Lateral cell membrane (PubMed:19035354); Single-pass type I membrane protein (PubMed:19035354). Basal cell membrane (PubMed:19035354); Single-pass type I membrane protein (PubMed:19035354). Note=In ovary, localizes to the apical, lateral and basal membranes of follicle cells through oogenesis stage 10A. Apical membrane expression peaks at oogenesis stages 9 and 10A in columnar follicle cells overlying the oocyte but decreases in the most posterior follicle cells. Thereafter, it is down-regulated. Localization to lateral and basal membranes persists during dorsal appendage morphogenesis.
Tissue specificity: In ovaries, strongly expressed in follicle cells. In oocytes, expressed in the forming dorsal appendages (at protein level). Expressed in the embryonic dorsal cuticle, the larval eye and the wing imaginal disk. (PubMed:19035354, PubMed:8119134).
Disruption phenotype: In zygotic mutant embryos, midgut forms primary constrictions but fails to elongate and the visceral muscle does not flatten but remains attached to the midgut epithelium. Embryos lacking maternal and zygotic mys show a delay in midgut migration. Mutant larvae present an olfatory phenotype, showing reduced response to isoamyl acetate but normal response to ethyl acetate. (PubMed:10821184, PubMed:15469969).
Miscellaneous: The absence of the beta-PS subunit results in detachment and rounding up of the muscles, thus the gene encoding beta-PS is called myospheroid.

(Information from UniProt)

pat-3 (WBGene00003930) associated phenotypes

body wall muscle actin organization defective, body wall muscle cell polarization variant, body wall muscle myosin organization defective, cell migration variant, embryonic lethal, excretory canal short, paralyzed arrested elongation two fold, protein expression absent, sterile

(Information from WormBase)

pat-3 (UniProt:Q27874) annotation

Function: Possible role in cell-cell interactions. Integrin alpha pat-1/beta pat-3 is a receptor for laminin. Integrin alpha pat- 2/beta pat-3 recognizes the sequence R-G-D in its ligands (By similarity). During gonad morphogenesis, involved in distal tip cell (DTC)-mediated guidance of gonad elongation, in maintaining their sharp tapering morphology and in their migration (PubMed:19023419). (, PubMed:19023419).
Subunit: Heterodimer of an alpha and a beta subunit. Beta pat-3 associates with either alpha pat-1 or alpha pat-2.
Subcellular location: Membrane ; Single-pass type I membrane protein
Disruption phenotype: RNAi-mediated knockdown in distal tip cell (DTC) causes DTC migration and guidance defects during the second phase of gonad elongation resulting in a triangular shaped gonad. (PubMed:19023419).

(Information from UniProt)
