# Supplementary material for: Drug repurposing for aging research using model organisms
Source: Aging Cell. 2017 Jun 16;16(5):1006–15. doi: 10.1111/acel.12626 (PMC5595691; doi:10.1111/acel.12626)
Supplement: Supplementary file 7 — Data S1 Zip‐Archive of all report cards. [file ACEL-16-1006-s007.zip › RC_0J8.pdf]

# 0J8

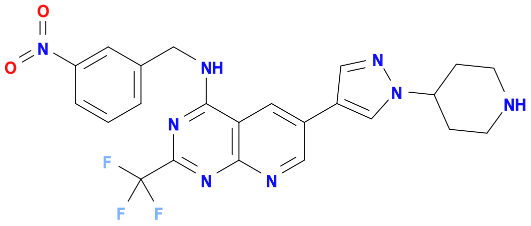

**Database identifiers**
ChEMBLCompound CHEMBL2148143

## Ranking

| | Rank | Score |
|---|---|---|
| Drosophila | NA | NA |
| C. elegans | 535/591 | 0.04 |

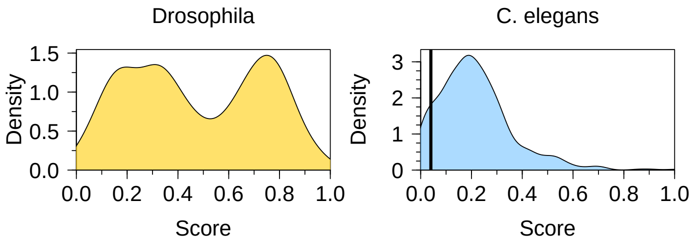


| | Ageing implication | Domain conservation | Binding site conservation | Binding affinity | Bioavailability | Lipinski | Promiscuity | Purchasability | Drug approval | **Total** |
|---|---|---|---|---|---|---|---|---|---|---|
| Drosophila | NA | NA | NA | NA | NA | NA | NA | NA | NA | NA |
| C. elegans | 0.624 | 0.853 | 0.546 | 0.95 | 0.328 | -0.05 | -0.0 | 0.0 | 0.0 | 0.04 |

## Names

No synonyms found

## Roles

ChEBI entry None has no roles

## Status

| | |
|---|---|
| Approved drug (according to ChEMBL) | No |
| Number of Rule of 5 violations | 1 |
| Binding affinity to original target in log units (RF-Score prediction) | 7.95 |
| Burns *C. elegans* bioavailability prediction | 0.26 |

## Compound Target Characteristics

### Hepatocyte growth factor receptor

Best gene implication in ageing for this target family came from gene Q2IBC7 via mapping the annotation from RGD 3082 annotated in RGD 2014-03-11. Annotation GO subterm of 7568 (aging)

was Inferred from Expression Pattern

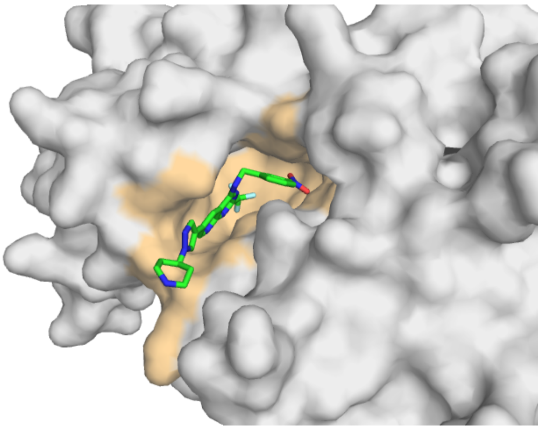

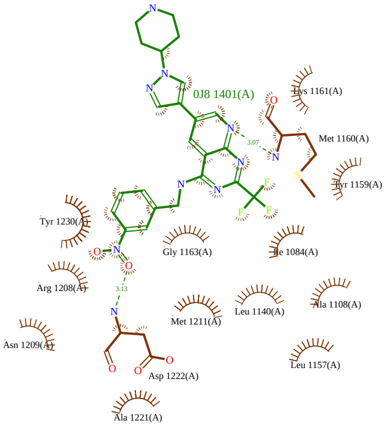


| protein | amino acids contacts (binding site) | | | | | | | | | | | | | | | |
|---|---|---|---|---|---|---|---|---|---|---|---|---|---|---|---|---|
| PDB:4gg7:chainA:P08581 | I | A | L | L | P | Y | M | K | G | R | N | M | A | D | A | Y |
| tr:B4DLF5:B4DLF5_HUMAN | I | A | L | L | P | Y | M | K | G | R | N | M | A | D | A | Y |
| sp:P08581:MET_HUMAN | I | A | L | L | P | Y | M | K | G | R | N | M | A | D | A | Y |
| tr:Q2IBC7:Q2IBC7_RAT | I | A | L | L | P | Y | M | K | G | R | N | M | A | D | A | Y |
| tr:F8VQL0:F8VQL0_MOUSE | I | A | L | L | P | Y | M | K | G | R | N | M | A | D | A | Y |
| tr:Q6AHP3:Q6AHP3_CAEEL | I | V | L | T | E | Y | M | A | G | R | N | L | A | D | C | - |
| tr:H1AGA1:H1AGA1_CAEEL | I | V | L | T | E | Y | M | A | G | R | N | L | A | D | C | - |
| tr:H2KZU7:H2KZU7_CAEEL | I | V | L | T | E | Y | M | A | G | R | N | L | A | D | C | - |

| | whole protein | | domain-based | | contact-based | |
|---|---|---|---|---|---|---|
| protein | ident | simil | ident | simil | ident | simil |
| PDB:4gg7:chainA:P08581 | 1.0 | 1.0 | 1.0 | 1.0 | 1.0 | 1.0 |
| tr:B4DLF5:B4DLF5_HUMAN | 0.69 | 0.69 | 1.0 | 1.0 | 1.0 | 1.0 |
| sp:P08581:MET_HUMAN | 1.0 | 1.0 | 1.0 | 1.0 | 1.0 | 1.0 |
| tr:Q2IBC7:Q2IBC7_RAT | 0.88 | 0.96 | 0.98 | 1.0 | 1.0 | 1.0 |
| tr:F8VQL0:F8VQL0_MOUSE | 0.89 | 0.96 | 0.99 | 1.0 | 1.0 | 1.0 |
| tr:Q6AHP3:Q6AHP3_CAEEL | 0.13 | 0.4 | 0.41 | 0.78 | 0.56 | 0.55 |
| tr:H1AGA1:H1AGA1_CAEEL | 0.13 | 0.4 | 0.41 | 0.78 | 0.56 | 0.55 |
| tr:H2KZU7:H2KZU7_CAEEL | 0.13 | 0.4 | 0.41 | 0.78 | 0.56 | 0.55 |
