# Supplementary material for: Drug repurposing for aging research using model organisms
Source: Aging Cell. 2017 Jun 16;16(5):1006–15. doi: 10.1111/acel.12626 (PMC5595691; doi:10.1111/acel.12626)
Supplement: Supplementary file 7 — Data S1 Zip‐Archive of all report cards. [file ACEL-16-1006-s007.zip › RC_0JJ.pdf]

0JJ

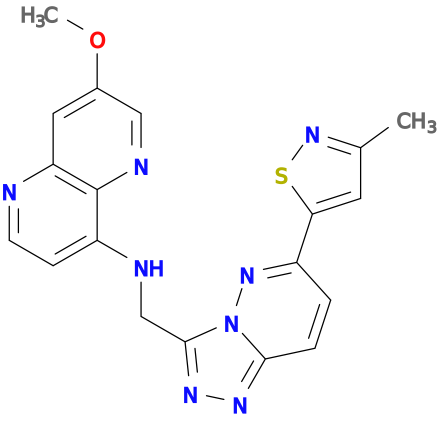

Database identifiers
ChEMBLCompound CHEMBL566685

Ranking

	Rank	Score
Drosophila	NA	NA
C. elegans	201/591	0.247

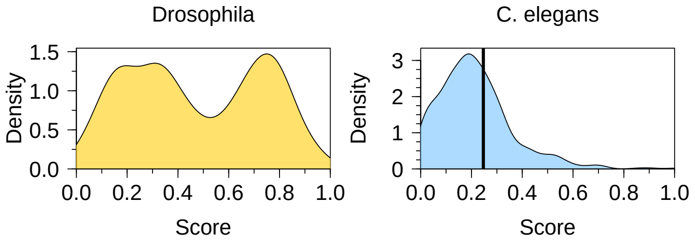

	Ageing implication	Domain conservation	Binding site conservation	Binding affinity	Bioavailability	Lipinski	Promiscuity	Purchasability	Drug approval	**Total**
Drosophila	NA	NA	NA	NA	NA	NA	NA	NA	NA	NA
C. elegans	0.624	0.853	0.573	0.947	0.855	0.0	-0.0	0.0	0.0	0.247

Names

No synonyms found

Roles

ChEBI entry None has no roles

Status

Approved drug (according to ChEMBL)	No
Number of Rule of 5 violations	0
Binding affinity to original target in log units (RF-Score prediction)	7.88
Burns *C. elegans* bioavailability prediction	7.4

Compound Target Characteristics

Hepatocyte growth factor receptor

Best gene implication in ageing for this target family came from gene Q2IBC7 via mapping the annotation from RGD 3082 annotated in RGD 2014-03-11. Annotation GO subterm of 7568 (aging) was Inferred from Expression Pattern

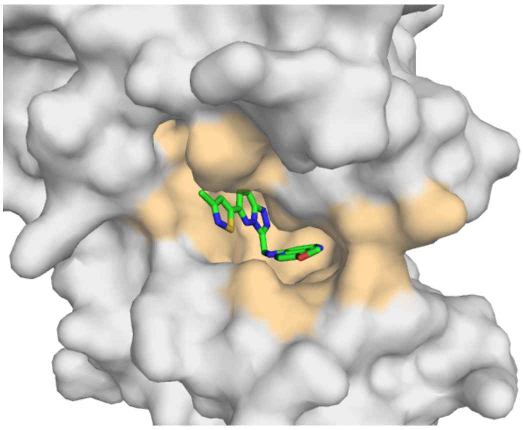

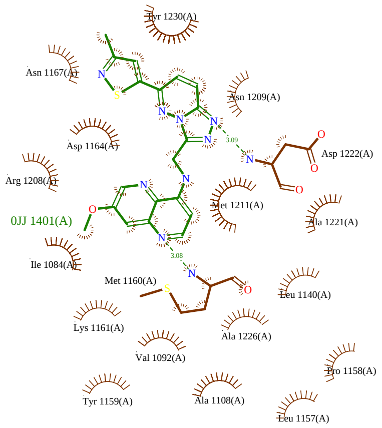

protein	amino acids contacts (binding site)
PDB:4deg:chainA:P08581	I V A L L P Y M K D N R N M A D A Y
tr:B4DLF5:B4DLF5_HUMAN	I V A L L P Y M K D N R N M A D A Y
sp:P08581:MET_HUMAN	I V A L L P Y M K D N R N M A D A Y
tr:Q2IBC7:Q2IBC7_RAT	I V A L L P Y M K D N R N M A D A Y
tr:F8VQL0:F8VQL0_MOUSE	I V A L L P Y M K D N R N M A D A Y
tr:Q6AHP3:Q6AHP3_CAEEL	I V V L T E Y M A D S R N L A D C -
tr:H1AGA1:H1AGA1_CAEEL	I V V L T E Y M A D S R N L A D C -
tr:H2KZU7:H2KZU7_CAEEL	I V V L T E Y M A D S R N L A D C -

	whole protein		domain-based		contact-based	
protein	ident	simil	ident	simil	ident	simil
PDB:4deg:chainA:P08581	1.0	1.0	1.0	1.0	1.0	1.0
tr:B4DLF5:B4DLF5_HUMAN	0.69	0.69	1.0	1.0	1.0	1.0
sp:P08581:MET_HUMAN	1.0	1.0	1.0	1.0	1.0	1.0
tr:Q2IBC7:Q2IBC7_RAT	0.88	0.96	0.98	1.0	1.0	1.0
tr:F8VQL0:F8VQL0_MOUSE	0.89	0.96	0.99	1.0	1.0	1.0
tr:Q6AHP3:Q6AHP3_CAEEL	0.13	0.4	0.41	0.78	0.56	0.57
tr:H1AGA1:H1AGA1_CAEEL	0.13	0.4	0.41	0.78	0.56	0.57
tr:H2KZU7:H2KZU7_CAEEL	0.13	0.4	0.41	0.78	0.56	0.57
