# Supplementary material for: Drug repurposing for aging research using model organisms
Source: Aging Cell. 2017 Jun 16;16(5):1006–15. doi: 10.1111/acel.12626 (PMC5595691; doi:10.1111/acel.12626)
Supplement: Supplementary file 7 — Data S1 Zip‐Archive of all report cards. [file ACEL-16-1006-s007.zip › RC_0LI.pdf]

0LI

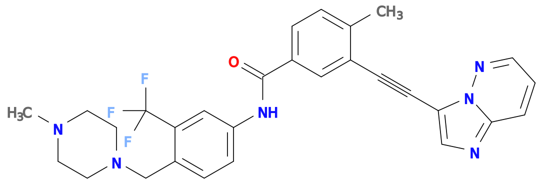

Database identifiers

ChEMBLCompound	CHEMBL1171837
DrugBank	DB08901
CHEBI	78543
ZINC	ZINC36701290
eMolecules	32176636

Ranking

	Rank	Score
Drosophila	470/697	0.306
C. elegans	189/591	0.254

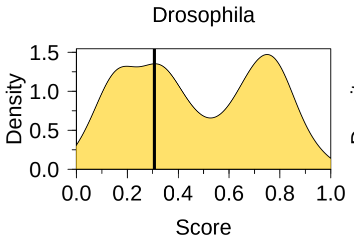

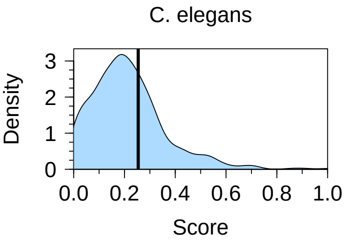

	Ageing implication	Domain conservation	Binding site conservation	Binding affinity	Bioavailability	Lipinski	Promiscuity	Purchasability	Drug approval	**Total**
Drosophila	0.203	0.966	0.95	0.935	(0.9)	-0.05	-0.0	0.1	0.1	0.306
C. elegans	0.203	0.952	0.941	0.935	0.617	-0.05	-0.0	0.1	0.1	0.254

Names

- **ponatinib**
- Ap 24534

Roles

antineoplastic agent, tyrosine kinase inhibitor

Status

Approved drug (according to ChEMBL)	Yes
Administration Route	Oral
Number of Rule of 5 violations	1
Binding affinity to original target in log units (RF-Score prediction)	7.66
Burns *C. elegans* bioavailability prediction	4.19

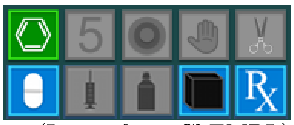

(Image from ChEMBL)

Medical Information

Indicatation: Ponatinib is indicated for the treatment of adult patients with chronic phase, accelerated phase, or blast phase chronic myeloid leukemia (CML) that is resistant or intolerant to prior tyrosine kinase inhibitor therapy or Philadelphia chromosome positive acute lymphoblastic

leukemia (Ph+ALL) that is resistant or intolerant to prior tyrosine kinase inhibitor therapy.

Mechanism of action: Ponatinib is a multi-target kinase inhibitor. Its primary cellular target is the Bcr-Abl tyrosine kinase protein which is constitutively active and promotes the progression of CML. This protein arises from the fused Bcr and Abl gene- what is commonly known as the Philadelphia chromosome. Ponatinib is unique in that it is especially useful in the treatment of resistant CML because it inhibits the tyrosine kinase activity of Abl and T315I mutant kinases. The T315I mutation confers resistance in cells as it prevents other Bcr-Abl inhibitors from binding to the Abl kinase. Other targets that ponatinib inhibits are members of the VEGFR, PDGFR, FGFR, EPH receptors and SRC families of kinases, and KIT, RET, TIE2, and FLT3. A decrease in tumour size expressing native or T315I mutant BCR-ABL have been observed in rats.

Toxicity: The most common non-hematologic adverse reactions (20%) were hypertension, rash, abdominal pain, fatigue, headache, dry skin, constipation, arthralgia, nausea, and pyrexia. Hematologic adverse reactions included thrombocytopenia, anemia, neutropenia, lymphopenia, and leukopenia.

Metabolism: At least 64% of a ponatinib dose undergoes phase I and phase II metabolism. CYP3A4 and to a lesser extent CYP2C8, CYP2D6 and CYP3A5 are involved in the phase I metabolism of ponatinib in vitro. Ponatinib is also metabolized by esterases and/or amidases.

(Information from DrugBank)

Compound Target Characteristics

Tyrosine-protein kinase ABL1

Best gene implication in ageing for this target family came from gene P00519 via mapping the annotation from Ensembl ENSG00000097007 via mapping the annotation from EntrezGene 25 via mapping the annotation from GenAgeHuman 0078 annotated in GenAge release 17.

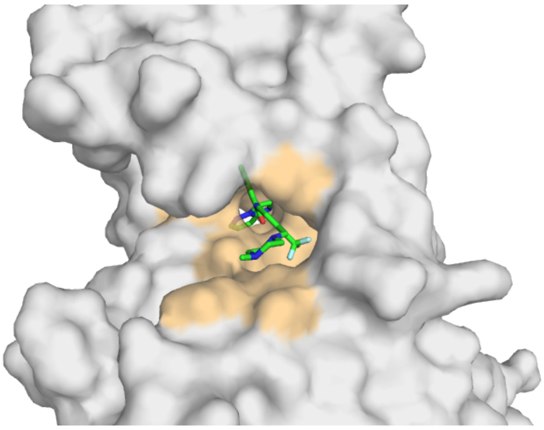

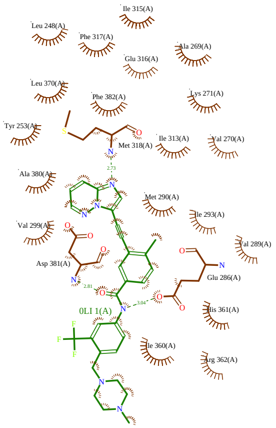

protein	amino acids contacts (binding site)
PDB:3ik3:chainA:P00520	L Y A V K E V M I L V I I E F M I H R L V A D F
PDB:3oxz:chainA:P00520	L Y A V K E V M I L V I T E F M I H R L V A D F
sp:P00519:ABL1_HUMAN	L Y A V K E V M I L V I T E F M I H R L V A D F
tr:Q59FK4:Q59FK4_HUMAN	L Y A V K E V M I L V I T E F M I H R L V A D F
tr:F1M0A6:F1M0A6_RAT	L Y A V K E V M I L V I T E F M I H R L V A D F
tr:D3ZGM3:D3ZGM3_RAT	L Y A V K E V M I L V I T E F M I H R L V A D F
tr:E9PT20:E9PT20_RAT	L Y A V K E V M I L V I T E F M I H R L V A D F
tr:Q3V2W1:Q3V2W1_MOUSE	L Y A V K E V M I L V I T E F M I H R L V A D F
sp:P00520:ABL1_MOUSE	L Y A V K E V M I L V I T E F M I H R L V A D F
tr:Q3SYK5:Q3SYK5_MOUSE	L Y A V K E V M I L V I T E F M I H R L V A D F
sp:P00522:ABL_DROME	L Y A V K E I M M L V I T E F M I H R L V A D F
tr:A8JNU2:A8JNU2_DROME	L Y A V K E I M M L V I T E F M I H R L V A D F
sp:P03949:ABL1_CAEEL	L Y A V K E I M L L V I T E F M I H R L I A D F

	whole protein		domain-based		contact-based	
protein	ident	simil	ident	simil	ident	simil
PDB:3ik3:chainA:P00520	0.99	1.0	1.0	1.0	1.0	1.0
PDB:3oxz:chainA:P00520	0.99	1.0	0.99	1.0	0.96	0.97
sp:P00519:ABL1_HUMAN	0.88	0.95	1.0	1.0	0.96	0.97
tr:Q59FK4:Q59FK4_HUMAN	0.83	0.92	1.0	1.0	0.96	0.97
tr:F1M0A6:F1M0A6_RAT	0.93	0.96	1.0	1.0	0.96	0.97
tr:D3ZGM3:D3ZGM3_RAT	0.96	0.99	1.0	1.0	0.96	0.97
tr:E9PT20:E9PT20_RAT	0.93	0.96	1.0	1.0	0.96	0.97
tr:Q3V2W1:Q3V2W1_MOUSE	0.33	0.33	1.0	1.0	0.96	0.97
sp:P00520:ABL1_MOUSE	1.0	1.0	1.0	1.0	0.96	0.97
tr:Q3SYK5:Q3SYK5_MOUSE	0.96	0.98	1.0	1.0	0.96	0.97
sp:P00522:ABL_DROME	0.26	0.51	0.78	0.93	0.88	0.95
tr:A8JNU2:A8JNU2_DROME	0.26	0.5	0.78	0.93	0.88	0.95
sp:P03949:ABL1_CAEEL	0.32	0.64	0.67	0.9	0.83	0.94

Abl (FBgn0000017) associated phenotypes

cell migration defective, cell shape defective, germline clone, heat sensitive, lethal - all die before end of pupal stage, maternal effect, mitotic cell cycle defective, neuroanatomy defective, neurophysiology defective, partially lethal - majority die, partially lethal - majority live, reduced, rescuable maternal effect, size defective, somatic clone, some die during pupal stage

(Information from FlyBase)

Abl (UniProt:P00522) annotation

Function: Arm and Abl proteins function cooperatively at adherens junctions in both the CNS and epidermis; critical for embryonic epithelial morphogenesis regulating cell shape changes and cell migration. Plays a critical role in transducing embryonic midline repulsive cues; may regulate cytoskeletal dynamics underlying a growth cone's response to midline cues. The ability of pCC/MP2 axons to correctly interpret midline repulsive cues and stay on the ipsilateral side is dependent on the strength of both Slit/robo and Abl-dependent signaling pathways. (PubMed:11756472, PubMed:12973825, PubMed:9635189).

Subcellular location: Cytoplasm.

Tissue specificity: Arm and ena colocalize with Abl at adherens junctions throughout development. (PubMed:11756472, PubMed:9635189).

Developmental stage: Expressed both maternally and zygotically. (PubMed:2832740).

Disruption phenotype: Both loss- and gain-of-function mutants exhibit neurons within the pCC/MP2 pathway that incorrectly project across the midline. Loss of Abl disrupts cell migration and cell shape changes during dorsal closure. (PubMed:11756472, PubMed:9635189).

(Information from UniProt)

abl-1 (WBGene00000018) associated phenotypes

apoptosis variant, gamma ray induced apoptosis increased, lipid synthesis increased, protein subcellular localization variant

(Information from WormBase)
