# Supplementary material for: Drug repurposing for aging research using model organisms
Source: Aging Cell. 2017 Jun 16;16(5):1006–15. doi: 10.1111/acel.12626 (PMC5595691; doi:10.1111/acel.12626)
Supplement: Supplementary file 7 — Data S1 Zip‐Archive of all report cards. [file ACEL-16-1006-s007.zip › RC_0RS.pdf]

0RS

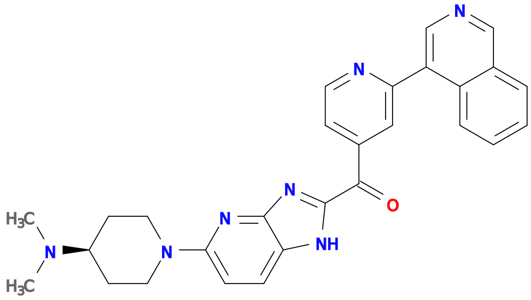

Database identifiers

ChEMBLCompound	CHEMBL2089064

Ranking

	Rank	Score
Drosophila	475/697	0.3
C. elegans	397/591	0.141

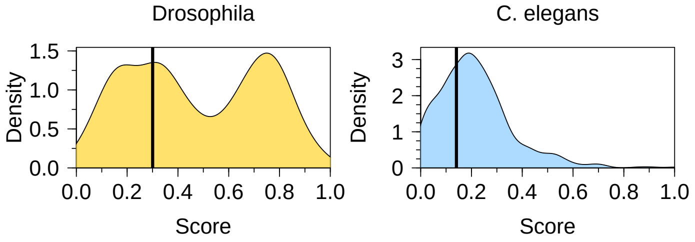

	Ageing implication	Domain conservation	Binding site conservation	Binding affinity	Bioavailability	Lipinski	Promiscuity	Purchasability	Drug approval	**Total**
Drosophila	0.436	0.904	0.924	0.918	(0.9)	0.0	-0.0	0.0	0.0	0.3
C. elegans	0.436	0.77	0.836	0.918	0.548	0.0	-0.0	0.0	0.0	0.141

Names

No synonyms found

Roles

ChEBI entry None has no roles

Status

Approved drug (according to ChEMBL)	No
Number of Rule of 5 violations	0
Binding affinity to original target in log units (RF-Score prediction)	7.42
Burns *C. elegans* bioavailability prediction	3.42

Compound Target Characteristics

Cyclin-dependent kinase 6

Best gene implication in ageing for this target family came from gene F1MA87 via mapping the annotation from RGD 621121 annotated in RGD 2014-03-11. Annotation GO subterm of 7568 (aging) was Inferred from Sequence Orthology

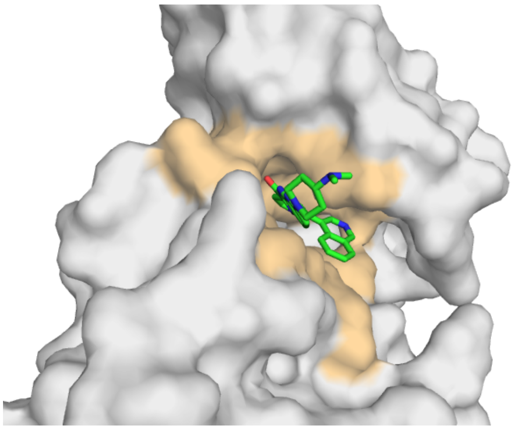

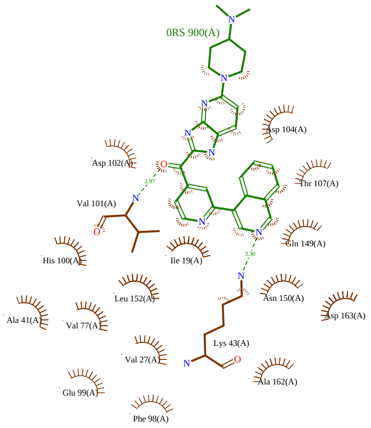

protein	amino acids contacts (binding site)
PDB:4ez5:chainA:Q00534	I V A K V F E H V D D T Q N L A D
sp:Q00534:CDK6_HUMAN	I V A K V F E H V D D T Q N L A D
tr:F1MA87:F1MA87_RAT	I V A K V F E H V D D T Q N L A D
sp:Q64261:CDK6_MOUSE	I V A K V F E H V D D T Q N L A D
tr:Q0VBK8:Q0VBK8_MOUSE	I V A K V F E H V D D T Q N L A D
tr:Q94877:Q94877_DROME	I V A K V F E H V E D D Q N L A D
tr:Q7K306:Q7K306_DROME	I V A K V F E H V E D D Q N L A D
tr:G5EFI3:G5EFI3_CAEEL	L V A K I M E R C D D T Q N L A D
tr:Q9XTR1:Q9XTR1_CAEEL	L V A K I M E R C D D T Q N L A D
sp:P00546:CDK1_YEAST	V V A K V F E F L D D R Q N L G D

	whole protein		domain-based		contact-based	
protein	ident	simil	ident	simil	ident	simil
PDB:4ez5:chainA:Q00534	1.0	1.0	1.0	1.0	1.0	1.0
sp:Q00534:CDK6_HUMAN	1.0	1.0	1.0	1.0	1.0	1.0
tr:F1MA87:F1MA87_RAT	0.97	0.99	0.98	0.99	1.0	1.0
sp:Q64261:CDK6_MOUSE	0.96	0.99	0.98	0.99	1.0	1.0
tr:Q0VBK8:Q0VBK8_MOUSE	0.96	0.99	0.98	0.99	1.0	1.0
tr:Q94877:Q94877_DROME	0.4	0.74	0.45	0.82	0.88	0.92
tr:Q7K306:Q7K306_DROME	0.4	0.74	0.45	0.82	0.88	0.92
tr:G5EFI3:G5EFI3_CAEEL	0.33	0.7	0.39	0.8	0.71	0.84
tr:Q9XTR1:Q9XTR1_CAEEL	0.28	0.61	0.35	0.72	0.71	0.84
sp:P00546:CDK1_YEAST	0.41	0.72	0.44	0.77	0.71	0.83

Cdk4 (FBgn0016131) associated phenotypes

decreased cell number, increased cell size, maternal effect, poor, radiation sensitive, size defective, small body, somatic clone, sterile

(Information from FlyBase)

cdk-4 (WBGene00000406) associated phenotypes

lethal, sterile

(Information from WormBase)

cdk-4 (UniProt:G5EFI3) annotation

Function: Serine/threonine-protein kinase which, in association with cyclin D-like protein cyd-1,

is required for the progression through the G1 phase of the cell cycle during postembryonic development by phosphorylating and inhibiting lin-35 and fzr-1 (PubMed:10518501, PubMed:11684669, PubMed:25562820). In complex with cyd-1, involved in sex determination during gonadogenesis by regulating the asymmetric division of the somatic gonadal precursor cell (SGP) (PubMed:16198291). (PubMed:10518501, PubMed:11684669, PubMed:16198291, PubMed:25562820).
Cofactor: Mg(2+) Evidence=(UniProtKB:P24941);
Subunit: Interacts with cyd-1; the interaction is likely involved in regulating cdk-4 activity. (PubMed:10518501).
Developmental stage: Expression initiates during mid-embryogenesis primarily in post-proliferative hypodermal cells and neurons in the head, ventral cord and tail, then declines until hatching where it is mainly seen in seam cells. Expressed throughout larval development in several blast cell linages. In the P lineage, expression is restricted to proliferating cells, whereas it persists in somatic gonads and seam cells. Expressed in uterus and intestine and to a lesser extent in spermatheca. (PubMed:10518501).
Disruption phenotype: RNAi-mediated knockdown results in L4 stage arrest which is associated with uncoordinated movements and a protruding vulva. Impaired cell division of P blast cells, somatic gonad precursors Z1 and Z4 and intestinal cells. Severe defect in the proliferation of P blast cells, intestinal cells, vulva cell precursors and somatic gonad precursors. Mesoblast M cell division is normal. (PubMed:10518501).

(Information from UniProt)

cdk-4 (UniProt:Q9XTR1) annotation
Function: Serine/threonine-protein kinase which, in association with cyclin D-like protein cyd-1, is required for the progression through the G1 phase of the cell cycle during postembryonic development by phosphorylating and inhibiting lin-35 and fzr-1 (PubMed:10518501, PubMed:11684669, PubMed:25562820). In complex with cyd-1, involved in sex determination during gonadogenesis by regulating the asymmetric division of the somatic gonadal precursor cell (SGP) (PubMed:16198291). (PubMed:10518501, PubMed:11684669, PubMed:16198291, PubMed:25562820).
Cofactor: Mg(2+) Evidence=(UniProtKB:P24941);
Subunit: Interacts with cyd-1; the interaction is likely involved in regulating cdk-4 activity. (PubMed:10518501).
Developmental stage: Expression initiates during mid-embryogenesis primarily in post-proliferative hypodermal cells and neurons in the head, ventral cord and tail, then declines until hatching where it is mainly seen in seam cells. Expressed throughout larval development in several blast cell linages. In the P lineage, expression is restricted to proliferating cells, whereas it persists in somatic gonads and seam cells. Expressed in uterus and intestine and to a lesser extent in spermatheca. (PubMed:10518501).
Disruption phenotype: RNAi-mediated knockdown results in L4 stage arrest which is associated with uncoordinated movements and a protruding vulva. Impaired cell division of P blast cells, somatic gonad precursors Z1 and Z4 and intestinal cells. Severe defect in the proliferation of P blast cells, intestinal cells, vulva cell precursors and somatic gonad precursors. Mesoblast M cell division is normal. (PubMed:10518501).

(Information from UniProt)
