# Supplementary material for: Drug repurposing for aging research using model organisms
Source: Aging Cell. 2017 Jun 16;16(5):1006–15. doi: 10.1111/acel.12626 (PMC5595691; doi:10.1111/acel.12626)
Supplement: Supplementary file 7 — Data S1 Zip‐Archive of all report cards. [file ACEL-16-1006-s007.zip › RC_0S7.pdf]

0S7

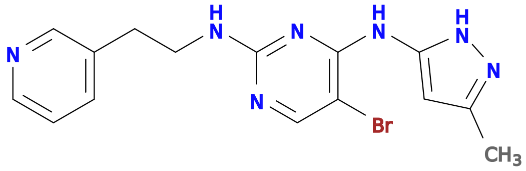

Database identifiers

ChEMBLCompound	CHEMBL2088089

Ranking

	Rank	Score
Drosophila	655/697	0.106
C. elegans	549/591	0.029

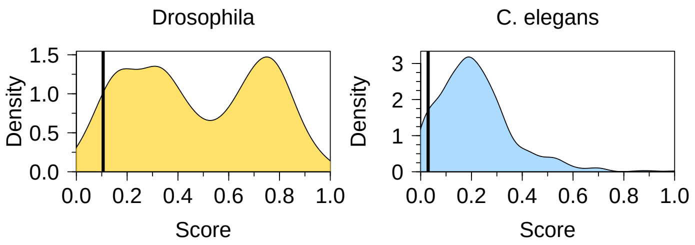

	Ageing implication	Domain conservation	Binding site conservation	Binding affinity	Bioavailability	Lipinski	Promiscuity	Purchasability	Drug approval	**Total**
Drosophila	0.203	0.91	0.943	0.678	(0.9)	0.0	-0.0	0.0	0.0	0.106
C. elegans	0.203	0.852	0.727	0.678	0.351	0.0	-0.0	0.0	0.0	0.029

Names

No synonyms found

Roles

ChEBI entry None has no roles

Status

Approved drug (according to ChEMBL)	No
Number of Rule of 5 violations	0
Binding affinity to original target in log units (RF-Score prediction)	5.75
Burns *C. elegans* bioavailability prediction	0.71

Compound Target Characteristics

Fibroblast growth factor receptor 1

Best gene implication in ageing for this target family came from gene P11362 via mapping the annotation from Ensembl ENSG00000077782 via mapping the annotation from EntrezGene 2260 via mapping the annotation from GenAgeHuman 0169 annotated in GenAge release 17.

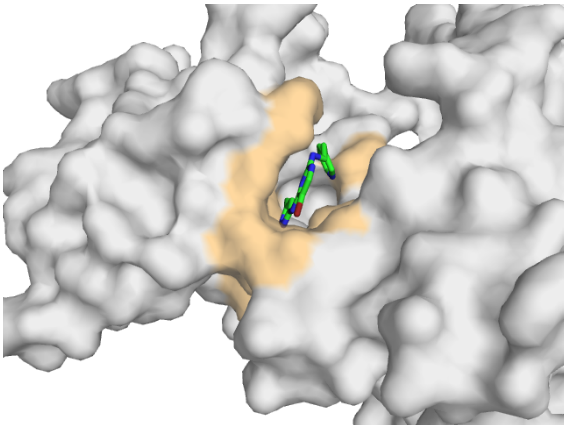

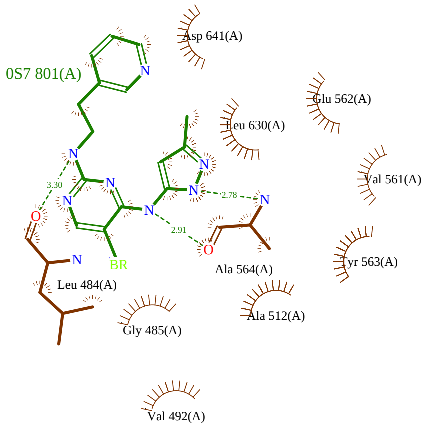

protein	amino acids contacts (binding site)
PDB:4f63:chainA:P11362	L G V A V E Y A S G L D
tr:D3DSX2:D3DSX2_HUMAN	L G V A V E Y A S G L D
tr:E7EU09:E7EU09_HUMAN	L G V A V E Y A S G L D
tr:J3KNT4:J3KNT4_HUMAN	L G V A V E Y A S G L D
sp:P11362:FGFR1_HUMAN	L G V A V E Y A S G L D
tr:Q63827:Q63827_RAT	L G V A V E Y A S G L D
tr:F1LM54:F1LM54_RAT	L G V A V E Y A S G L D
tr:Q8CBY7:Q8CBY7_MOUSE	L G V A V E Y A S G L D
sp:P16092:FGFR1_MOUSE	L G V A V E Y A S G L D
tr:Q8CIM9:Q8CIM9_MOUSE	L G V A V E Y A S G L D
tr:J3QN85:J3QN85_MOUSE	L G V A V E Y A S G L D
sp:Q07407:FGFR1_DROME	L G V A V E Y A P G L D
sp:Q09147:FGFR2_DROME	L G V A V E Y A P G L D
sp:Q10656:EGL15_CAEEL	L G V A V E L C K G L D

	whole protein		domain-based		contact-based	
protein	ident	simil	ident	simil	ident	simil
PDB:4f63:chainA:P11362	1.0	1.0	1.0	1.0	1.0	1.0
tr:D3DSX2:D3DSX2_HUMAN	0.47	0.47	1.0	1.0	1.0	1.0
tr:E7EU09:E7EU09_HUMAN	0.85	0.87	1.0	1.0	1.0	1.0
tr:J3KNT4:J3KNT4_HUMAN	0.96	0.97	1.0	1.0	1.0	1.0
sp:P11362:FGFR1_HUMAN	1.0	1.0	1.0	1.0	1.0	1.0
tr:Q63827:Q63827_RAT	0.88	0.88	1.0	1.0	1.0	1.0
tr:F1LM54:F1LM54_RAT	0.98	1.0	1.0	1.0	1.0	1.0
tr:Q8CBY7:Q8CBY7_MOUSE	0.42	0.42	1.0	1.0	1.0	1.0
sp:P16092:FGFR1_MOUSE	0.98	1.0	1.0	1.0	1.0	1.0
tr:Q8CIM9:Q8CIM9_MOUSE	0.98	0.99	1.0	1.0	1.0	1.0
tr:J3QN85:J3QN85_MOUSE	0.97	0.98	1.0	1.0	1.0	1.0
sp:Q07407:FGFR1_DROME	0.31	0.64	0.54	0.82	0.92	0.94
sp:Q09147:FGFR2_DROME	0.25	0.54	0.56	0.83	0.92	0.94
sp:Q10656:EGL15_CAEEL	0.25	0.56	0.5	0.78	0.75	0.73

htl (FBgn0010389) associated phenotypes

cell migration defective, increased cell death, increased cell number, neuroanatomy defective, somatic clone

(Information from FlyBase)

htl (UniProt:Q07407) annotation

Function: May be required for patterning of muscle precursor cells. May be essential for generation of mesodermal and endodermal layers, invaginations of various types of cells and CNS formation. (PubMed:8330538).

Subcellular location: Membrane; Single-pass type I membrane protein.

Tissue specificity: In early embryos, expression is specific to mesodermal primordium and invaginated mesodermal cells. At later stages, expression is seen in putative muscle precursor cells and in the CNS. (PubMed:8330538).

Developmental stage: Embryogenesis. (PubMed:8330538).
Caution: It is uncertain whether Met-1 or Met-16 is the initiator.

(Information from UniProt)

btl (FBgn0005592) associated phenotypes
cell migration defective, neuroanatomy defective, somatic clone

(Information from FlyBase)

btl (UniProt:Q09147) annotation
Function: May be required for patterning of muscle precursor cells: generation of mesodermal and endodermal layers, invaginations of various types of cells, and CNS formation. Essential for the ability of the migrating tracheal and midline cells to recognize external guiding cues. (PubMed:1325393, PubMed:1849109, PubMed:8330538).
Subcellular location: Membrane; Single-pass type I membrane protein.
Tissue specificity: During embryogenesis, expression is seen in mesoderm, endodermal precursor cells, CNS midline cells and trachea and salivary duct ectodermal cells. (PubMed:1325393, PubMed:1849109, PubMed:8330538).
Developmental stage: All stages of development. (PubMed:1849109).

(Information from UniProt)

egl-15 (WBGene00001184) associated phenotypes
L1 arrest, SM migration variant, axon midline crossing variant, bag of worms, bloated, egg laying defective, egg laying imipramine resistant, egg laying levamisole resistant, egg laying phentolamine resistant, egg laying serotonin resistant, egg laying variant, hermaphrodite sex muscle development variant, hermaphrodite sex muscle morphology variant, hermaphrodite sexual development variant, imipramine resistant, larval arrest, larval lethal, scrawny, serotonin resistant, ventral nerve cord maintenance variant

(Information from WormBase)

egl-15 (UniProt:Q10656) annotation
Function: Activation leads to the phosphorylation of SOC-1 on Tyr- 408 triggering PTP-2 recruitment and/or activation. Activates the SEM-5/SOS-1/RAS/MAPK pathway (PubMed:11689700). Acts in the hypodermis to regulate axon growth (PubMed:12835392). Activates protein degradation in muscles (PubMed:14517244). Plays a role in fluid homeostasis by acting in the hypodermis and also has a role in larval development (PubMed:7585964). Isoform b has no effect on axon growth but affects the maintenance of axon position. Isoform b interaction with egl-17 is required for the guidance of sex myoblast migration during gonad development (PubMed:12835392). Isoforms a, c, d and e interaction with let-756 appears to play a role in maintaining body morphology at higher temperatures (PubMed:12835392). (PubMed:11689700, PubMed:12835392, PubMed:14517244, PubMed:7585964).
Cofactor: Mn(2+)
Subcellular location: Membrane; Single-pass type I membrane protein.
Developmental stage: Expressed in the hypodermal membrane during the larval stage. (PubMed:11689700).
Ptm: Activity is regulated by the phosphatase clr-1, however it is not known whether clr-1 acts directly on egl-15. (PubMed:9585503).
Disruption phenotype: Early arrest in larval development. Impaired guided migration of sex myoblasts. (PubMed:7585964).

(Information from UniProt)
