# Supplementary material for: Drug repurposing for aging research using model organisms
Source: Aging Cell. 2017 Jun 16;16(5):1006–15. doi: 10.1111/acel.12626 (PMC5595691; doi:10.1111/acel.12626)
Supplement: Supplementary file 7 — Data S1 Zip‐Archive of all report cards. [file ACEL-16-1006-s007.zip › RC_10B.pdf]

10B

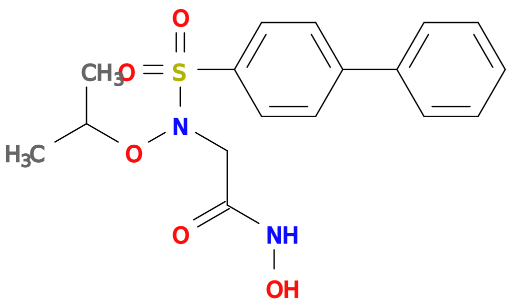

Database identifiers

ChEMBLCompound	CHEMBL181244
ZINC	ZINC03818628
eMolecules	29914335

Ranking

	Rank	Score
Drosophila	NA	NA
C. elegans	387/591	0.148

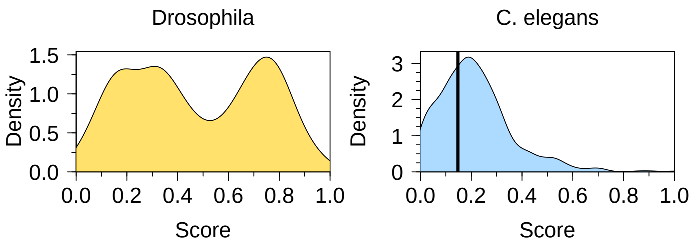

	Ageing implication	Domain conservation	Binding site conservation	Binding affinity	Bioavailability	Lipinski	Promiscuity	Purchasability	Drug approval	**Total**
Drosophila	NA	NA	NA	NA	NA	NA	NA	NA	NA	NA
C. elegans	0.624	0.493	0.876	0.863	0.207	0.0	-0.0	0.1	0.0	0.148

Names

- Boron, isotope of mass 10
- Boron-10

Roles

ChEBI entry None has no roles

Status

Approved drug (according to ChEMBL)	No
Number of Rule of 5 violations	0
Binding affinity to original target in log units (RF-Score prediction)	6.84
Burns *C. elegans* bioavailability prediction	-6.71

Compound Target Characteristics

Matrix metalloproteinase-9

Best gene implication in ageing for this target family came from gene D3ZYK8 via mapping the annotation from RGD 621320 annotated in RGD 2014-03-11. Annotation GO 7568 (aging) was Inferred from Expression Pattern

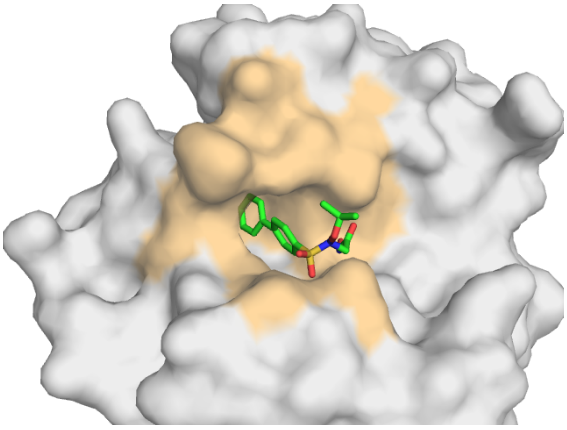

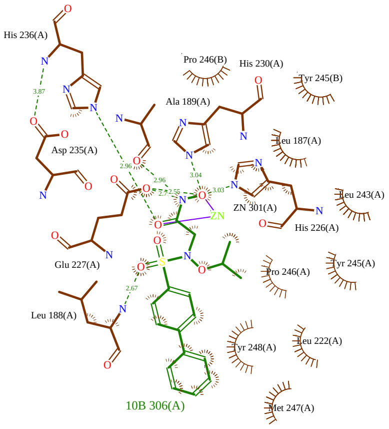

protein	amino acids contacts (binding site)
PDB:4h3x:chainA:P14780	G L L A L H E H H L Y P M Y
sp:P14780:MMP9_HUMAN	G L L A L H E H H L Y P M Y
tr:D3ZYK8:D3ZYK8_RAT	G L L A L H E H H L Y P M Y
sp:P41245:MMP9_MOUSE	G L L A L H E H H L Y P L Y
tr:O61265:O61265_CAEEL	G V L A Y H E H H L H P Y Y
tr:Q95X57:Q95X57_CAEEL	G V L A Y H E H H L H P Y Y
tr:O44836:O44836_CAEEL	G V L A Y H E H H L H P Y Y

	whole protein		domain-based		contact-based	
protein	ident	simil	ident	simil	ident	simil
PDB:4h3x:chainA:P14780	0.99	1.0	0.99	1.0	1.0	1.0
sp:P14780:MMP9_HUMAN	1.0	1.0	1.0	1.0	1.0	1.0
tr:D3ZYK8:D3ZYK8_RAT	0.75	0.92	0.84	0.95	1.0	1.0
sp:P41245:MMP9_MOUSE	0.72	0.89	0.82	0.94	0.93	0.99
tr:O61265:O61265_CAEEL	0.11	0.3	0.24	0.5	0.71	0.88
tr:Q95X57:Q95X57_CAEEL	0.12	0.34	0.25	0.57	0.71	0.88
tr:O44836:O44836_CAEEL	0.13	0.38	0.27	0.6	0.71	0.88

WBGene00019212 associated phenotypes

lethal, sterile

(Information from WormBase)
