# Supplementary material for: Drug repurposing for aging research using model organisms
Source: Aging Cell. 2017 Jun 16;16(5):1006–15. doi: 10.1111/acel.12626 (PMC5595691; doi:10.1111/acel.12626)
Supplement: Supplementary file 7 — Data S1 Zip‐Archive of all report cards. [file ACEL-16-1006-s007.zip › RC_16C.pdf]

16C

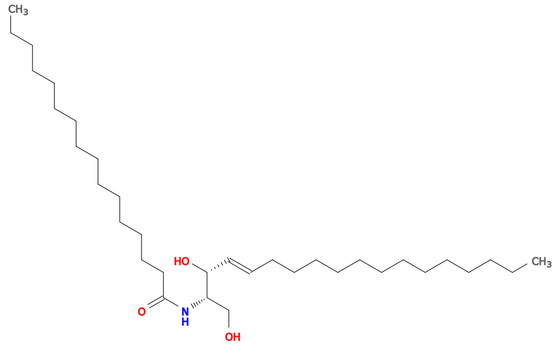

Database identifiers

ChEMBLCompound	CHEMBL35292
CHEBI	72959
ZINC	ZINC08860497
eMolecules	715863

Ranking

	Rank	Score
Drosophila	517/697	0.249
C. elegans	516/591	0.057

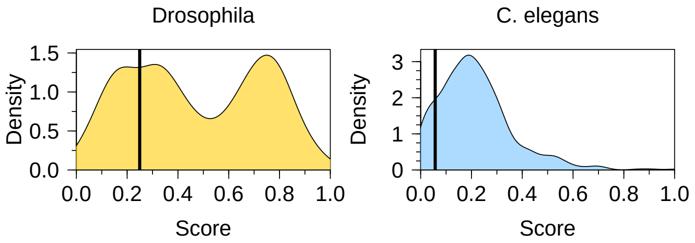

	Ageing implication	Domain conservation	Binding site conservation	Binding affinity	Bioavailability	Lipinski	Promiscuity	Purchasability	Drug approval	**Total**
Drosophila	0.36	0.897	0.889	0.967	(0.9)	-0.1	-0.0	0.1	0.0	0.249
C. elegans	0.36	0.87	0.857	0.967	0.222	-0.1	-0.0	0.1	0.0	0.057

Names

- **N-hexadecanoylsphingosine**
- C16 Cer
- C16 Ceramide
- C16-0(Palmitoyl)ceramide
- Cer(d18:1/16:0)
- Ceramide (d18:1/16:0)
- N-(hexadecanoyl)ceramide
- N-(hexadecanoyl)sphing-4-enine
- N-(palmitoyl)ceramide
- N-hexadecanoyl-D-erythro-sphingosine
- N-palmitoylsphing-4-enine
- N-Palmitoylsphingosine

Roles

ChEBI entry 72959 has no roles

Status

Approved drug (according to ChEMBL)	No
Number of Rule of 5 violations	2
Binding affinity to original target in log units (RF-Score prediction)	8.37
Burns *C. elegans* bioavailability prediction	-4.02

Compound Target Characteristics

Collagen type IV alpha-3-binding protein

Best gene implication in ageing for this target family came from gene Q9Y128 via mapping the annotation from Ensembl FBgn0027569 via mapping the annotation from EntrezGene 38928 via mapping the annotation from GenAgeModels 0117 annotated in GenAge release 17.

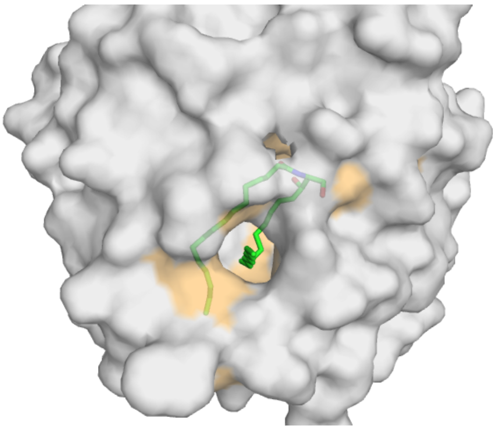

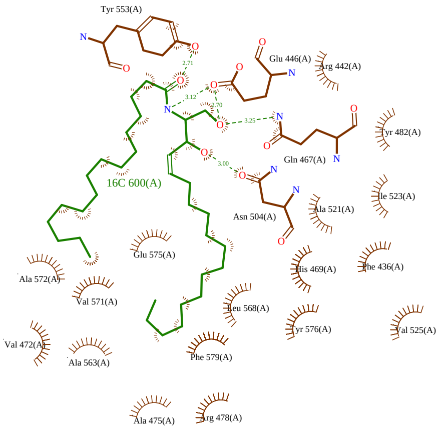

protein	amino acids contacts (binding site)
PDB:2e3o:chainA:Q9Y5P4	F R E Q H K V A R Y N A I V Y A L V A E Y F
PDB:2e3p:chainA:Q9Y5P4	F R E Q H K V A R Y N A I V Y A L V A E Y F
sp:Q9Y5P4:C43BP_HUMAN	F R E Q H K V A R Y N A I V Y A L V A E Y F
tr:D4ACN6:D4ACN6_RAT	F R E Q H K V A R Y N A I I Y A L V A E Y F
sp:Q9EQG9:C43BP_MOUSE	F R E Q H K V A R Y N A I I Y A L V A E Y F
tr:Q9Y128:Q9Y128_DROME	F R E Q H K I A R F N I L V Y A L V Y E Y F
tr:Q93569:Q93569_CAEEL	F K D Q H K V A R F N V L V Y V L V Y E Y F

	whole protein		domain-based		contact-based	
protein	ident	simil	ident	simil	ident	simil
PDB:2e3o:chainA:Q9Y5P4	0.99	1.0	0.99	1.0	1.0	1.0
PDB:2e3p:chainA:Q9Y5P4	0.98	0.99	0.99	1.0	1.0	1.0
sp:Q9Y5P4:C43BP_HUMAN	1.0	1.0	1.0	1.0	1.0	1.0
tr:D4ACN6:D4ACN6_RAT	0.96	0.99	0.98	1.0	0.95	0.99
sp:Q9EQG9:C43BP_MOUSE	0.97	0.99	0.98	1.0	0.95	0.99
tr:Q9Y128:Q9Y128_DROME	0.42	0.74	0.47	0.82	0.77	0.89
tr:Q93569:Q93569_CAEEL	0.25	0.63	0.37	0.79	0.68	0.86

cert (FBgn0027569) associated phenotypes

heat stress response defective, locomotor behavior defective, short lived, stress response defective

(Information from FlyBase)
