# Supplementary material for: Drug repurposing for aging research using model organisms
Source: Aging Cell. 2017 Jun 16;16(5):1006–15. doi: 10.1111/acel.12626 (PMC5595691; doi:10.1111/acel.12626)
Supplement: Supplementary file 7 — Data S1 Zip‐Archive of all report cards. [file ACEL-16-1006-s007.zip › RC_16W.pdf]

16W

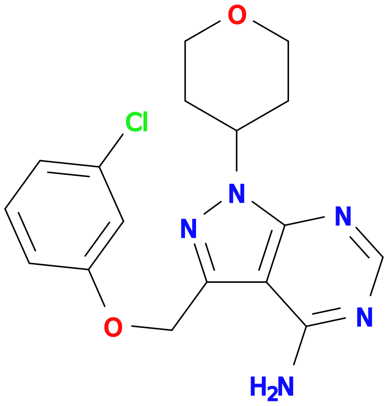

Database identifiers

ChEMBLCompound	CHEMBL2069623

Ranking

	Rank	Score
Drosophila	596/697	0.163
C. elegans	517/591	0.057

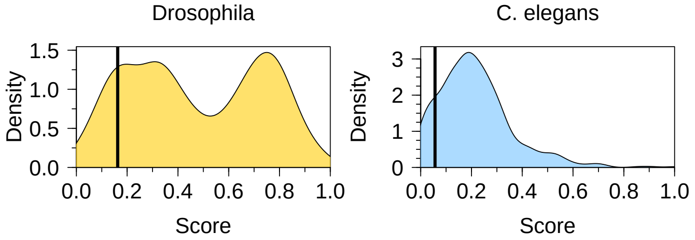

	Ageing implication	Domain conservation	Binding site conservation	Binding affinity	Bioavailability	Lipinski	Promiscuity	Purchasability	Drug approval	**Total**
Drosophila	0.203	0.975	1.0	0.919	(0.9)	0.0	-0.0	0.0	0.0	0.163
C. elegans	0.203	0.969	1.0	0.919	0.316	0.0	-0.0	0.0	0.0	0.057

Names

No synonyms found

Roles

ChEBI entry None has no roles

Status

Approved drug (according to ChEMBL)	No
Number of Rule of 5 violations	0
Binding affinity to original target in log units (RF-Score prediction)	7.42
Burns *C. elegans* bioavailability prediction	0.02

Compound Target Characteristics

Casein kinase I isoform epsilon

Best gene implication in ageing for this target family came from gene P49674 via mapping the annotation from Ensembl ENSG00000213923 via mapping the annotation from EntrezGene 1454 via mapping the annotation from GenAgeHuman 0244 annotated in GenAge release 17.

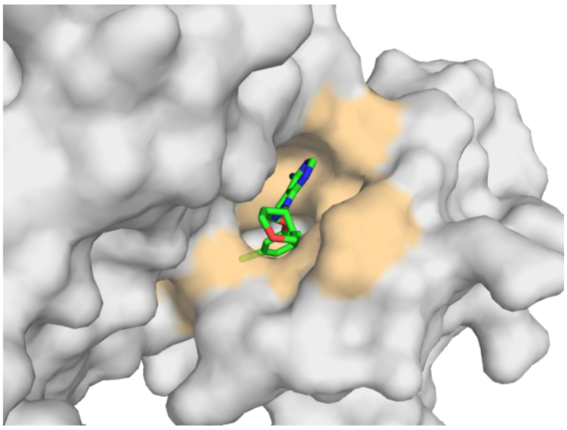

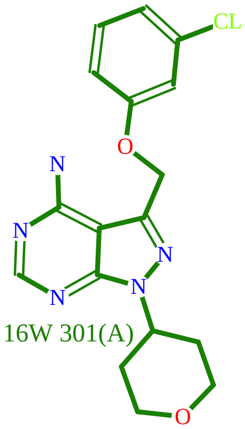

protein	amino acids contacts (binding site)											
PDB:4hni:chainA:P49674	I	I	A	K	M	M	E	L	L	L	D	F
tr:B0QY36:B0QY36_HUMAN	I	I	A	K	M	M	E	L	L	L	D	F
tr:B0QY35:B0QY35_HUMAN	I	I	A	K	M	M	E	L	L	L	D	F
tr:B0QY34:B0QY34_HUMAN	I	I	A	K	M	M	E	L	L	L	D	F
tr:Q5U045:Q5U045_HUMAN	I	I	A	K	M	M	E	L	L	L	D	F
sp:P49674:KC1E_HUMAN	I	I	A	K	M	M	E	L	L	L	D	F
tr:Q9JJ76:Q9JJ76_RAT	I	I	A	K	M	M	E	L	L	L	D	F
tr:Q3TYE1:Q3TYE1_MOUSE	I	I	A	K	M	M	E	L	L	L	D	F
sp:Q9JMK2:KC1E_MOUSE	I	I	A	K	M	M	E	L	L	L	D	F
sp:O76324:DCO_DROME	I	I	A	K	M	M	E	L	L	L	D	F
tr:H9G2V4:H9G2V4_CAEEL	I	I	A	K	M	M	E	L	L	L	D	F
sp:Q20471:KIN20_CAEEL	I	I	A	K	M	M	E	L	L	L	D	F

	whole protein		domain-based		contact-based	
protein	ident	simil	ident	simil	ident	simil
PDB:4hni:chainA:P49674	1.0	1.0	1.0	1.0	1.0	1.0
tr:B0QY36:B0QY36_HUMAN	0.54	0.54	0.83	0.83	1.0	1.0
tr:B0QY35:B0QY35_HUMAN	0.71	0.73	1.0	1.0	1.0	1.0
tr:B0QY34:B0QY34_HUMAN	0.71	0.74	1.0	1.0	1.0	1.0
tr:Q5U045:Q5U045_HUMAN	1.0	1.0	1.0	1.0	1.0	1.0
sp:P49674:KC1E_HUMAN	1.0	1.0	1.0	1.0	1.0	1.0
tr:Q9JJ76:Q9JJ76_RAT	0.99	1.0	1.0	1.0	1.0	1.0
tr:Q3TYE1:Q3TYE1_MOUSE	0.73	0.8	1.0	1.0	1.0	1.0
sp:Q9JMK2:KC1E_MOUSE	0.99	1.0	1.0	1.0	1.0	1.0
sp:O76324:DCO_DROME	0.58	0.73	0.88	0.97	1.0	1.0
tr:H9G2V4:H9G2V4_CAEEL	0.56	0.67	0.8	0.94	1.0	1.0
sp:Q20471:KIN20_CAEEL	0.39	0.47	0.8	0.94	1.0	1.0

dco (FBgn0002413) associated phenotypes

cell autonomous, cell death defective, cell polarity defective, cell size defective, circadian rhythm defective, developmental rate defective, dominant, eclosion rhythm defective, flight defective, hyperplasia, increased cell number, lethal - all die before end of pupal stage, partially lethal, partially lethal - majority die, partially lethal - majority live, polyphasic, size defective, somatic clone, some die during pupal stage, temperature conditional

(Information from FlyBase)

dco (UniProt:O76324) annotation
Function: Involved in circadian rhythms, viability and molecular oscillations of the clock genes period (per) and timeless (tim). Dbt reduces the stability and thus the accumulation of monomeric per proteins, probably through phosphorylation. No evident circadian oscillation is detected in head. (PubMed:10556065, PubMed:9674430, PubMed:9674431).
Subunit: Forms a complex with per.
Tissue specificity: Expressed in photoreceptor cells of the eyes as well as in the region situated between the optic lobe and the central brain. (PubMed:10556065).

(Information from UniProt)

kin-20 (UniProt:Q20471) annotation
Function: Casein kinases are operationally defined by their preferential utilization of acidic proteins such as caseins as substrates. It can phosphorylate a large number of proteins. Participates in Wnt signaling (By similarity). Is a developmental timer that specifies temporal cell fate selection; acts to control the temporal identity of hypodermal seam cells. Required during late-larval development to prevent adult fates, particularly cell cycle exit and fusion, from being expressed too early. (, PubMed:15691769).
Enzyme regulation: Exhibits substrate-dependent heparinactivation.
Subunit: Monomer.
Subcellular location: Cytoplasm
Tissue specificity: Expressed throughout larval development and into the adult stage in both hypodermal seam cells and the hermaphrodite specific neuron. (PubMed:15691769).

(Information from UniProt)
