# Supplementary material for: Drug repurposing for aging research using model organisms
Source: Aging Cell. 2017 Jun 16;16(5):1006–15. doi: 10.1111/acel.12626 (PMC5595691; doi:10.1111/acel.12626)
Supplement: Supplementary file 7 — Data S1 Zip‐Archive of all report cards. [file ACEL-16-1006-s007.zip › RC_1BQ.pdf]

1BQ

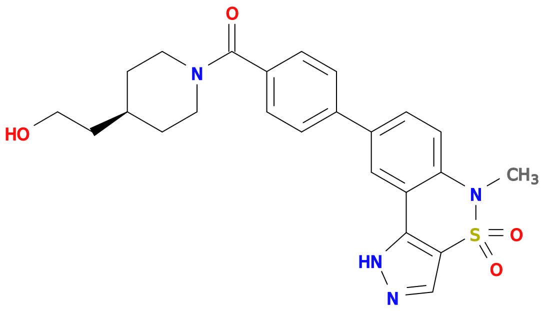

Database identifiers

ChEMBLCompound	CHEMBL2333434

Ranking

	Rank	Score
Drosophila	661/697	0.1
C. elegans	551/591	0.029

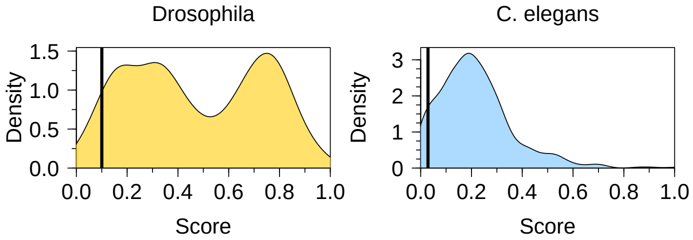

	Ageing implication	Domain conservation	Binding site conservation	Binding affinity	Bioavailability	Lipinski	Promiscuity	Purchasability	Drug approval	**Total**
Drosophila	0.203	0.868	0.725	0.879	(0.9)	0.0	-0.0	0.0	0.0	0.1
C. elegans	0.203	0.844	0.709	0.879	0.275	0.0	-0.0	0.0	0.0	0.029

Names

No synonyms found

Roles

ChEBI entry None has no roles

Status

Approved drug (according to ChEMBL)	No
Number of Rule of 5 violations	0
Binding affinity to original target in log units (RF-Score prediction)	6.98
Burns *C. elegans* bioavailability prediction	-1.09

Compound Target Characteristics

Focal adhesion kinase 1

Best gene implication in ageing for this target family came from gene Q05397 via mapping the annotation from Ensembl ENSG00000169398 via mapping the annotation from EntrezGene 5747 via mapping the annotation from GenAgeHuman 0166 annotated in GenAge release 17.

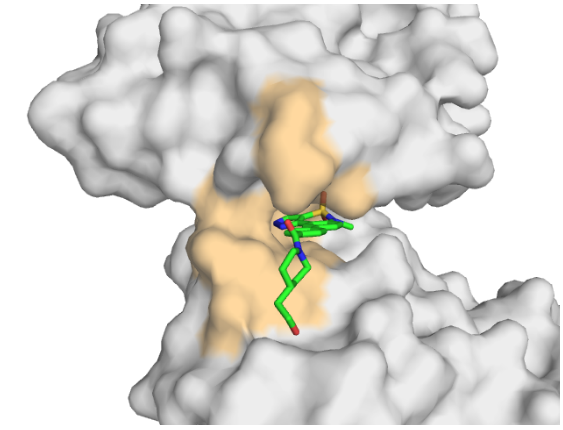

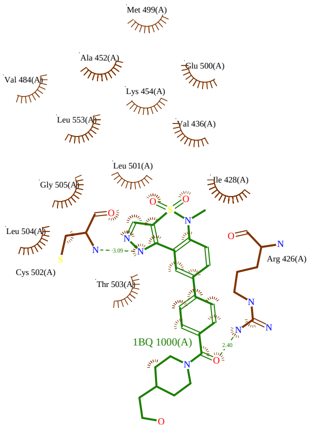

protein	amino acids contacts (binding site)													
PDB:4i4e:chainA:Q05397	R	I	V	A	K	V	M	E	L	C	T	L	G	L
tr:E9PEI4:E9PEI4_HUMAN	R	I	V	A	K	V	M	E	L	C	T	L	G	L
tr:H0YB16:H0YB16_HUMAN	R	I	V	A	K	V	M	E	L	C	T	L	G	L
tr:Q8N9D7:Q8N9D7_HUMAN	R	I	V	A	K	V	M	E	L	C	T	L	G	L
tr:B4DH13:B4DH13_HUMAN	R	I	V	A	K	V	M	E	L	C	T	L	G	L
tr:H0YBZ1:H0YBZ1_HUMAN	R	I	V	A	K	V	M	E	L	C	T	L	G	L
tr:Q8IYN9:Q8IYN9_HUMAN	R	I	V	A	K	V	M	E	L	C	T	L	G	L
sp:Q05397:FAK1_HUMAN	R	I	V	A	K	V	M	E	L	C	T	L	G	L
tr:J3QT16:J3QT16_HUMAN	R	I	V	A	K	V	M	E	L	C	T	L	G	L
tr:E7ESA6:E7ESA6_HUMAN	R	I	V	A	K	V	M	E	L	C	T	L	G	L
tr:R9PXT6:R9PXT6_RAT	R	I	V	A	K	V	M	E	L	C	T	L	G	L
sp:P34152:FAK1_MOUSE	R	I	V	A	K	V	M	E	L	C	T	L	G	L
tr:Q2TGI0:Q2TGI0_DROME	A	I	V	A	K	I	M	E	L	A	K	L	G	L
tr:Q9U5Y2:Q9U5Y2_DROME	A	I	V	A	K	I	M	E	L	A	K	L	G	L
tr:E1JGM8:E1JGM8_DROME	A	I	V	A	K	I	M	E	L	A	K	L	G	L
tr:B8A418:B8A418_DROME	A	I	V	A	K	I	M	E	L	A	K	L	G	L
tr:Q5BIG9:Q5BIG9_DROME	A	I	V	A	K	I	M	E	L	A	K	L	G	L
tr:Q0E917:Q0E917_DROME	A	I	V	A	K	I	M	E	L	A	K	L	G	L
tr:Q9U531:Q9U531_DROME	A	I	V	A	K	I	M	E	L	A	K	L	G	L
tr:Q9U472:Q9U472_DROME	A	I	V	A	K	I	M	E	L	A	K	L	G	L
tr:B7YZL9:B7YZL9_DROME	A	I	V	A	K	I	M	E	L	A	K	L	G	L
tr:Q8T879:Q8T879_CAEEL	E	I	V	A	K	I	L	E	L	A	P	K	G	L
tr:Q95YD4:Q95YD4_CAEEL	E	I	V	A	K	I	L	E	L	A	P	K	G	L

protein	whole protein ident	simil	domain-based ident	simil	contact-based ident	simil
PDB:4i4e:chainA:Q05397	1.0	1.0	1.0	1.0	1.0	1.0
tr:E9PEI4:E9PEI4_HUMAN	0.63	0.64	1.0	1.0	1.0	1.0
tr:H0YB16:H0YB16_HUMAN	0.68	0.68	0.97	0.97	1.0	1.0
tr:Q8N9D7:Q8N9D7_HUMAN	0.67	0.67	0.97	0.97	1.0	1.0
tr:B4DH13:B4DH13_HUMAN	0.65	0.65	0.97	0.97	1.0	1.0
tr:H0YBZ1:H0YBZ1_HUMAN	0.66	0.66	0.97	0.97	1.0	1.0
tr:Q8IYN9:Q8IYN9_HUMAN	0.96	0.96	1.0	1.0	1.0	1.0
sp:Q05397:FAK1_HUMAN	1.0	1.0	1.0	1.0	1.0	1.0
tr:J3QT16:J3QT16_HUMAN	0.99	0.99	1.0	1.0	1.0	1.0
tr:E7ESA6:E7ESA6_HUMAN	0.96	0.96	1.0	1.0	1.0	1.0
tr:R9PXT6:R9PXT6_RAT	0.97	0.99	0.99	1.0	1.0	1.0
sp:P34152:FAK1_MOUSE	0.94	0.96	0.94	0.95	1.0	1.0
tr:Q2TGI0:Q2TGI0_DROME	0.16	0.23	0.56	0.79	0.71	0.72
tr:Q9U5Y2:Q9U5Y2_DROME	0.3	0.62	0.56	0.79	0.71	0.72
tr:E1JGM8:E1JGM8_DROME	0.3	0.62	0.56	0.79	0.71	0.72
tr:B8A418:B8A418_DROME	0.3	0.62	0.56	0.79	0.71	0.72
tr:Q5BIG9:Q5BIG9_DROME	0.3	0.62	0.56	0.79	0.71	0.72
tr:Q0E917:Q0E917_DROME	0.3	0.62	0.56	0.79	0.71	0.72
tr:Q9U531:Q9U531_DROME	0.3	0.62	0.56	0.79	0.71	0.72
tr:Q9U472:Q9U472_DROME	0.3	0.62	0.56	0.79	0.71	0.72
tr:B7YZL9:B7YZL9_DROME	0.25	0.52	0.56	0.79	0.71	0.72
tr:Q8T879:Q8T879_CAEEL	0.21	0.53	0.47	0.77	0.57	0.71
tr:Q95YD4:Q95YD4_CAEEL	0.21	0.55	0.47	0.77	0.57	0.71

Fak (FBgn0020440) associated phenotypes

neuroanatomy defective, neurophysiology defective

(Information from FlyBase)
