# Supplementary material for: Drug repurposing for aging research using model organisms
Source: Aging Cell. 2017 Jun 16;16(5):1006–15. doi: 10.1111/acel.12626 (PMC5595691; doi:10.1111/acel.12626)
Supplement: Supplementary file 7 — Data S1 Zip‐Archive of all report cards. [file ACEL-16-1006-s007.zip › RC_1DA.pdf]

1DA

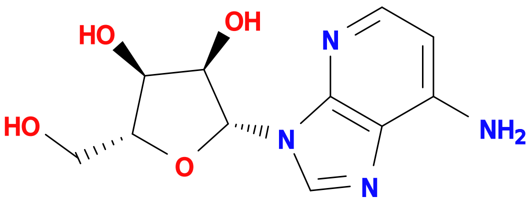

Database identifiers

ChEMBLCompound	CHEMBL115824
ZINC	ZINC03814313

Ranking

	Rank	Score
Drosophila	NA	NA
C. elegans	261/591	0.213

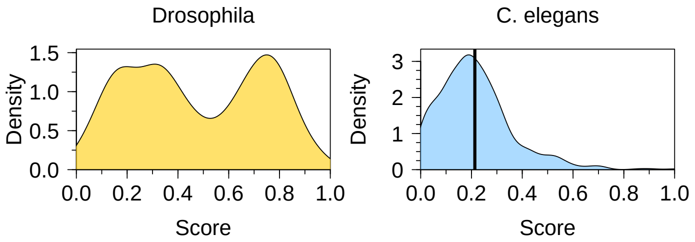

	Ageing implication	Domain conservation	Binding site conservation	Binding affinity	Bioavailability	Lipinski	Promiscuity	Purchasability	Drug approval	**Total**
Drosophila	NA	NA	NA	NA	NA	NA	NA	NA	NA	NA
C. elegans	0.792	0.853	0.965	0.854	0.204	0.0	-0.0	0.1	0.0	0.213

Names

- (2R,3R,4S,5R)-2-(7-amino-3-imidazo5,4-bpyridinyl)-5-(hydroxymethyl)tetrahydrofuran-3,4-diol
- (2R,3R,4S,5R)-2-(7-aminoimidazo5,4-bpyridin-3-yl)-5-(hydroxymethyl)oxolane-3,4-diol
- (2R,3R,4S,5R)-2-(7-aminoimidazo5,4-bpyridin-3-yl)-5-methylol-tetrahydrofuran-3,4-diol
- 1-Deaza-A
- 1-Deazaadenosine
- 3H-Imidazo(4,5-b)pyridin-7-amine, 3-beta-D-ribofuranosyl-

Roles

ChEBI entry None has no roles

Status

Approved drug (according to ChEMBL)	No
Number of Rule of 5 violations	0
Binding affinity to original target in log units (RF-Score prediction)	6.77
Burns *C. elegans* bioavailability prediction	-7.73

Compound Target Characteristics

Adenosine deaminase

Best gene implication in ageing for this target family came from gene Q920P6 annotated in UniProt release 2014_02. Annotation GO 7568 (aging) was Inferred from Expression Pattern

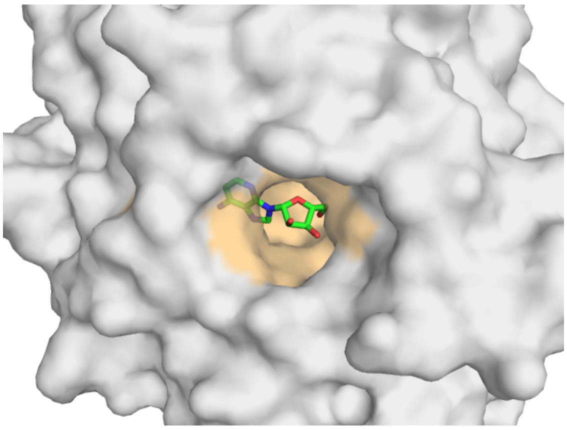

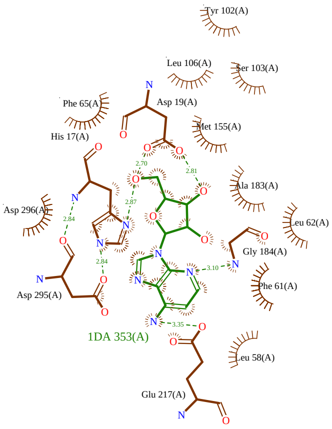

protein	amino acids contacts (binding site)															
PDB:1add:chainA:P03958	H	D	L	F	L	F	R	Y	S	L	M	A	G	E	D	D
sp:P00813:ADA_HUMAN	H	D	L	F	L	F	R	Y	S	L	M	A	G	E	D	D
sp:Q920P6:ADA_RAT	H	D	L	F	L	F	R	Y	S	L	M	A	G	E	D	D
sp:P03958:ADA_MOUSE	H	D	L	F	L	F	R	Y	S	L	M	A	G	E	D	D
tr:Q4FK28:Q4FK28_MOUSE	H	D	L	F	L	F	R	Y	S	L	M	A	G	E	D	D
tr:Q86NI2:Q86NI2_CAEEL	H	D	L	V	L	F	R	Y	S	L	I	A	G	E	D	D
tr:H2KYI5:H2KYI5_CAEEL	H	D	L	V	L	F	R	Y	S	L	I	A	G	E	D	D
sp:P53909:ADE_YEAST	H	E	L	F	L	-	F	Y	D	-	L	D	S	E	D	D

protein	whole protein ident	whole protein simil	domain-based ident	domain-based simil	contact-based ident	contact-based simil
PDB:1add:chainA:P03958	1.0	1.0	1.0	1.0	1.0	1.0
sp:P00813:ADA_HUMAN	0.81	0.93	0.83	0.96	1.0	1.0
sp:Q920P6:ADA_RAT	0.94	0.99	0.94	0.99	1.0	1.0
sp:P03958:ADA_MOUSE	1.0	1.0	1.0	1.0	1.0	1.0
tr:Q4FK28:Q4FK28_MOUSE	1.0	1.0	1.0	1.0	1.0	1.0
tr:Q86NI2:Q86NI2_CAEEL	0.39	0.76	0.4	0.78	0.88	0.97
tr:H2KYI5:H2KYI5_CAEEL	0.35	0.69	0.4	0.78	0.88	0.97
sp:P53909:ADE_YEAST	0.26	0.68	0.27	0.68	0.5	0.52
