# Supplementary material for: Drug repurposing for aging research using model organisms
Source: Aging Cell. 2017 Jun 16;16(5):1006–15. doi: 10.1111/acel.12626 (PMC5595691; doi:10.1111/acel.12626)
Supplement: Supplementary file 7 — Data S1 Zip‐Archive of all report cards. [file ACEL-16-1006-s007.zip › RC_1EJ.pdf]

1EJ

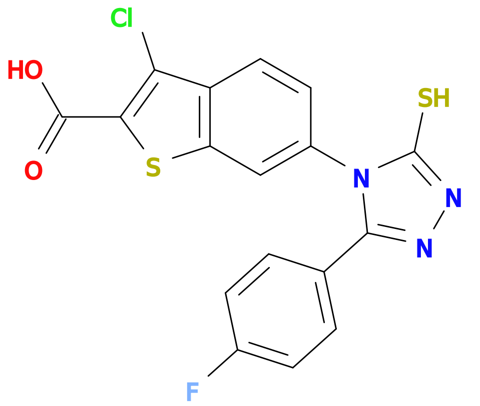

Database identifiers

ChEMBLCompound	CHEMBL2407464
eMolecules	4084605

Ranking

	Rank	Score
Drosophila	582/697	0.172
C. elegans	457/591	0.106

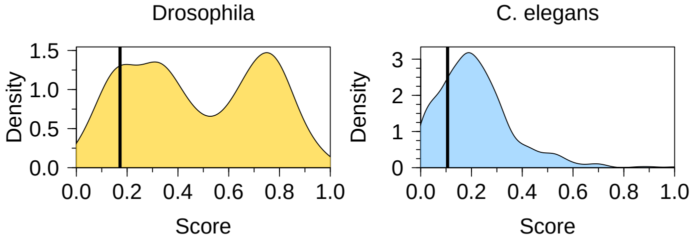

	Ageing implication	Domain conservation	Binding site conservation	Binding affinity	Bioavailability	Lipinski	Promiscuity	Purchasability	Drug approval	**Total**
Drosophila	0.36	0.831	0.613	0.439	(0.9)	0.0	-0.0	0.1	0.0	0.172
C. elegans	0.36	0.397	0.416	0.439	0.246	0.0	-0.0	0.1	0.0	0.106

Names

No synonyms found

Roles

ChEBI entry None has no roles

Status

Approved drug (according to ChEMBL)	No
Number of Rule of 5 violations	0
Binding affinity to original target in log units (RF-Score prediction)	4.75
Burns *C. elegans* bioavailability prediction	-2.3

Compound Target Characteristics

Replication protein A 70 kDa DNA-binding subunit

Best gene implication in ageing for this target family came from gene Q19537 via mapping the annotation from Ensembl WBGene00017546 via mapping the annotation from EntrezGene 174238 via mapping the annotation from GenAgeModels 1779 annotated in GenAge release 17.

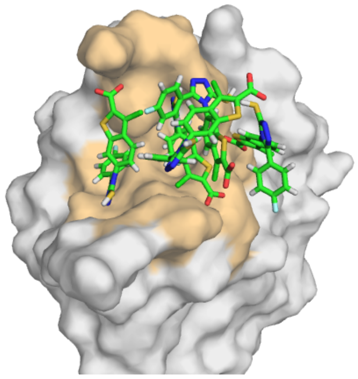

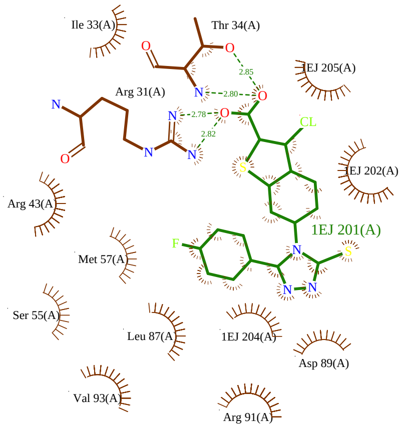

protein	amino acids contacts (binding site)															
PDB:4ijh:chainA:P27694	R	I	T	T	R	R	S	S	F	M	L	K	D	R	V	I
tr:I3L524:I3L524_HUMAN	R	I	T	T	R	R	S	S	F	M	L	K	D	R	V	I
tr:I3L2M5:I3L2M5_HUMAN	R	I	T	T	R	R	S	S	F	M	L	K	D	R	V	I
sp:P27694:RFA1_HUMAN	R	I	T	T	R	R	S	S	F	M	L	K	D	R	V	I
tr:Q7TP21:Q7TP21_RAT	R	I	T	T	R	R	S	S	F	M	L	K	D	R	V	I
sp:Q8VEE4:RFA1_MOUSE	R	I	S	T	R	R	S	S	F	M	L	K	D	R	V	V
tr:Q5SWN2:Q5SWN2_MOUSE	R	I	S	T	R	R	S	S	F	M	L	K	D	R	V	V
sp:Q24492:RFA1_DROME	K	I	N	S	R	R	S	Y	A	M	V	G	K	K	V	I
sp:Q19537:RFA1_CAEEL	E	G	E	Q	T	R	N	A	-	L	Q	G	E	A	N	F
sp:P22336:RFA1_YEAST	R	S	D	G	K	L	K	A	-	L	V	R	E	K	Y	L

	whole protein		domain-based		contact-based	
protein	ident	simil	ident	simil	ident	simil
PDB:4ijh:chainA:P27694	0.97	0.97	0.99	1.0	1.0	1.0
tr:I3L524:I3L524_HUMAN	0.2	0.2	0.89	0.89	1.0	1.0
tr:I3L2M5:I3L2M5_HUMAN	0.25	0.25	0.89	0.89	1.0	1.0
sp:P27694:RFA1_HUMAN	1.0	1.0	1.0	1.0	1.0	1.0
tr:Q7TP21:Q7TP21_RAT	0.76	0.84	0.79	0.86	1.0	1.0
sp:Q8VEE4:RFA1_MOUSE	0.84	0.94	0.8	0.95	0.88	0.95
tr:Q5SWN2:Q5SWN2_MOUSE	0.82	0.91	0.68	0.81	0.88	0.95
sp:Q24492:RFA1_DROME	0.41	0.76	0.35	0.76	0.44	0.61
sp:Q19537:RFA1_CAEEL	0.25	0.68	0.13	0.56	0.06	0.42
sp:P22336:RFA1_YEAST	0.31	0.72	0.19	0.69	0.06	0.49

RpA-70 (FBgn0010173) associated phenotypes

neuroanatomy defective, partially lethal - majority die

(Information from FlyBase)

RpA-70 (UniProt:Q24492) annotation

Function: As part of the heterotrimeric replication protein A complex (RPA/RP-A), binds and stabilizes single-stranded DNA intermediates, that form during DNA replication or upon DNA stress. It prevents their reannealing and in parallel, recruits and activates different proteins and complexes involved in DNA metabolism. Thereby, it plays an essential role both in DNA replication and the cellular response to DNA damage. (UniProtKB:P27694).
Subunit: Component of the heterotrimeric canonical replication protein A complex (RPA).
Subcellular location: Nucleus.

(Information from UniProt)

rpa-1 (UniProt:Q19537) annotation

Function: As part of the heterotrimeric replication protein A complex (RPA/RP-A), binds and stabilizes single-stranded DNA intermediates, that form during DNA replication or upon DNA stress. It prevents their reannealing and in parallel, recruits and activates different proteins and complexes involved in DNA metabolism. Thereby, it plays an essential role both in DNA replication and the cellular response to DNA damage. (UniProtKB:P27694).

Subunit: Component of the heterotrimeric canonical replication protein A complex (RPA).

Subcellular location: Nucleus ECO:0000250.

(Information from UniProt)
