# Supplementary material for: Drug repurposing for aging research using model organisms
Source: Aging Cell. 2017 Jun 16;16(5):1006–15. doi: 10.1111/acel.12626 (PMC5595691; doi:10.1111/acel.12626)
Supplement: Supplementary file 7 — Data S1 Zip‐Archive of all report cards. [file ACEL-16-1006-s007.zip › RC_1N1.pdf]

1N1

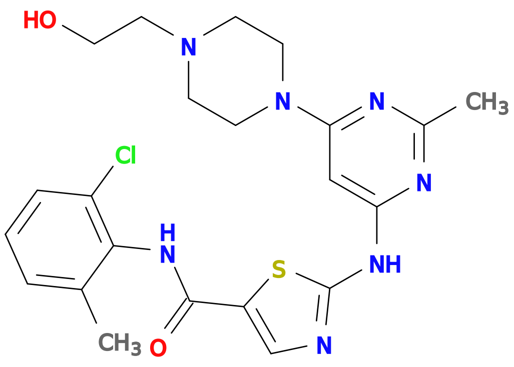

Database identifiers

ChEMBLCompound	CHEMBL1421
DrugBank	DB01254
CHEBI	49375
ZINC	ZINC21982951
eMolecules	10748986

Ranking

	Rank	Score
Drosophila	1/697	1.0
C. elegans	59/591	0.405

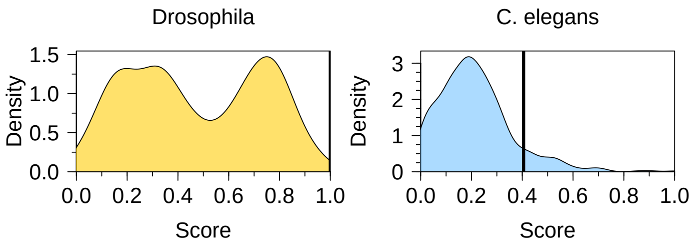

	Ageing implication	Domain conservation	Binding site conservation	Binding affinity	Bioavailability	Lipinski	Promiscuity	Purchasability	Drug approval	**Total**
Drosophila	1.0	0.959	1.0	0.955	(0.9)	0.0	-0.004	0.1	0.1	1.0
C. elegans	1.0	0.922	0.818	0.955	0.29	0.0	-0.004	0.1	0.1	0.405

Names

- **dasatinib (anhydrous)**
- anh. dasatinib
- anhydrous dasatinib
- Bms-354825
- Dasatinib
- dasatinib (anh.)
- Sprycel

Roles

tyrosine kinase inhibitor, antineoplastic agent, Tyrosine-protein kinase ABL inhibitor, Platelet-derived growth factor receptor beta inhibitor, Stem cell growth factor receptor inhibitor, SRC inhibitor, Tyrosine-protein kinase LCK inhibitor, Ephrin type-A receptor 2 inhibitor

Status

Approved drug (according to ChEMBL)	Yes
Administration Route	Oral
Number of Rule of 5 violations	0
Binding affinity to original target in log units (RF-Score prediction)	8.04
Burns *C. elegans* bioavailability prediction	-0.64

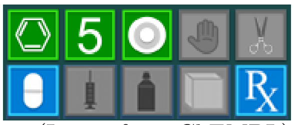

(Image from ChEMBL)

Medical Information

Indicatation: For the treatment of adults with chronic, accelerated, or myeloid or lymphoid blast phase chronic myeloid leukemia with resistance or intolerance to prior therapy. Also indicated for the treatment of adults with Philadelphia chromosome-positive acute lymphoblastic leukemia with resistance or intolerance to prior therapy.
Mechanism of action: Dasatinib, at nanomolar concentrations, inhibits the following kinases: BCR-ABL, SRC family (SRC, LCK, YES, FYN), c-KIT, EPHA2, and PDGFRamp;beta;. Based on modeling studies, dasatinib is predicted to bind to multiple conformations of the ABL kinase. In vitro, dasatinib was active in leukemic cell lines representing variants of imatinib mesylate sensitive and resistant disease. Dasatinib inhibited the growth of chronic myeloid leukemia (CML) and acute lymphoblastic leukemia (ALL) cell lines overexpressing BCR-ABL. Under the conditions of the assays, dasatinib was able to overcome imatinib resistance resulting from BCR-ABL kinase domain mutations, activation of alternate signaling pathways involving the SRC family kinases (LYN, HCK), and multi-drug resistance gene overexpression.
Toxicity: Acute overdose in animals was associated with cardiotoxicity.
Metabolism: Dasatinib is extensively metabolized in humans, primarily by the cytochrome P450 enzyme 3A4
Retail: Bristol-Myers Squibb Co., E.R. Squibb and Sons LLC, Physicians Total Care Inc.
Sprycel 20 mg tablet - 69.56 USD / tablet
Sprycel 50 mg tablet - 139.12 USD / tablet
Sprycel 70 mg tablet - 139.12 USD / tablet
Sprycel 100 mg tablet - 278.24 USD / tablet

(Information from DrugBank)

Compound Target Characteristics

Cytoplasmic tyrosine-protein kinase BMX

Best gene implication in ageing for this target family came from gene P08630 annotated in UniProt release 2014_02. Annotation GO 8340 (determination of adult lifespan) was Inferred from Mutant Phenotype

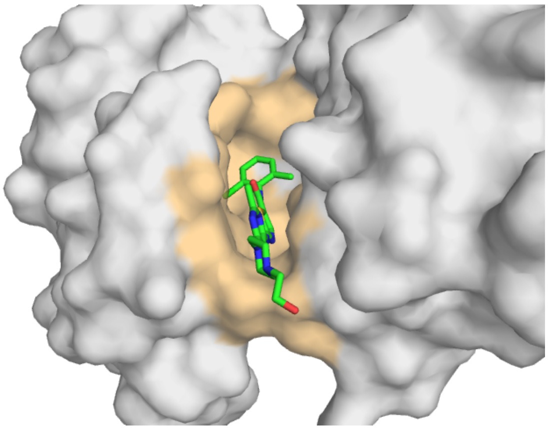

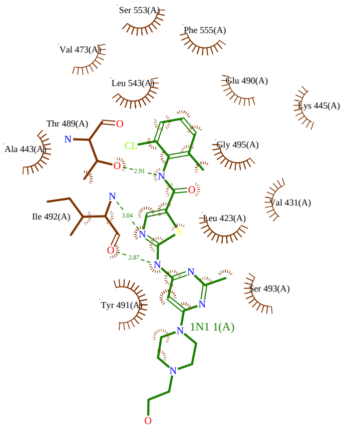

protein	amino acids contacts (binding site)														
PDB:3sxr:chainA:P51813	L	V	A	K	V	I	T	E	Y	I	S	G	L	S	F
sp:P51813:BMX_HUMAN	L	V	A	K	V	I	T	E	Y	I	S	G	L	S	F
tr:F1LVI0:F1LVI0_RAT	L	V	A	K	V	I	T	E	Y	I	T	G	L	S	F
tr:B1AUL6:B1AUL6_MOUSE	L	V	A	K	V	I	T	E	Y	I	T	G	L	S	F
tr:B1AUL5:B1AUL5_MOUSE	L	V	A	K	V	I	T	E	Y	I	T	G	L	S	F
sp:P97504:BMX_MOUSE	L	V	A	K	V	I	T	E	Y	I	T	G	L	S	F
sp:P08630:BTKL_DROME	L	V	A	K	V	I	T	E	Y	M	K	G	L	A	F

	whole protein		domain-based		contact-based	
protein	ident	simil	ident	simil	ident	simil
PDB:3sxr:chainA:P51813	0.98	0.99	0.98	0.99	1.0	1.0
sp:P51813:BMX_HUMAN	1.0	1.0	1.0	1.0	1.0	1.0
tr:F1LVI0:F1LVI0_RAT	0.85	0.92	0.95	0.99	0.93	0.96
tr:B1AUL6:B1AUL6_MOUSE	0.87	0.93	0.95	0.98	0.93	0.96
tr:B1AUL5:B1AUL5_MOUSE	0.88	0.94	0.95	0.98	0.93	0.96
sp:P97504:BMX_MOUSE	0.88	0.94	0.95	0.98	0.93	0.96
sp:P08630:BTKL_DROME	0.31	0.59	0.59	0.86	0.8	0.85

Btk29A (FBgn0003502) associated phenotypes

cell shape defective, lethal - all die before end of first instar larval stage, mating defective, partially, short lived, size defective, some die during first instar larval stage

(Information from FlyBase)

Btk29A (UniProt:P08630) annotation

Function: Required for proper ring canal development. Also required for the development of male genitalia and for adult survival. (PubMed:10330180, PubMed:9655810).
Cofactor: Zn(2+)Note=Binds 1 zinc ion per subunit. ;
Subcellular location: Note=Ring canals.
Tissue specificity: Ring canals in the egg chambers and imaginal disks of third-instar larvae. (PubMed:10330180, PubMed:3110602, PubMed:9655810).
Developmental stage: Expressed both maternally and zygotically. Predominantly in early to middle embryogenesis, in larvae and adult females. (PubMed:10330180, PubMed:3110602).
Disruption phenotype: Flies exhibit shortened copulatory duration (due to incomplete fusion of the left and right halves of the apodeme that holds the penis during copulation) and reduced adult-stage life span. (PubMed:10330180).

(Information from UniProt)

Tyrosine-protein kinase BTK

Best gene implication in ageing for this target family came from gene P08630 annotated in UniProt release 2014_02. Annotation GO 8340 (determination of adult lifespan) was Inferred from Mutant Phenotype

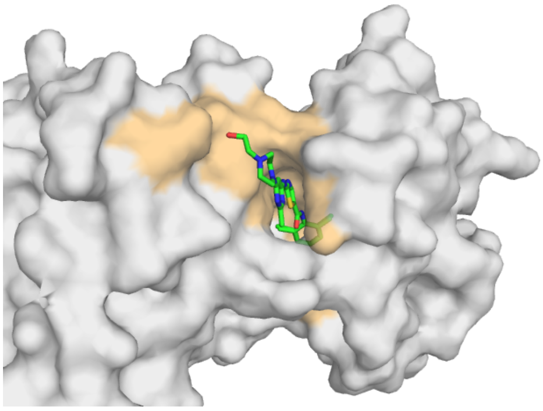

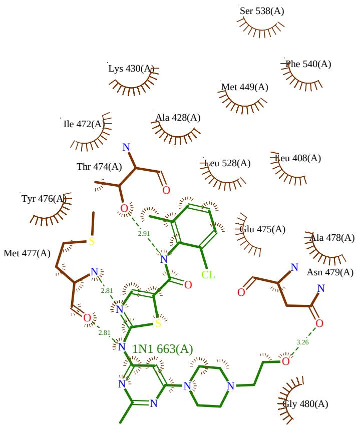

protein	amino acids contacts (binding site)																	
PDB:3k54:chainA:Q06187	L	A	K	E	M	V	I	T	E	Y	M	A	N	G	E	L	S	F
PDB:3oct:chainA:Q06187	L	A	K	E	M	V	I	T	E	Y	M	A	N	G	E	L	S	F
tr:Q3MS96:Q3MS96_HUMAN	L	A	K	E	M	V	I	T	E	Y	M	A	N	G	E	L	S	F
sp:Q06187:BTK_HUMAN	L	A	K	E	M	V	I	T	E	Y	M	A	N	G	E	L	S	F
tr:Q501W1:Q501W1_RAT	I	A	K	E	M	V	I	T	E	F	M	E	N	G	E	L	S	F
tr:F1LVI0:F1LVI0_RAT	L	A	K	E	M	V	I	T	E	Y	I	T	N	G	N	L	S	F
tr:E9PTF7:E9PTF7_RAT	L	A	K	E	M	V	I	T	E	Y	M	A	N	G	E	Q	S	E
sp:P35991:BTK_MOUSE	L	A	K	E	M	V	I	T	E	Y	M	A	N	G	E	L	S	F
sp:P08630:BTKL_DROME	L	A	K	E	M	V	I	T	E	Y	M	K	H	G	R	L	A	F

	whole protein		domain-based		contact-based	
protein	ident	simil	ident	simil	ident	simil
PDB:3k54:chainA:Q06187	1.0	1.0	1.0	1.0	1.0	1.0
PDB:3oct:chainA:Q06187	1.0	1.0	1.0	1.0	1.0	1.0
tr:Q3MS96:Q3MS96_HUMAN	0.48	0.48	0.96	0.96	1.0	1.0
sp:Q06187:BTK_HUMAN	1.0	1.0	1.0	1.0	1.0	1.0
tr:Q501W1:Q501W1_RAT	0.4	0.65	0.62	0.88	0.83	0.93
tr:F1LVI0:F1LVI0_RAT	0.48	0.77	0.66	0.91	0.83	0.94
tr:E9PTF7:E9PTF7_RAT	0.93	0.97	0.92	0.97	0.89	0.87
sp:P35991:BTK_MOUSE	0.98	1.0	0.99	1.0	1.0	1.0
sp:P08630:BTKL_DROME	0.36	0.63	0.58	0.87	0.78	0.83

Btk29A (FBgn0003502) associated phenotypes

cell shape defective, lethal - all die before end of first instar larval stage, mating defective, partially, short lived, size defective, some die during first instar larval stage

(Information from FlyBase)

Btk29A (UniProt:P08630) annotation

Function: Required for proper ring canal development. Also required for the development of male genitalia and for adult survival. (PubMed:10330180, PubMed:9655810).
Cofactor: Zn(2+)Note=Binds 1 zinc ion per subunit. ;
Subcellular location: Note=Ring canals.
Tissue specificity: Ring canals in the egg chambers and imaginal disks of third-instar larvae. (PubMed:10330180, PubMed:3110602, PubMed:9655810).
Developmental stage: Expressed both maternally and zygotically. Predominantly in early to middle embryogenesis, in larvae and adult females. (PubMed:10330180, PubMed:3110602).
Disruption phenotype: Flies exhibit shortened copulatory duration (due to incomplete fusion of the left and right halves of the apodeme that holds the penis during copulation) and reduced adult-stage life span. (PubMed:10330180).

(Information from UniProt)

Mitogen-activated protein kinase 14

Best gene implication in ageing for this target family came from gene O62618 annotated in UniProt release 2014_02. Annotation GO 8340 (determination of adult lifespan) was Inferred from Mutant Phenotype

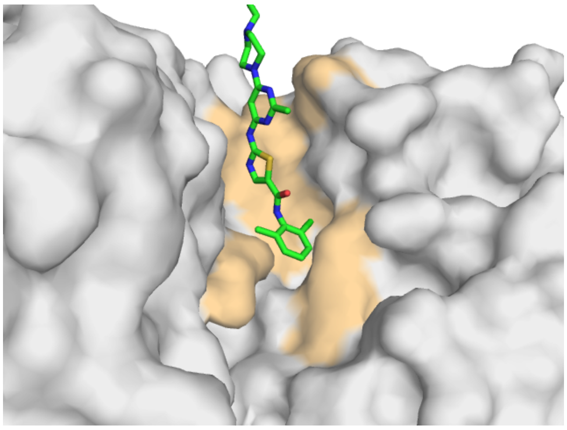

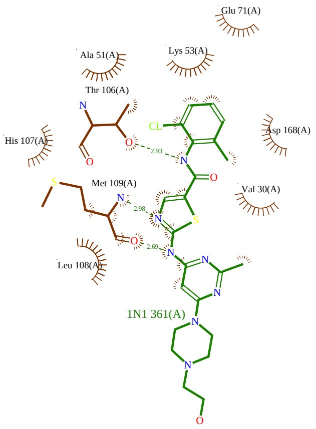

protein	amino acids contacts (binding site)
PDB:3lfa:chainA:Q16539	V A K E L T H L M D
tr:L7RSM2:L7RSM2_HUMAN	V A K E L T H L M D
sp:Q16539:MK14_HUMAN	V A K E L T H L M D
tr:G3V617:G3V617_RAT	V A K E L T H L M D
tr:Q56A33:Q56A33_RAT	V A K E L T H L M D
tr:B2KF34:B2KF34_MOUSE	V A K E L T H L M D
tr:B2KF35:B2KF35_MOUSE	V A K E L T H L M D
sp:P47811:MK14_MOUSE	V A K E L T H L M D
tr:Q5U421:Q5U421_MOUSE	V A K E L T H L M D
sp:O62618:MK14A_DROME	V A K E L T H L M D
tr:E1JIV6:E1JIV6_DROME	V A K E L T H L M D
sp:Q17446:PMK1_CAEEL	I A K E F S M L M D
sp:Q8MXI4:PMK2_CAEEL	L A K E F S V L M D
sp:P32485:HOG1_YEAST	V A K E F T E L Q D

	whole protein		domain-based		contact-based	
protein	ident	simil	ident	simil	ident	simil
PDB:3lfa:chainA:Q16539	1.0	1.0	1.0	1.0	1.0	1.0
tr:L7RSM2:L7RSM2_HUMAN	0.96	0.99	0.95	0.99	1.0	1.0
sp:Q16539:MK14_HUMAN	1.0	1.0	1.0	1.0	1.0	1.0
tr:G3V617:G3V617_RAT	0.99	1.0	0.99	1.0	1.0	1.0
tr:Q56A33:Q56A33_RAT	0.95	0.99	0.95	0.99	1.0	1.0
tr:B2KF34:B2KF34_MOUSE	0.56	0.56	0.62	0.62	1.0	1.0
tr:B2KF35:B2KF35_MOUSE	0.7	0.71	0.8	0.8	1.0	1.0
sp:P47811:MK14_MOUSE	0.99	1.0	0.99	1.0	1.0	1.0
tr:Q5U421:Q5U421_MOUSE	0.96	0.99	0.95	0.99	1.0	1.0
sp:O62618:MK14A_DROME	0.67	0.88	0.74	0.91	1.0	1.0
tr:E1JIV6:E1JIV6_DROME	0.67	0.88	0.74	0.91	1.0	1.0
sp:Q17446:PMK1_CAEEL	0.62	0.85	0.71	0.92	0.6	0.82
sp:Q8MXI4:PMK2_CAEEL	0.49	0.74	0.59	0.84	0.6	0.82
sp:P32485:HOG1_YEAST	0.41	0.68	0.54	0.85	0.7	0.85

Mpk2 (FBgn0015765) associated phenotypes

heat stress response defective, immune response defective, non-enhancer of variegation, non-suppressor of variegation, nutrition conditional, osmotic stress response defective, oxidative stress response defective, partially lethal - majority die, reduced, short lived, starvation stress response defective, stress response defective

(Information from FlyBase)

Mpk2 (UniProt:O62618) annotation
Function: Kinase involved in a signal transduction pathway. May down-regulate insect immunity gene expression after prolonged infection. (PubMed:9417090, PubMed:9584193).
Cofactor:Mg(2+)
Enzyme regulation: Activated by threonine and tyrosine phosphorylation by Mkk3 in response to environmental stress. (PubMed:9417090, PubMed:9584193).
Subcellular location: Nucleus (PubMed:9417090).
Developmental stage: Expressed both maternally and zygotically. Levels are highest at the preblastoderm stage but low levels are present throughout development. (PubMed:9584193).
Domain: The TXY motif contains the threonine and tyrosine residues whose phosphorylation activates the MAP kinases.
Ptm: Dually phosphorylated on Thr-184 and Tyr-186, which activates the enzyme. (PubMed:9417090).
(Information from UniProt)

pmk-1 (WBGene00004055) associated phenotypes
cell stress response variant, drug induced gene expression variant, gene expression level reduced, lethal, life span variant, organism oxidative stress response hypersensitive, pathogen induced gene expression variant, pathogen susceptibility increased, sterile
(Information from WormBase)

pmk-1 (UniProt:Q17446) annotation
Function: Responds to activation by environmental stress and pro- inflammatory cytokines by phosphorylating downstream targets. Functions downstream of the MAPKK sek-1 and the MAPKKK nsy-1 as the MAP kinase required for pathogen resistance. (PubMed:11703092, PubMed:12142542).
Cofactor: Mg(2+) Evidence=(PubMed:11703092);
Enzyme regulation: Activated by phosphorylation on threonine and tyrosine. Inhibited by pyridinyl-imidazole related compounds. (PubMed:11703092).
Domain: The TXY motif contains the threonine and tyrosine residues whose phosphorylation activates the MAP kinases.
Ptm: Dually phosphorylated on Thr-191 and Tyr-193, which activates the enzyme.
(Information from UniProt)

pmk-2 (UniProt:Q8MXI4) annotation
Function: Responds to activation by environmental stress and pro- inflammatory cytokines by phosphorylating downstream targets. (PubMed:11703092).
Cofactor: Mg(2+) Evidence=(PubMed:11703092);
Enzyme regulation: Activated by phosphorylation on threonine and tyrosine. Inhibited by pyridinyl-imidazole related compounds. (PubMed:11703092).
Subcellular location: Cytoplasm (PubMed:11703092).
Domain: The TXY motif contains the threonine and tyrosine residues whose phosphorylation activates the MAP kinases.
Ptm: Dually phosphorylated on Thr-222 and Tyr-224, which activates the enzyme.
(Information from UniProt)
