# Supplementary material for: Drug repurposing for aging research using model organisms
Source: Aging Cell. 2017 Jun 16;16(5):1006–15. doi: 10.1111/acel.12626 (PMC5595691; doi:10.1111/acel.12626)
Supplement: Supplementary file 7 — Data S1 Zip‐Archive of all report cards. [file ACEL-16-1006-s007.zip › RC_1PE.pdf]

# 1PE

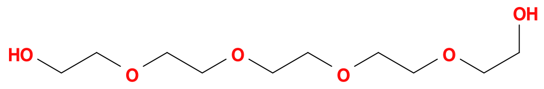


**Database identifiers**

| | |
|---|---|
| ChEMBLCompound | CHEMBL1229766 |
| CHEBI | 39631 |
| ZINC | ZINC04283769 |
| eMolecules | 498770 |

## Ranking

| | Rank | Score |
|---|---|---|
| Drosophila | 490/697 | 0.288 |
| C. elegans | 444/591 | 0.114 |

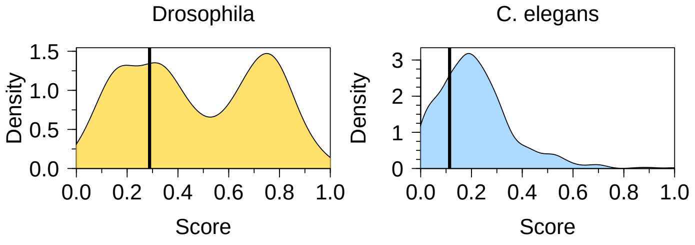


| | Ageing implication | Domain conservation | Binding site conservation | Binding affinity | Bioavailability | Lipinski | Promiscuity | Purchasability | Drug approval | **Total** |
|---|---|---|---|---|---|---|---|---|---|---|
| Drosophila | 1.0 | 0.958 | 0.91 | 0.309 | (0.9) | 0.0 | -0.054 | 0.1 | 0.0 | 0.288 |
| C. elegans | 1.0 | 0.948 | 1.0 | 0.309 | 0.231 | 0.0 | -0.054 | 0.1 | 0.0 | 0.114 |

## Names

· **pentaethylene glycol**
· Hoch2ch2o5h

## Roles

ChEBI entry 39631 has no roles

## Status

| | |
|---|---|
| Approved drug (according to ChEMBL) | No |
| Number of Rule of 5 violations | 0 |
| Binding affinity to original target in log units (RF-Score prediction) | 4.19 |
| Burns *C. elegans* bioavailability prediction | -3.21 |

## Compound Target Characteristics

### Menin

Best gene implication in ageing for this target family came from gene Q8SXU2 annotated in UniProt release 2014_02. Annotation GO 8340 (determination of adult lifespan) was Inferred from Mutant Phenotype

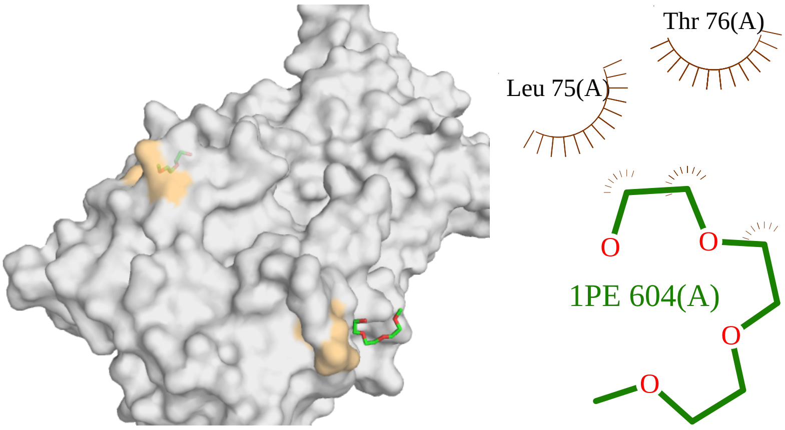


| protein | amino acids contacts (binding site) |
|---|---|
| PDB:4gq6:chainA:O00255 | L T W S R K W |
| tr:E7ENS2:E7ENS2_HUMAN | L T W S R K W |
| tr:E7EPR4:E7EPR4_HUMAN | L T W S R K W |
| tr:E7ET29:E7ET29_HUMAN | L T W S R K W |
| tr:E7EN32:E7EN32_HUMAN | L T W S R K W |
| sp:O00255:MEN1_HUMAN | L T W S R K W |
| sp:Q9WVR8:MEN1_RAT | L T W S R K W |
| sp:O88559:MEN1_MOUSE | L T W S R K W |
| tr:F8WHD9:F8WHD9_MOUSE | L T W S R K W |
| tr:Q8SXU2:Q8SXU2_DROME | I S W I R K W |
| tr:Q7KXY9:Q7KXY9_DROME | I S W I R K W |
| tr:Q9VM47:Q9VM47_DROME | I S W I R K W |

| | whole protein | | domain-based | | contact-based | |
|---|---|---|---|---|---|---|
| protein | ident | simil | ident | simil | ident | simil |
| PDB:4gq6:chainA:O00255 | 1.0 | 1.0 | 1.0 | 1.0 | 1.0 | 1.0 |
| tr:E7ENS2:E7ENS2_HUMAN | 0.42 | 0.42 | 0.43 | 0.43 | 1.0 | 1.0 |
| tr:E7EPR4:E7EPR4_HUMAN | 0.46 | 0.46 | 0.46 | 0.46 | 1.0 | 1.0 |
| tr:E7ET29:E7ET29_HUMAN | 0.56 | 0.56 | 0.56 | 0.56 | 1.0 | 1.0 |
| tr:E7EN32:E7EN32_HUMAN | 0.9 | 0.9 | 0.9 | 0.9 | 1.0 | 1.0 |
| sp:O00255:MEN1_HUMAN | 1.0 | 1.0 | 1.0 | 1.0 | 1.0 | 1.0 |
| sp:Q9WVR8:MEN1_RAT | 0.96 | 0.98 | 0.96 | 0.98 | 1.0 | 1.0 |
| sp:O88559:MEN1_MOUSE | 0.96 | 0.98 | 0.96 | 0.98 | 1.0 | 1.0 |
| tr:F8WHD9:F8WHD9_MOUSE | 0.95 | 0.97 | 0.95 | 0.97 | 1.0 | 1.0 |
| tr:Q8SXU2:Q8SXU2_DROME | 0.36 | 0.64 | 0.37 | 0.65 | 0.57 | 0.68 |
| tr:Q7KXY9:Q7KXY9_DROME | 0.32 | 0.6 | 0.34 | 0.64 | 0.57 | 0.68 |
| tr:Q9VM47:Q9VM47_DROME | 0.32 | 0.59 | 0.34 | 0.64 | 0.57 | 0.68 |

**Mnn1 (FBgn0031885) associated phenotypes**

DNA repair defective, chemical sensitive, conditional, heat sensitive, lethal - all die before end of P-stage, melanotic mass phenotype, radiation sensitive, short lived, some die during pharate adult stage, stress response defective

(Information from FlyBase)

## Phosphoenolpyruvate carboxykinase, cytosolic [GTP]

Best gene implication in ageing for this target family came from gene Q9Z2V4 via mapping the annotation from Ensembl ENSMUSG00000027513 via mapping the annotation from EntrezGene 18534 via mapping the annotation from GenAgeModels 0497 annotated in GenAge release 17.

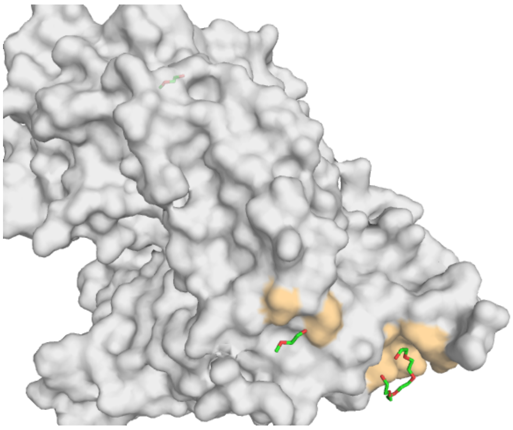

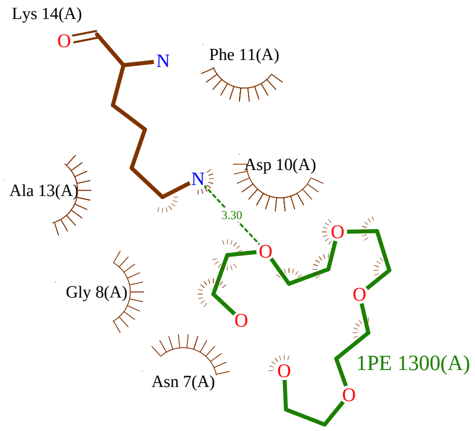


| protein | amino acids contacts (binding site) |
|---|---|
| PDB:3dt2:chainA:P07379 | N G D F A K Q A P L W W M K R E A S W L P K I |
| PDB:3dt4:chainA:P07379 | N G D F A K Q A P L W W M K R E A S W L P K I |
| PDB:3dt7:chainA:P07379 | N G D F A K Q A P L W W M K R E A S W L P K I |
| PDB:3dtb:chainA:P07379 | N G D F A K Q A P L W W M K R E A S W L P K I |
| PDB:3moe:chainA:P07379 | N G D F A K Q A P L W W M K R E A S W L P K I |
| PDB:3mof:chainA:P07379 | N G D F A K Q A P L W W M K R E A S W L P K I |
| PDB:3moh:chainA:P07379 | - - - - A K Q A P L W W M K R E A S W L P K I |
| PDB:4gno:chainA:P07379 | N G D F A K Q A P L W W M K R E A S W L P K I |
| PDB:4ox2:chainA:P07379 | N G D F A K Q A P L W W M K R E A S W L P K I |
| sp:P35558:PCKGC_HUMAN | N G N L A K Q A P L W W M K R E A S W L P K I |
| sp:P07379:PCKGC_RAT | N G D F A K Q A P L W W M K R E A S W L P K I |
| sp:Q9Z2V4:PCKGC_MOUSE | N G D F A K Q A P L W W M K R E A S W L P K I |
| sp:P20007:PCKG_DROME | Q S I I G N Q Q P L W W M K R A A D W V P K I |
| tr:O02286:O02286_CAEEL | T P K N G A R S P L W W M K Y Q T S W M P K I |

| protein | whole protein ident | whole protein simil | domain-based ident | domain-based simil | contact-based ident | contact-based simil |
|---|---|---|---|---|---|---|
| PDB:3dt2:chainA:P07379 | 1.0 | 1.0 | 1.0 | 1.0 | 1.0 | 1.0 |
| PDB:3dt4:chainA:P07379 | 1.0 | 1.0 | 1.0 | 1.0 | 1.0 | 1.0 |
| PDB:3dt7:chainA:P07379 | 1.0 | 1.0 | 1.0 | 1.0 | 1.0 | 1.0 |
| PDB:3dtb:chainA:P07379 | 1.0 | 1.0 | 1.0 | 1.0 | 1.0 | 1.0 |
| PDB:3moe:chainA:P07379 | 1.0 | 1.0 | 1.0 | 1.0 | 1.0 | 1.0 |
| PDB:3mof:chainA:P07379 | 1.0 | 1.0 | 0.99 | 1.0 | 1.0 | 1.0 |
| PDB:3moh:chainA:P07379 | 1.0 | 1.0 | 1.0 | 1.0 | 0.83 | 0.64 |
| PDB:4gno:chainA:P07379 | 1.0 | 1.0 | 0.99 | 1.0 | 1.0 | 1.0 |
| PDB:4ox2:chainA:P07379 | 1.0 | 1.0 | 1.0 | 1.0 | 1.0 | 1.0 |
| sp:P35558:PCKGC_HUMAN | 0.91 | 0.98 | 0.9 | 0.97 | 0.91 | 0.98 |
| sp:P07379:PCKGC_RAT | 1.0 | 1.0 | 1.0 | 1.0 | 1.0 | 1.0 |
| sp:Q9Z2V4:PCKGC_MOUSE | 0.98 | 0.99 | 0.97 | 0.99 | 1.0 | 1.0 |
| sp:P20007:PCKG_DROME | 0.61 | 0.84 | 0.6 | 0.83 | 0.57 | 0.69 |
| tr:O02286:O02286_CAEEL | 0.54 | 0.82 | 0.54 | 0.82 | 0.48 | 0.65 |

**Pepck (UniProt:P20007) annotation**
**Function**: Catalyzes the conversion of oxaloacetate (OAA) to phosphoenolpyruvate (PEP), the rate-limiting step in the metabolic pathway that produces glucose from lactate and other precursors derived from the citric acid cycle.
**Cofactor**: Mn(2+)Note=Binds 1 Mn(2+) ion per subunit. ;
**Pathway**: Carbohydrate biosynthesis; gluconeogenesis.
**Subunit**: Monomer.

(Information from UniProt)

## Alcohol dehydrogenase class-3

Best gene implication in ageing for this target family came from gene P12711 annotated in UniProt release 2014_02. Annotation GO 7568 (aging) was Inferred from Expression Pattern

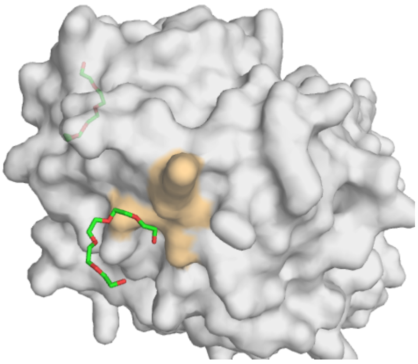

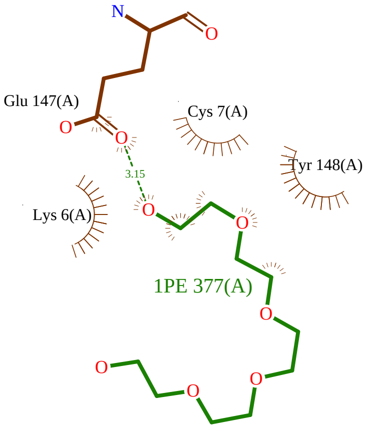


| protein | amino acids contacts (binding site) | | | | | | | | | |
|---|---|---|---|---|---|---|---|---|---|---|
| PDB:3qj5:chainA:P11766 | K | C | K | Q | C | G | E | Y | V | E |
| tr:Q6IRT1:Q6IRT1_HUMAN | K | C | K | Q | C | G | E | Y | V | E |
| sp:P11766:ADHX_HUMAN | K | C | K | Q | C | G | E | Y | V | E |
| sp:P12711:ADHX_RAT | R | C | K | Q | C | G | E | Y | V | E |
| sp:P28474:ADHX_MOUSE | R | C | K | Q | C | G | E | Y | V | E |
| tr:Q6P5I3:Q6P5I3_MOUSE | R | C | K | Q | C | G | E | Y | V | E |
| sp:P46415:ADHX_DROME | T | C | N | Q | C | N | E | Y | V | S |
| sp:Q17335:ADHX_CAEEL | N | C | G | Q | C | K | E | Y | V | E |
| sp:P32771:FADH_YEAST | K | C | T | E | C | G | E | Y | R | S |

| | whole protein | | domain-based | | contact-based | |
|---|---|---|---|---|---|---|
| protein | ident | simil | ident | simil | ident | simil |
| PDB:3qj5:chainA:P11766 | 1.0 | 1.0 | 1.0 | 1.0 | 1.0 | 1.0 |
| tr:Q6IRT1:Q6IRT1_HUMAN | 1.0 | 1.0 | 1.0 | 1.0 | 1.0 | 1.0 |
| sp:P11766:ADHX_HUMAN | 1.0 | 1.0 | 1.0 | 1.0 | 1.0 | 1.0 |
| sp:P12711:ADHX_RAT | 0.94 | 0.98 | 0.94 | 0.98 | 0.9 | 0.98 |
| sp:P28474:ADHX_MOUSE | 0.93 | 0.98 | 0.93 | 0.98 | 0.9 | 0.98 |
| tr:Q6P5I3:Q6P5I3_MOUSE | 0.93 | 0.98 | 0.93 | 0.98 | 0.9 | 0.98 |
| sp:P46415:ADHX_DROME | 0.7 | 0.9 | 0.71 | 0.91 | 0.6 | 0.69 |
| sp:Q17335:ADHX_CAEEL | 0.67 | 0.88 | 0.68 | 0.89 | 0.7 | 0.68 |
| sp:P32771:FADH_YEAST | 0.6 | 0.84 | 0.62 | 0.85 | 0.6 | 0.74 |

### Fdh (FBgn0011768) associated phenotypes

heat sensitive, memory defective

(Information from FlyBase)

### Fdh (UniProt:P46415) annotation

**Function**: Class-III ADH is remarkably ineffective in oxidizing ethanol, but it readily catalyzes the oxidation of long-chain primary alcohols and the oxidation of S-(hydroxymethyl) glutathione.
**Cofactor**:Zn(2+)Note=Binds 2 Zn(2+) ions per subunit. ;

(Information from UniProt)

### H24K24.3 (UniProt:Q17335) annotation

**Function**: Class-III ADH is remarkably ineffective in oxidizing ethanol, but it readily catalyzes the oxidation of long-chain primary alcohols and the oxidation of S-(hydroxymethyl) glutathione.
**Cofactor**: Zn(2+)Note=Binds 2 Zn(2+) ions per subunit. ;
**Subunit**: Homodimer.
**Subcellular location**: Cytoplasm

(Information from UniProt)

### 2',3'-cyclic-nucleotide 3'-phosphodiesterase

Best gene implication in ageing for this target family came from gene P13233 annotated in UniProt release 2014_02. Annotation GO 7568 (aging) was Inferred from Expression Pattern

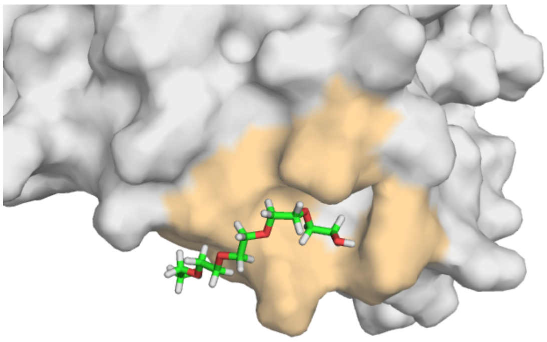

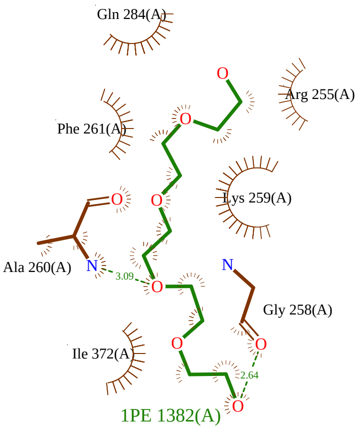


| protein | amino acids contacts (binding site) |
|---|---|
| PDB:2yoz:chainA:P16330 | A K E R G K A F Q V S I |
| PDB:2yp0:chainA:P16330 | A K E R G K A F Q V S I |
| sp:P09543:CN37_HUMAN | A K E K S K A F Q V S I |
| sp:P13233:CN37_RAT | A K E R G K A F Q V S I |
| tr:Q3TYL9:Q3TYL9_MOUSE | A K E R G K A F Q V S I |
| sp:P16330:CN37_MOUSE | A K E R G K A F Q V S I |
| tr:Q3TYV5:Q3TYV5_MOUSE | A K E R G K A F Q V S I |
| tr:Q19804:Q19804_CAEEL | - - - - - - - - - - - - |

| | whole protein | | domain-based | | contact-based | |
|---|---|---|---|---|---|---|
| protein | ident | simil | ident | simil | ident | simil |
| PDB:2yoz:chainA:P16330 | 1.0 | 1.0 | 1.0 | 1.0 | 1.0 | 1.0 |
| PDB:2yp0:chainA:P16330 | 1.0 | 1.0 | 1.0 | 1.0 | 1.0 | 1.0 |
| sp:P09543:CN37_HUMAN | 0.85 | 0.96 | 0.81 | 0.95 | 0.83 | 0.94 |
| sp:P13233:CN37_RAT | 0.95 | 0.99 | 0.95 | 0.99 | 1.0 | 1.0 |
| tr:Q3TYL9:Q3TYL9_MOUSE | 0.95 | 0.95 | 1.0 | 1.0 | 1.0 | 1.0 |
| sp:P16330:CN37_MOUSE | 1.0 | 1.0 | 1.0 | 1.0 | 1.0 | 1.0 |
| tr:Q3TYV5:Q3TYV5_MOUSE | 1.0 | 1.0 | 1.0 | 1.0 | 1.0 | 1.0 |
| tr:Q19804:Q19804_CAEEL | 0.11 | 0.28 | 0.02 | 0.04 | 0.0 | 0.0 |

### Peptidyl-prolyl cis-trans isomerase NIMA-interacting 1

Best gene implication in ageing for this target family came from gene Q13526 via mapping the annotation from Ensembl ENSG00000127445 via mapping the annotation from EntrezGene 5300 via mapping the annotation from GenAgeHuman 0062 annotated in GenAge release 17.

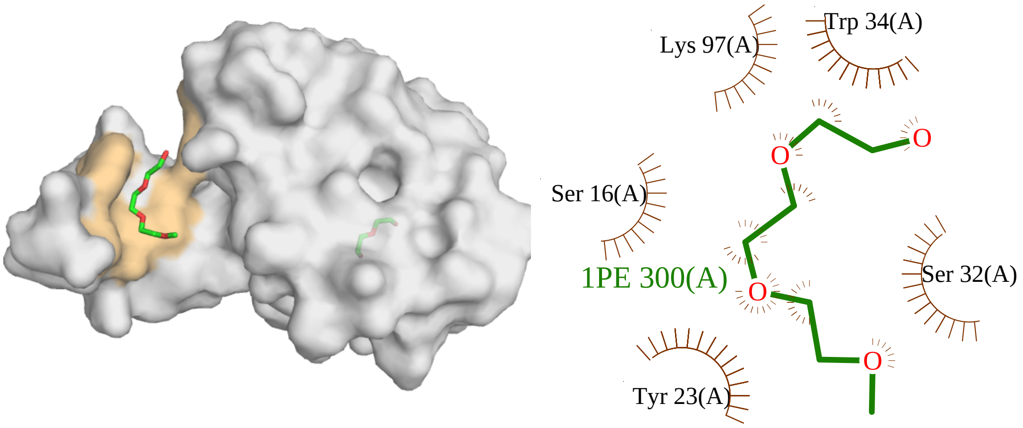


| protein | amino acids contacts (binding site) |
|---|---|
| PDB:1zcn:chainA:Q13526 | S Y S W H K L M Q F H |
| PDB:2f21:chainA:Q13526 | A Y S W H K L M Q F H |
| sp:Q13526:PIN1_HUMAN | S Y S W H K L M Q F H |
| tr:B0BNL2:B0BNL2_RAT | S Y S W H K L M Q F H |
| sp:Q9QUR7:PIN1_MOUSE | S Y S W H K L M Q F H |
| sp:P54353:DOD_DROME | S Y S W H V L M Q F H |
| tr:Q9N492:Q9N492_CAEEL | S Y S W H K L M Q F H |
| sp:P22696:ESS1_YEAST | S Y S W H D L M Q F H |

| | whole protein | | domain-based | | contact-based | |
|---|---|---|---|---|---|---|
| protein | ident | simil | ident | simil | ident | simil |
| PDB:1zcn:chainA:Q13526 | 0.99 | 1.0 | 0.99 | 1.0 | 1.0 | 1.0 |
| PDB:2f21:chainA:Q13526 | 0.99 | 0.99 | 0.99 | 0.99 | 0.91 | 0.91 |
| sp:Q13526:PIN1_HUMAN | 1.0 | 1.0 | 1.0 | 1.0 | 1.0 | 1.0 |
| tr:B0BNL2:B0BNL2_RAT | 0.96 | 0.98 | 0.99 | 1.0 | 1.0 | 1.0 |
| sp:Q9QUR7:PIN1_MOUSE | 0.95 | 0.98 | 0.98 | 1.0 | 1.0 | 1.0 |
| sp:P54353:DOD_DROME | 0.55 | 0.82 | 0.58 | 0.84 | 0.91 | 0.91 |
| tr:Q9N492:Q9N492_CAEEL | 0.54 | 0.81 | 0.57 | 0.84 | 1.0 | 1.0 |
| sp:P22696:ESS1_YEAST | 0.44 | 0.78 | 0.46 | 0.8 | 0.91 | 0.91 |

### Protein deglycase DJ-1

Best gene implication in ageing for this target family came from gene Q6NMT9 via mapping the annotation from Ensembl FBgn0033885 via mapping the annotation from EntrezGene 36543 via mapping the annotation from GenAgeModels 0178 annotated in GenAge release 17.

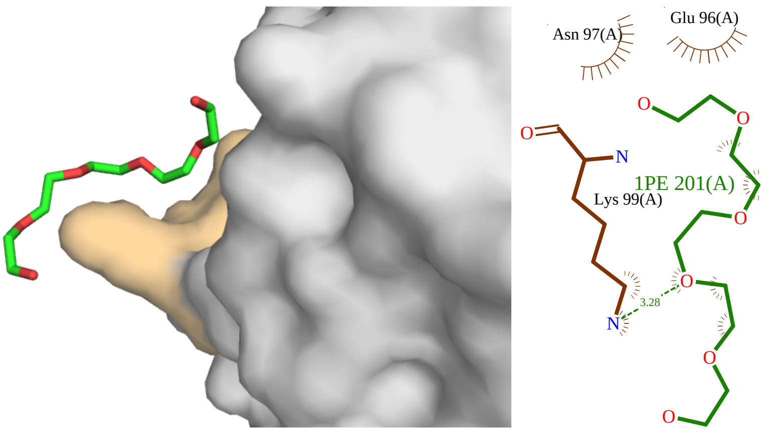

| protein | amino acids contacts (binding site) |
|---|---|
| PDB:4p2g:chainA:Q99497 | E N K |
| PDB:4p34:chainA:Q99497 | E N K |
| tr:K7ELW0:K7ELW0_HUMAN | E N K |
| tr:K7EN27:K7EN27_HUMAN | E N K |
| sp:Q99497:PARK7_HUMAN | E N K |
| sp:O88767:PARK7_RAT | E N K |
| tr:A2A815:A2A815_MOUSE | E S K |
| tr:A2A813:A2A813_MOUSE | E S K |
| sp:Q99LX0:PARK7_MOUSE | E S K |
| tr:Q6NMT9:Q6NMT9_DROME | E S G |
| tr:Q9VA37:Q9VA37_DROME | E S G |
| tr:A1Z9J4:A1Z9J4_DROME | E S G |
| tr:P90994:P90994_CAEEL | V E G |
| tr:O16228:O16228_CAEEL | V K G |

| protein | whole protein ident | whole protein simil | domain-based ident | domain-based simil | contact-based ident | contact-based simil |
|---|---|---|---|---|---|---|
| PDB:4p2g:chainA:Q99497 | 0.99 | 1.0 | 0.99 | 1.0 | 1.0 | 1.0 |
| PDB:4p34:chainA:Q99497 | 0.99 | 1.0 | 0.99 | 1.0 | 1.0 | 1.0 |
| tr:K7ELW0:K7ELW0_HUMAN | 0.89 | 0.89 | 0.89 | 0.89 | 1.0 | 1.0 |
| tr:K7EN27:K7EN27_HUMAN | 0.75 | 0.75 | 0.76 | 0.76 | 1.0 | 1.0 |
| sp:Q99497:PARK7_HUMAN | 1.0 | 1.0 | 1.0 | 1.0 | 1.0 | 1.0 |
| sp:O88767:PARK7_RAT | 0.92 | 0.98 | 0.91 | 0.98 | 1.0 | 1.0 |
| tr:A2A815:A2A815_MOUSE | 0.67 | 0.7 | 0.67 | 0.71 | 0.67 | 0.79 |
| tr:A2A813:A2A813_MOUSE | 0.83 | 0.9 | 0.84 | 0.91 | 0.67 | 0.79 |
| sp:Q99LX0:PARK7_MOUSE | 0.92 | 0.98 | 0.91 | 0.98 | 0.67 | 0.79 |
| tr:Q6NMT9:Q6NMT9_DROME | 0.35 | 0.5 | 0.42 | 0.6 | 0.33 | 0.41 |
| tr:Q9VA37:Q9VA37_DROME | 0.47 | 0.74 | 0.52 | 0.82 | 0.33 | 0.41 |
| tr:A1Z9J4:A1Z9J4_DROME | 0.46 | 0.69 | 0.54 | 0.81 | 0.33 | 0.41 |
| tr:P90994:P90994_CAEEL | 0.52 | 0.82 | 0.52 | 0.82 | 0.0 | 0.41 |
| tr:O16228:O16228_CAEEL | 0.43 | 0.78 | 0.44 | 0.79 | 0.0 | 0.41 |

### dj-1beta (FBgn0039802) associated phenotypes

chemical resistant, chemical sensitive, locomotor behavior defective, long lived, oxidative stress response defective, radiation sensitive, short lived, stress response defective

(Information from FlyBase)

### DJ-1alpha (FBgn0033885) associated phenotypes

chemical resistant, chemical sensitive, increased cell death, locomotor behavior defective, short lived, stress response defective

(Information from FlyBase)

### djr-1.1 (WBGene00015184) associated phenotypes

solitary feeding increased

(Information from WormBase)

### djr-1.1 (UniProt:P90994) annotation

**Function**: Catalyzes the conversion of methylglyoxal (MG) or glyoxal (GO) to D-lactate or glycolic acid respectively in a single glutathione (GSH)-independent step. May play a role in detoxifying endogenously produced glyoxals. Involved in protection against glyoxal-induced cell death. (PubMed:22523093).
**Biophysicochemical properties**: Kinetic parameters: KM=0.3 mM for methylglyoxal (PubMed:22523093); KM=3.61 mM for glyoxal (PubMed:22523093); Note=kcat is 13.8 min(-1) with methylglyoxal as substrate and 356.4 min(-1) with glyoxal as substrate. (PubMed:22523093);
**Subcellular location**: Cytoplasm (PubMed:22523093). Nucleus (PubMed:22523093). Note=Ubiquitously localized throughout the whole cells with higher expression in the nucleus. (PubMed:22523093).
**Tissue specificity**: Expressed exclusively in the intestine. (PubMed:22523093).

(Information from UniProt)

**djr-1.2 (UniProt:O16228) annotation**

**Function**: Catalyzes the conversion of methylglyoxal (MG) or glyoxal (GO) to D-lactate or glycolic acid respectively in a single glutathione (GSH)-independent step. May play a role in detoxifying endogenously produced glyoxals. Involved in protection against glyoxal-induced cell death. Protects dopaminergic neurons from glyoxal-dependent neuronal degeneration. (PubMed:22523093, PubMed:23624124).

**Biophysicochemical properties**: Kinetic parameters: KM=0.39 mM for methylglyoxal (PubMed:22523093); KM=0.78 mM for glyoxal (PubMed:22523093); Note=kcat is 60.0 min(-1) with methylglyoxal as substrate and 146.4 min(-1) with glyoxal as substrate. (PubMed:22523093);

**Subcellular location**: Cytoplasm (PubMed:22523093).

**Tissue specificity**: Expressed in various tissues, including pharyngeal muscles, pharynx-intestinal valve, ventral nerve cord, spermatheca, rectal gland, inner labial (IL) cells of head neurons, phasmid (PHA/PHB) neurons in tail and supporting sheath/socket cells, as well as in head mesodermal cells (HMC), excretory canals and coelomocytes. (PubMed:22523093).

**Induction**: Induced by DAF-16 during starvation as well as in the dauer stage. (PubMed:23624124).

(Information from UniProt)
