# Supplementary material for: Drug repurposing for aging research using model organisms
Source: Aging Cell. 2017 Jun 16;16(5):1006–15. doi: 10.1111/acel.12626 (PMC5595691; doi:10.1111/acel.12626)
Supplement: Supplementary file 7 — Data S1 Zip‐Archive of all report cards. [file ACEL-16-1006-s007.zip › RC_1ZB.pdf]

# 1ZB

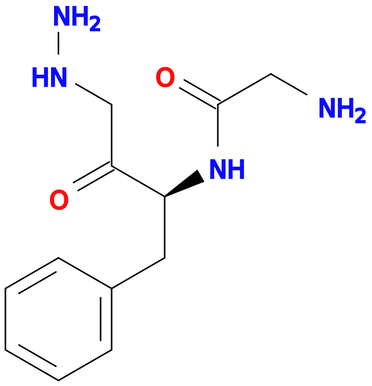

**Database identifiers**

| | |
|---|---|
| ChEMBLCompound | CHEMBL448143 |
| eMolecules | 27275031 |

## Ranking

| | Rank | Score |
|---|---|---|
| Drosophila | NA | NA |
| C. elegans | 411/591 | 0.13 |

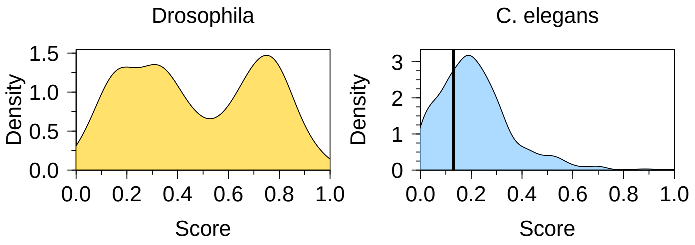


| | Ageing implication | Domain conservation | Binding site conservation | Binding affinity | Bioavailability | Lipinski | Promiscuity | Purchasability | Drug approval | **Total** |
|---|---|---|---|---|---|---|---|---|---|---|
| Drosophila | NA | NA | NA | NA | NA | NA | NA | NA | NA | NA |
| C. elegans | 0.792 | 0.412 | 0.674 | 0.538 | 0.254 | 0.0 | -0.0 | 0.1 | 0.0 | 0.13 |

## Names

No synonyms found

## Roles

ChEBI entry None has no roles

## Status

| | |
|---|---|
| Approved drug (according to ChEMBL) | No |
| Number of Rule of 5 violations | 0 |
| Binding affinity to original target in log units (RF-Score prediction) | 5.15 |
| Burns *C. elegans* bioavailability prediction | -1.93 |

## Compound Target Characteristics

### Dipeptidyl peptidase 1

Best gene implication in ageing for this target family came from gene P80067 annotated in UniProt release 2014_02. Annotation GO 7568 (aging) was Inferred from Expression Pattern

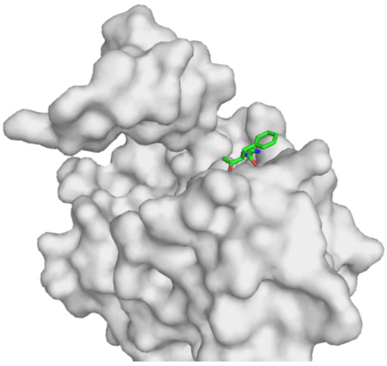

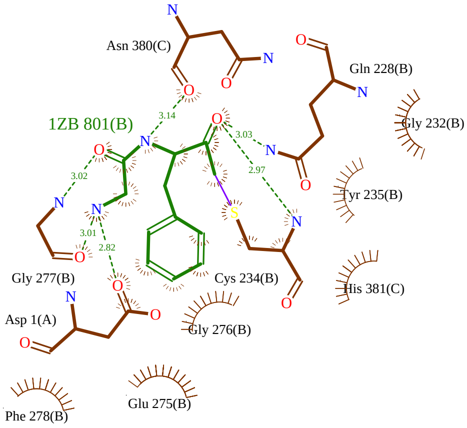


| protein | amino acids contacts (binding site) | | | | | | | | | | | |
|---|---|---|---|---|---|---|---|---|---|---|---|---|
| PDB:2djf:chainA:P53634 | D | - | - | - | - | - | - | - | - | - | - | - |
| PDB:2djf:chainB:P53634 | - | Q | G | S | C | Y | E | G | G | F | - | - |
| PDB:2djf:chainC:P53634 | - | - | - | - | - | - | - | - | - | - | N | H |
| sp:P53634:CATC_HUMAN | D | Q | G | S | C | Y | E | G | G | F | N | H |
| sp:P80067:CATC_RAT | D | Q | G | S | C | Y | D | G | G | F | N | H |
| tr:Q3U9B7:Q3U9B7_MOUSE | D | Q | G | S | C | Y | D | G | G | F | N | H |
| sp:P97821:CATC_MOUSE | D | Q | G | S | C | Y | D | G | G | F | N | H |
| tr:Q3UBY5:Q3UBY5_MOUSE | D | Q | G | S | C | Y | D | G | G | F | N | H |
| tr:P92005:P92005_CAEEL | - | Q | G | S | C | W | Q | G | G | E | N | H |

| | whole protein | | domain-based | | contact-based | |
|---|---|---|---|---|---|---|
| protein | ident | simil | ident | simil | ident | simil |
| PDB:2djf:chainA:P53634 | 1.0 | 1.0 | 1.0 | 1.0 | 0.08 | 0.0 |
| PDB:2djf:chainB:P53634 | 1.0 | 1.0 | 1.0 | 1.0 | 0.75 | 0.5 |
| PDB:2djf:chainC:P53634 | 1.0 | 1.0 | 1.0 | 1.0 | 0.17 | 0.0 |
| sp:P53634:CATC_HUMAN | 1.0 | 1.0 | 1.0 | 1.0 | 1.0 | 1.0 |
| sp:P80067:CATC_RAT | 0.79 | 0.93 | 0.79 | 0.92 | 0.92 | 0.97 |
| tr:Q3U9B7:Q3U9B7_MOUSE | 0.76 | 0.9 | 0.76 | 0.9 | 0.92 | 0.97 |
| sp:P97821:CATC_MOUSE | 0.78 | 0.93 | 0.78 | 0.93 | 0.92 | 0.97 |
| tr:Q3UBY5:Q3UBY5_MOUSE | 0.78 | 0.93 | 0.78 | 0.93 | 0.92 | 0.97 |
| tr:P92005:P92005_CAEEL | 0.21 | 0.5 | 0.25 | 0.56 | 0.67 | 0.67 |
