# Supplementary material for: Drug repurposing for aging research using model organisms
Source: Aging Cell. 2017 Jun 16;16(5):1006–15. doi: 10.1111/acel.12626 (PMC5595691; doi:10.1111/acel.12626)
Supplement: Supplementary file 7 — Data S1 Zip‐Archive of all report cards. [file ACEL-16-1006-s007.zip › RC_21B.pdf]

21B

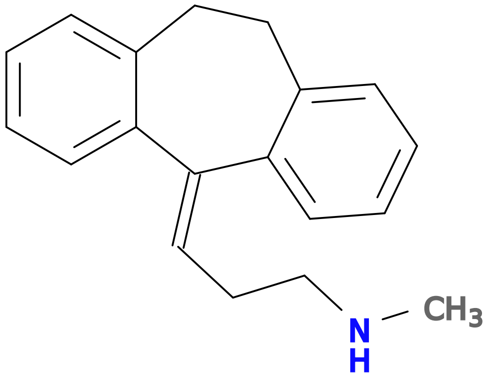

Database identifiers

ChEMBLCompound	CHEMBL445
DrugBank	DB00540
CHEBI	7640

Ranking

	Rank	Score
Drosophila	70/697	0.809
C. elegans	87/591	0.337

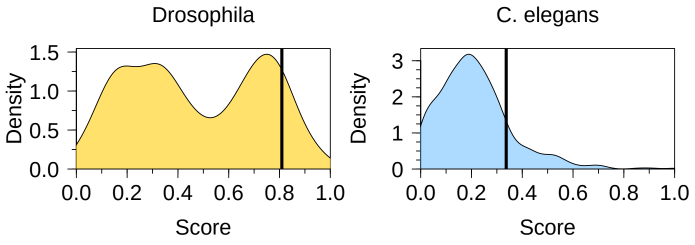

	Ageing implication	Domain conservation	Binding site conservation	Binding affinity	Bioavailability	Lipinski	Promiscuity	Purchasability	Drug approval	**Total**
Drosophila	0.792	0.982	1.0	0.871	(0.9)	0.0	-0.0	0.1	0.1	0.809
C. elegans	0.792	0.949	0.955	0.871	0.22	0.0	-0.0	0.1	0.1	0.337

Names

- **nortriptyline**
- Ateben
- Avantyl
- demethylamitriptyline
- Noritren
- Pamelor
- Psychostyl
- Sensaval

Roles

antidepressant, drug metabolite

Status

Approved drug (according to ChEMBL)	Yes
Administration Route	Oral
Number of Rule of 5 violations	0
Binding affinity to original target in log units (RF-Score prediction)	6.91
Burns *C. elegans* bioavailability prediction	-4.28

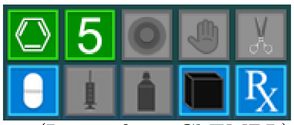
(Image from ChEMBL)

Medical Information

Indicatation: For the treatment of depression, chronic pain, irritable bowel syndrome, sleep disorders, diabetic neuropathy, agitation and insomnia, and migraine prophylaxis.
Mechanism of action: It is believed that nortriptyline either inhibits the reuptake of the neurotransmitter serotonin at the neuronal membrane or acts at beta-adrenergic receptors. Tricyclic antidepressants do not inhibit monoamine oxidase nor do they affect dopamine reuptake.
Toxicity: Symptoms of overdose include cardiac dysrhythmias, severe hypotension, shock, congestive heart failure, pulmonary edema, convulsions, and CNS depression, including coma. Changes in the electrocardiogram, particularly in QRS axis or width, are clinically significant indicators of tricyclic antidepressant toxicity.
Metabolism: Undergoes hepatic metabolism via the same pathway as other TCAs.
Retail: Advanced Pharmaceutical Services Inc., Amerisource Health Services Corp., Apotheca Inc., A-S Medication Solutions LLC, Bryant Ranch Prepack, Caremark LLC and 38 more suppliers
Nortriptyline hcl 75 mg capsule - 1.33 USD / capsule
Nortriptyline hcl powder - 11.09 USD / g
Pamelor 75 mg capsule - 19.34 USD / capsule
Pamelor 10 mg capsule - 24.04 USD / capsule
Pamelor 25 mg capsule - 24.04 USD / capsule
Pamelor 50 mg capsule - 24.04 USD / capsule
and 19 more options

(Information from DrugBank)

Compound Target Characteristics

Transporter

Best gene implication in ageing for this target family came from gene P23977 annotated in UniProt release 2014_02. Annotation GO 7568 (aging) was Inferred from Expression Pattern

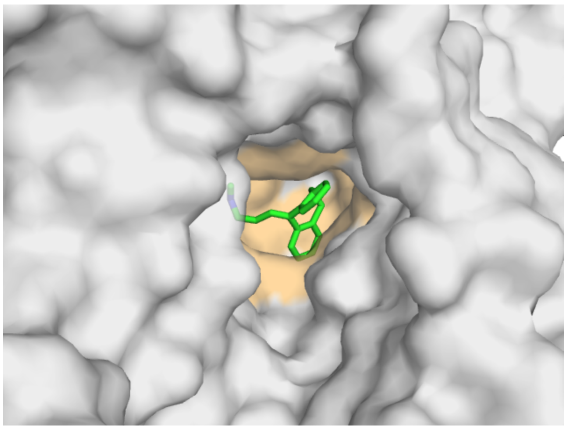

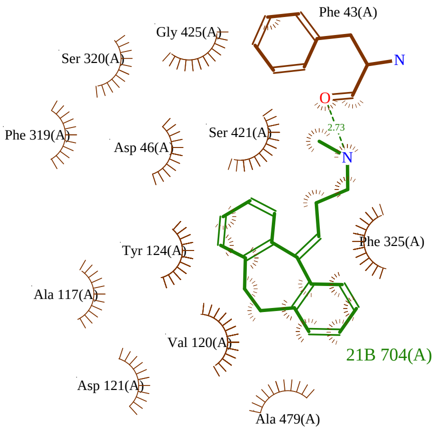

protein	amino acids contacts (binding site)
PDB:4m48:chainA:Q7K4Y6	F D A V D Y F S F S G A
sp:Q01959:SC6A3_HUMAN	F D S V G Y F S F S G A
sp:P23977:SC6A3_RAT	F D S V G Y F S F S G A
sp:Q61327:SC6A3_MOUSE	F D S V G Y F S F S G A
tr:Q9NB97:Q9NB97_DROME	F D A V D Y F S F S G A
tr:Q7K4Y6:Q7K4Y6_DROME	F D A V D Y F S F S G A
sp:Q03614:NTDO_CAEEL	F D A V D Y F S F S G T

	whole protein		domain-based		contact-based	
protein	ident	simil	ident	simil	ident	simil
PDB:4m48:chainA:Q7K4Y6	0.99	1.0	0.99	1.0	1.0	1.0
sp:Q01959:SC6A3_HUMAN	0.46	0.75	0.6	0.87	0.83	0.85
sp:P23977:SC6A3_RAT	0.47	0.75	0.61	0.88	0.83	0.85
sp:Q61327:SC6A3_MOUSE	0.47	0.75	0.61	0.88	0.83	0.85
tr:Q9NB97:Q9NB97_DROME	1.0	1.0	1.0	1.0	1.0	1.0
tr:Q7K4Y6:Q7K4Y6_DROME	1.0	1.0	1.0	1.0	1.0	1.0
sp:Q03614:NTDO_CAEEL	0.49	0.77	0.65	0.89	0.92	0.95

DAT (FBgn0034136) associated phenotypes

chemical conditional, circadian rhythm defective, hyperactive, locomotor behavior defective, sleep defective

(Information from FlyBase)

dat-1 (WBGene00000934) associated phenotypes

aqueous adaptation defective, swimming induced paralysis

(Information from WormBase)

dat-1 (UniProt:Q03614) annotation

Function: Terminates the action of dopamine by its high affinity sodium-dependent reuptake into presynaptic terminals. (PubMed:9765501).

Subcellular location: Membrane ; Multi-pass membrane protein

Miscellaneous: This protein is the target of psychomotor stimulants such as amphetamines or cocaine.

(Information from UniProt)
