# Supplementary material for: Drug repurposing for aging research using model organisms
Source: Aging Cell. 2017 Jun 16;16(5):1006–15. doi: 10.1111/acel.12626 (PMC5595691; doi:10.1111/acel.12626)
Supplement: Supplementary file 7 — Data S1 Zip‐Archive of all report cards. [file ACEL-16-1006-s007.zip › RC_255.pdf]

255

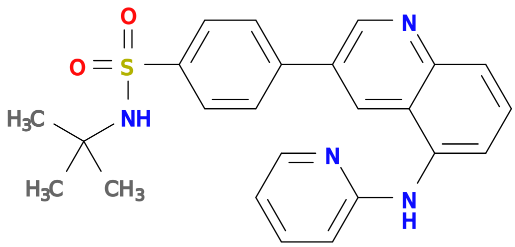

Database identifiers

ChEMBLCompound	CHEMBL250409
DrugBank	DB06933

Ranking

	Rank	Score
Drosophila	127/697	0.766
C. elegans	301/591	0.19

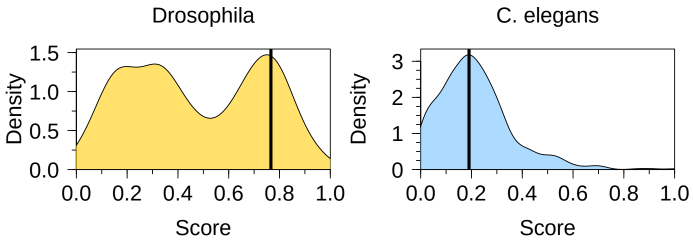

	Ageing implication	Domain conservation	Binding site conservation	Binding affinity	Bioavailability	Lipinski	Promiscuity	Purchasability	Drug approval	**Total**
Drosophila	1.0	0.971	0.987	0.86	(0.9)	-0.05	-0.0	0.0	0.075	0.766
C. elegans	1.0	0.969	0.912	0.86	0.218	-0.05	-0.0	0.0	0.075	0.19

Names

No synonyms found

Roles

ChEBI entry None has no roles

Status

Approved drug (according to ChEMBL)	No
Classification (according to DrugBank)	experimental
Number of Rule of 5 violations	1
Binding affinity to original target in log units (RF-Score prediction)	6.82
Burns *C. elegans* bioavailability prediction	-4.44

Compound Target Characteristics

Mitogen-activated protein kinase 10

Best gene implication in ageing for this target family came from gene Q8WQG9 annotated in UniProt release 2014_02. Annotation GO 8340 (determination of adult lifespan) was Inferred from Mutant Phenotype

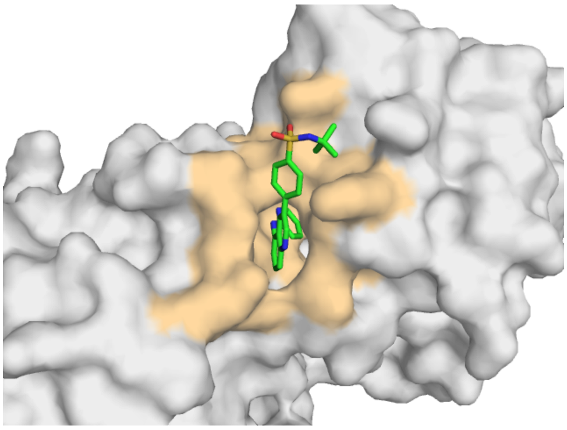

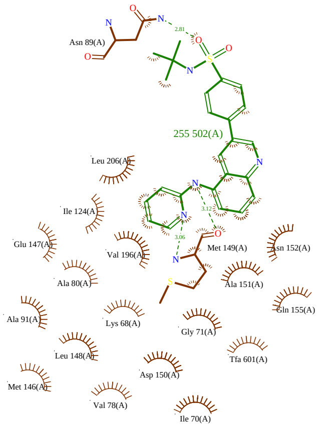

protein	amino acids contacts (binding site)
PDB:2r9s:chainA:P53779	K I G V A N A I M E L M D A N Q V L
tr:A6NF29:A6NF29_HUMAN	K I G V A N A I M E L M D A N Q V L
tr:D7R525:D7R525_HUMAN	K I G V A N A I M E L M D A N Q V L
tr:J3KNK1:J3KNK1_HUMAN	K I G V A N A I M E L M D A N Q V L
sp:P45983:MK08_HUMAN	K I G V A N A I M E L M D A N Q V L
sp:P45984:MK09_HUMAN	K I G V A N A I M E L M D A N Q V L
tr:A1L4K2:A1L4K2_HUMAN	K I G V A N A I M E L M D A N Q V L
sp:P53779:MK10_HUMAN	K I G V A N A I M E L M D A N Q V L
tr:F1LP66:F1LP66_RAT	K I G V A N A I M E L M D A N Q V L
tr:D4A5V8:D4A5V8_RAT	K I G V A N A I M E L M D A N Q V L
sp:P49186:MK09_RAT	K I G V A N A I M E L M D A N Q V L
tr:B0VXR6:B0VXR6_RAT	K I G V A N A I M E L M D A N Q V L
tr:D3ZQ33:D3ZQ33_RAT	K I G V A N A I M E L M D A N Q V L
tr:D3Z1Z4:D3Z1Z4_MOUSE	K I G V A N A I M E L M D A N Q V L
tr:A6P3E4:A6P3E4_MOUSE	K I G V A N A I M E L M D A N Q V L
sp:Q91Y86:MK08_MOUSE	K I G V A N A I M E L M D A N Q V L
tr:Q544A0:Q544A0_MOUSE	K I G V A N A I M E L M D A N Q V L
tr:Q3TQZ7:Q3TQZ7_MOUSE	K I G V A N A I M E L M D A N Q V L
tr:Q80W82:Q80W82_MOUSE	K I G V A N A I M E L M D A N Q V L
tr:Q7TSJ7:Q7TSJ7_MOUSE	K I G V A N A I M E L M D A N Q V L
tr:Q78GB8:Q78GB8_MOUSE	K I G V A N A I M E L M D A N Q V L
sp:Q9WTU6:MK09_MOUSE	K I G V A N A I M E L M D A N Q V L
tr:G3X8U9:G3X8U9_MOUSE	K I G V A N A I M E L M D A N Q V L
tr:Q5NCK8:Q5NCK8_MOUSE	K I G V A N A I M E L M D A N Q V L
tr:Q80W80:Q80W80_MOUSE	K I G V A N A I M E L M D A N Q V L
tr:Q8C9D4:Q8C9D4_MOUSE	K I G V A N A I M E L M D A N Q V L
tr:E9QN59:E9QN59_MOUSE	K I G V A N A I M E L M D A N Q V L
tr:E1JHD6:E1JHD6_DROME	R I G V A N A I M E L M D A N Q V L
sp:P92208:JNK_DROME	R I G V A N A I M E L M D A N Q V L
sp:Q8WQG9:JNK1_CAEEL	R I G V S Q A I M E L M D A N Q V L
sp:P32485:HOG1_YEAST	N V G V S P A I T E L Q G T D R L C

protein	whole protein ident	simil	domain-based ident	simil	contact-based ident	simil
PDB:2r9s:chainA:P53779	0.99	0.99	0.99	0.99	1.0	1.0
tr:A6NF29:A6NF29_HUMAN	0.43	0.46	0.69	0.73	1.0	1.0
tr:D7R525:D7R525_HUMAN	0.41	0.44	0.66	0.7	1.0	1.0
tr:J3KNK1:J3KNK1_HUMAN	0.63	0.7	0.76	0.8	1.0	1.0
sp:P45983:MK08_HUMAN	0.83	0.89	0.96	0.99	1.0	1.0
sp:P45984:MK09_HUMAN	0.76	0.86	0.91	0.97	1.0	1.0
tr:A1L4K2:A1L4K2_HUMAN	0.82	0.89	0.94	0.99	1.0	1.0
sp:P53779:MK10_HUMAN	1.0	1.0	1.0	1.0	1.0	1.0
tr:F1LP66:F1LP66_RAT	0.76	0.84	0.89	0.96	1.0	1.0
tr:D4A5V8:D4A5V8_RAT	0.77	0.86	0.93	0.98	1.0	1.0
sp:P49186:MK09_RAT	0.76	0.86	0.91	0.97	1.0	1.0
tr:B0VXR6:B0VXR6_RAT	0.92	0.92	1.0	1.0	1.0	1.0
tr:D3ZQ33:D3ZQ33_RAT	0.99	1.0	1.0	1.0	1.0	1.0
tr:D3Z1Z4:D3Z1Z4_MOUSE	0.61	0.65	0.78	0.81	1.0	1.0
tr:A6P3E4:A6P3E4_MOUSE	0.74	0.8	0.94	0.99	1.0	1.0
sp:Q91Y86:MK08_MOUSE	0.75	0.81	0.96	0.99	1.0	1.0
tr:Q544A0:Q544A0_MOUSE	0.75	0.81	0.96	0.99	1.0	1.0
tr:Q3TQZ7:Q3TQZ7_MOUSE	0.89	0.9	1.0	1.0	1.0	1.0
tr:Q80W82:Q80W82_MOUSE	0.89	0.9	1.0	1.0	1.0	1.0
tr:Q7TSJ7:Q7TSJ7_MOUSE	0.83	0.89	0.96	0.99	1.0	1.0
tr:Q78GB8:Q78GB8_MOUSE	0.92	0.92	1.0	1.0	1.0	1.0
sp:Q9WTU6:MK09_MOUSE	0.77	0.86	0.93	0.98	1.0	1.0
tr:G3X8U9:G3X8U9_MOUSE	0.82	0.89	0.94	0.99	1.0	1.0
tr:Q5NCK8:Q5NCK8_MOUSE	0.76	0.86	0.91	0.97	1.0	1.0
tr:Q80W80:Q80W80_MOUSE	0.98	0.99	0.97	0.99	1.0	1.0
tr:Q8C9D4:Q8C9D4_MOUSE	1.0	1.0	1.0	1.0	1.0	1.0
tr:E9QN59:E9QN59_MOUSE	0.94	0.94	1.0	1.0	1.0	1.0
tr:E1JHD6:E1JHD6_DROME	0.62	0.74	0.84	0.95	0.94	0.99
sp:P92208:JNK_DROME	0.62	0.74	0.84	0.95	0.94	0.99
sp:Q8WQG9:JNK1_CAEEL	0.45	0.6	0.8	0.94	0.83	0.91
sp:P32485:HOG1_YEAST	0.29	0.64	0.47	0.81	0.33	0.56

bsk (FBgn0000229) associated phenotypes

RU486 conditional, cell death defective, chemical resistant, chemical sensitive, conditional, decreased cell growth, dominant, heat sensitive, increased cell death, long lived, neuroanatomy defective, neurophysiology defective, nutrition conditional, oxidative stress response defective, planar polarity defective, progressive, short lived, somatic clone, starvation stress response defective, stress response defective, wound healing defective

(Information from FlyBase)

bsk (UniProt:P92208) annotation

Function: Responds to activation by environmental stress by phosphorylating a number of transcription factors, primarily components of AP-1 such as Jra and also the transcriptional repressor aop, and thus regulates transcriptional activity. Component of the immune response activated by bacterial infection, and is involved in wound healing and in dorsal closure, a morphogenetic movement during embryogenesis. Controls the expression of a phosphatase, puckered, at the edges of wounded epidermal tissue and in the dorsal epithelium during dorsal closure. (PubMed:10433922, PubMed:11784101, PubMed:8946915, PubMed:9224720).

Cofactor:Mg(2+)

Enzyme regulation: Activated by threonine and tyrosine phosphorylation by the dual specificity kinase, hep. Inhibited by dual specificity phosphatase, puckered. (PubMed:10433922).

Subcellular location: Cytoplasm

Tissue specificity: During gastrulation, expression is seen in cells undergoing morphogenetic movements. By stage 9 of embryonic development, expression is ubiquitous. At stages 12-14, expression occurs in epidermis and central nervous system. At stage 15, expression is restricted to ventral nerve cord, brain and some peripheral neurons. In larvae, expression is seen in all imaginal disks, with highest levels in wing and eye disks, and in the CNS. Adults express the protein in fat body and hemocytes. (PubMed:8946915, PubMed:8946916).

Developmental stage: Expressed maternally and zygotically through to adult (male and female). (PubMed:8946915).
Domain: The TXY motif contains the threonine and tyrosine residues whose phosphorylation activates the MAP kinases.
Ptm: Dually phosphorylated on Thr-181 and Tyr-183, which activates the enzyme.

(Information from UniProt)

jnk-1 (WBGene00002178) associated phenotypes

aldicarb hypersensitive, pathogen resistance increased, protein phosphorylation reduced, shortened life span, thermotolerance reduced

(Information from WormBase)

jnk-1 (UniProt:Q8WQG9) annotation

Function: Responds to activation by environmental stress by phosphorylating a number of transcription factors, and thus regulates transcriptional activity (By similarity). May coordinate locomotion by acting via type-D GABAergic motor neurons and regulate synaptic vesicle transport in conjunction with unc-16. (, PubMed:10393177, PubMed:11738026).
Cofactor:Mg(2+) Evidence=(PubMed:10393177);
Enzyme regulation: Activated by threonine and tyrosine phosphorylation by either of the dual specificity kinases, jkk-1 and sek-1. (PubMed:10393177).
Subunit: Binds to the scaffolding protein, unc-16. Unc-16 also binds other components of the JNK signaling pathway. (PubMed:11738026).
Domain: The TXY motif contains the threonine and tyrosine residues whose phosphorylation activates the MAP kinases.
Ptm: Dually phosphorylated on Thr-276 and Tyr-278, which activates the enzyme.

(Information from UniProt)
