# Supplementary material for: Drug repurposing for aging research using model organisms
Source: Aging Cell. 2017 Jun 16;16(5):1006–15. doi: 10.1111/acel.12626 (PMC5595691; doi:10.1111/acel.12626)
Supplement: Supplementary file 7 — Data S1 Zip‐Archive of all report cards. [file ACEL-16-1006-s007.zip › RC_3BK.pdf]

3BK

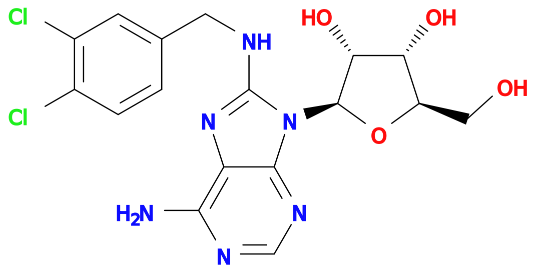

Database identifiers

ChEMBLCompound	CHEMBL516357
DrugBank	DB07045

Ranking

	Rank	Score
Drosophila	84/697	0.798
C. elegans	216/591	0.237

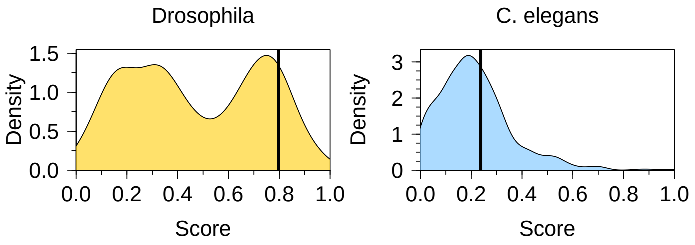

	Ageing implication	Domain conservation	Binding site conservation	Binding affinity	Bioavailability	Lipinski	Promiscuity	Purchasability	Drug approval	**Total**
Drosophila	1.0	0.977	1.0	0.822	(0.9)	0.0	-0.0	0.0	0.075	0.798
C. elegans	1.0	0.976	1.0	0.822	0.203	0.0	-0.0	0.0	0.075	0.237

Names

No synonyms found

Roles

ChEBI entry None has no roles

Status

Approved drug (according to ChEMBL)	No
Classification (according to DrugBank)	experimental
Number of Rule of 5 violations	0
Binding affinity to original target in log units (RF-Score prediction)	6.53
Burns *C. elegans* bioavailability prediction	-8.69

Compound Target Characteristics

Heat shock cognate 71 kDa protein

Best gene implication in ageing for this target family came from gene O97125 annotated in UniProt release 2014_02. Annotation GO 8340 (determination of adult lifespan) was Inferred from Mutant Phenotype

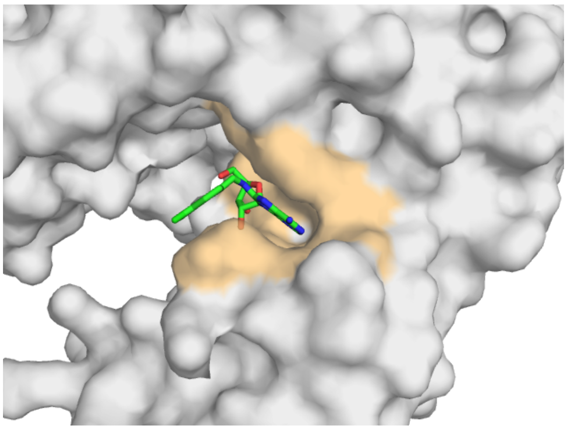

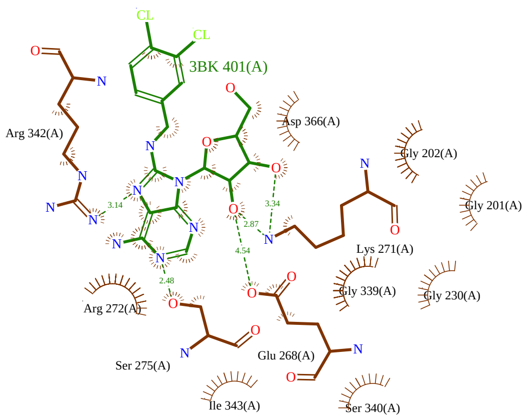

protein	amino acids contacts (binding site)
PDB:3fzk:chainA:P11142	G G G E K R S G S R I D
tr:E9PN89:E9PN89_HUMAN	G G G E K R S G S R I D
tr:E7EP94:E7EP94_HUMAN	G G G E K R S G S R I D
tr:Q96IS6:Q96IS6_HUMAN	G G G E K R S G S R I D
tr:E9PKE3:E9PKE3_HUMAN	G G G E K R S G S R I D
sp:P08107:HSP71_HUMAN	G G G E K R S G S R I D
sp:P11142:HSP7C_HUMAN	G G G E K R S G S R I D
tr:A8K5I0:A8K5I0_HUMAN	G G G E K R S G S R I D
tr:M0R8M9:M0R8M9_RAT	G G G E K R S G S R I D
tr:F1LZI1:F1LZI1_RAT	G G G E K R S G S R I D
tr:M0RCB1:M0RCB1_RAT	G G G E K R S G S R I D
tr:Q3TB63:Q3TB63_MOUSE	G G G E K R S G S R I D
tr:Q3KQJ4:Q3KQJ4_MOUSE	G G G E K R S G S R I D
tr:Q504P4:Q504P4_MOUSE	G G G E K R S G S R I D
sp:P63017:HSP7C_MOUSE	G G G E K R S G S R I D
tr:Q8I0E9:Q8I0E9_DROME	G G G E K R S G S R I D
sp:O97125:HSP68_DROME	G G G E K R S G S R I D
sp:P29843:HSP7A_DROME	G G G E K R S G S R I D
tr:F3YDI6:F3YDI6_DROME	G G G E K R S G S R I D
sp:P82910:HSP70_DROME	G G G E K R S G S R I D
sp:P02825:HSP71_DROME	G G G E K R S G S R I D
tr:C7LA76:C7LA76_DROME	G G G E K R S G S R I D
sp:P29844:HSP7C_DROME	G G G E K R S G S R I D
sp:P11147:HSP7D_DROME	G G G E K R S G S R I D
tr:C7LA75:C7LA75_DROME	G G G E K R S G S R I D
tr:F3YDH0:F3YDH0_DROME	G G G E K R S G S R I D
tr:F3YDH5:F3YDH5_DROME	G G G E K R S G S R I D
tr:Q8IH88:Q8IH88_DROME	G G G E K R S G S R I D
sp:P09446:HSP7A_CAEEL	G G G E K R S G S R I D
sp:P10592:HSP72_YEAST	G G G E K R S G S R I D

protein	whole protein ident	simil	domain-based ident	simil	contact-based ident	simil
PDB:3fzk:chainA:P11142	1.0	1.0	1.0	1.0	1.0	1.0
tr:E9PN89:E9PN89_HUMAN	0.48	0.48	0.93	0.93	1.0	1.0
tr:E7EP94:E7EP94_HUMAN	0.72	0.81	0.9	0.97	1.0	1.0
tr:Q96IS6:Q96IS6_HUMAN	0.91	0.91	1.0	1.0	1.0	1.0
tr:E9PKE3:E9PKE3_HUMAN	0.97	0.97	1.0	1.0	1.0	1.0
sp:P08107:HSP71_HUMAN	0.84	0.94	0.9	0.97	1.0	1.0
sp:P11142:HSP7C_HUMAN	1.0	1.0	1.0	1.0	1.0	1.0
tr:A8K5I0:A8K5I0_HUMAN	0.84	0.94	0.9	0.97	1.0	1.0
tr:M0R8M9:M0R8M9_RAT	0.99	1.0	0.98	0.99	1.0	1.0
tr:F1LZI1:F1LZI1_RAT	1.0	1.0	1.0	1.0	1.0	1.0
tr:M0RCB1:M0RCB1_RAT	0.98	0.99	0.99	1.0	1.0	1.0
tr:Q3TB63:Q3TB63_MOUSE	0.71	0.71	1.0	1.0	1.0	1.0
tr:Q3KQJ4:Q3KQJ4_MOUSE	0.87	0.87	1.0	1.0	1.0	1.0
tr:Q504P4:Q504P4_MOUSE	0.97	0.97	1.0	1.0	1.0	1.0
sp:P63017:HSP7C_MOUSE	1.0	1.0	1.0	1.0	1.0	1.0
tr:Q8I0E9:Q8I0E9_DROME	0.68	0.8	0.88	0.98	1.0	1.0
sp:O97125:HSP68_DROME	0.7	0.89	0.81	0.94	1.0	1.0
sp:P29843:HSP7A_DROME	0.8	0.93	0.88	0.98	1.0	1.0
tr:F3YDI6:F3YDI6_DROME	0.73	0.9	0.8	0.93	1.0	1.0
sp:P82910:HSP70_DROME	0.73	0.9	0.8	0.93	1.0	1.0
sp:P02825:HSP71_DROME	0.73	0.9	0.8	0.93	1.0	1.0
tr:C7LA76:C7LA76_DROME	0.73	0.9	0.79	0.93	1.0	1.0
sp:P29844:HSP7C_DROME	0.59	0.83	0.64	0.9	1.0	1.0
sp:P11147:HSP7D_DROME	0.84	0.95	0.9	0.98	1.0	1.0
tr:C7LA75:C7LA75_DROME	0.84	0.95	0.9	0.98	1.0	1.0
tr:F3YDH0:F3YDH0_DROME	0.59	0.83	0.64	0.9	1.0	1.0
tr:F3YDH5:F3YDH5_DROME	0.77	0.9	0.88	0.98	1.0	1.0
tr:Q8IH88:Q8IH88_DROME	0.75	0.88	0.88	0.98	1.0	1.0
sp:P09446:HSP7A_CAEEL	0.84	0.94	0.9	0.97	1.0	1.0
sp:P10592:HSP72_YEAST	0.75	0.92	0.85	0.96	1.0	1.0

Hsc70-1 (FBgn0001216) associated phenotypes

partially lethal - majority die, some die during pupal stage

(Information from FlyBase)

Hsp68 (FBgn0001230) associated phenotypes

chemical resistant, long lived, neuroanatomy defective, partially lethal - majority die, some die during pupal stage, starvation stress response defective

(Information from FlyBase)

Hsc70-1 (UniProt:P29843) annotation

Developmental stage: Heat shock cognate proteins are expressed constitutively during normal development.

(Information from UniProt)

Hsp70Aa (UniProt:P82910) annotation

Induction: Heat shock induces the synthesis of seven proteins at five otherwise inactive sites in the polytene chromosomes of fruit fly larvae. Two separate sites, producing two and three copies, respectively, code for the 70 kDa protein.
Miscellaneous: There are two copies of the gene coding for this protein at chromosome locus 87A7.

(Information from UniProt)

Hsp70Ab (UniProt:P02825) annotation

Induction: Heat shock induces the synthesis of seven proteins at five otherwise inactive sites in the polytene chromosomes of fruit fly larvae. Two separate sites, producing two and three copies, respectively, code for the 70 kDa protein.
Miscellaneous: There are two copies of the gene coding for this protein at chromosome locus 87A7.

(Information from UniProt)

Hsc70-3 (FBgn0001218) associated phenotypes
dominant, neuroanatomy defective, partially lethal - majority die

(Information from FlyBase)

Hsc70-3 (UniProt:P29844) annotation
Function: Probably plays a role in facilitating the assembly of multimeric protein complexes inside the ER.
Subcellular location: Endoplasmic reticulum lumen.

(Information from UniProt)

Hsc70-4 (UniProt:P11147) annotation
Subcellular location: Cytoplasm, perinuclear region. Nucleus. Note=Localized to a meshwork of cytoplasmic fibers around the nucleus. Translocates to the nucleus after thermal stress.
Developmental stage: Heat shock cognate proteins are expressed constitutively during normal development.

(Information from UniProt)

hsp-1 (WBGene00002005) associated phenotypes
lethal, sterile

(Information from WormBase)
