# Supplementary material for: Drug repurposing for aging research using model organisms
Source: Aging Cell. 2017 Jun 16;16(5):1006–15. doi: 10.1111/acel.12626 (PMC5595691; doi:10.1111/acel.12626)
Supplement: Supplementary file 7 — Data S1 Zip‐Archive of all report cards. [file ACEL-16-1006-s007.zip › RC_3JZ.pdf]

3JZ

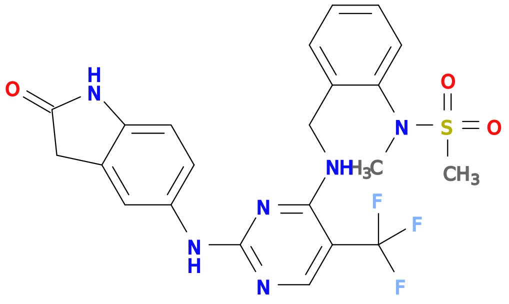

Database identifiers

ChEMBLCompound	CHEMBL472212
eMolecules	31507636

Ranking

	Rank	Score
Drosophila	641/697	0.119
C. elegans	544/591	0.034

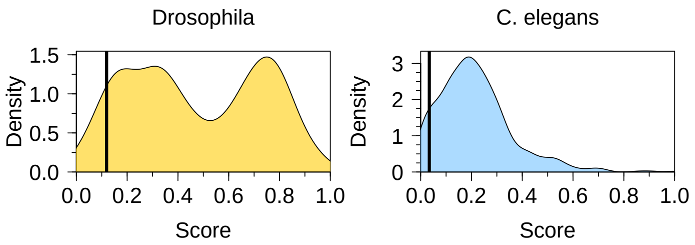

	Ageing implication	Domain conservation	Binding site conservation	Binding affinity	Bioavailability	Lipinski	Promiscuity	Purchasability	Drug approval	**Total**
Drosophila	0.203	0.83	0.845	0.936	(0.9)	-0.1	-0.0	0.1	0.0	0.119
C. elegans	0.203	0.899	0.849	0.936	0.238	-0.1	-0.0	0.1	0.0	0.034

Names

No synonyms found

Roles

ChEBI entry None has no roles

Status

Approved drug (according to ChEMBL)	No
Number of Rule of 5 violations	2
Binding affinity to original target in log units (RF-Score prediction)	7.68
Burns *C. elegans* bioavailability prediction	-2.77

Compound Target Characteristics

Protein-tyrosine kinase 2-beta

Best gene implication in ageing for this target family came from gene Q14289 via mapping the annotation from Ensembl ENSG00000120899 via mapping the annotation from EntrezGene 2185 via mapping the annotation from GenAgeHuman 0165 annotated in GenAge release 17.

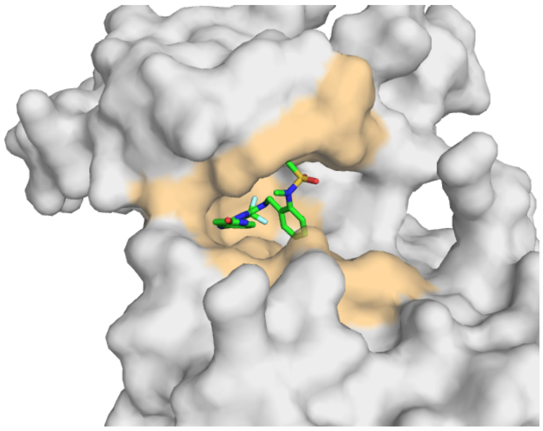

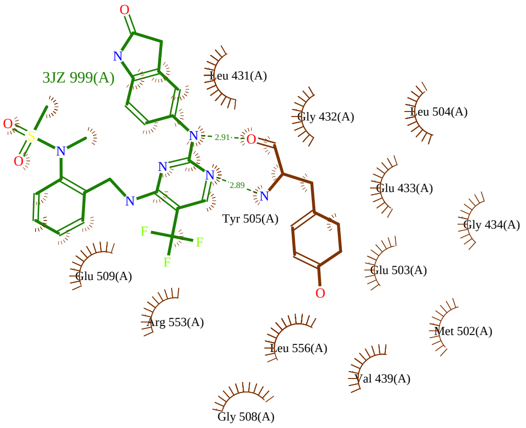

protein	amino acids contacts (binding site)
PDB:3fzr:chainA:Q14289	L G E G V A V M E L Y G E R L D
tr:E9PBI4:E9PBI4_HUMAN	L G E G V A V M E L Y G E R L D
sp:Q14289:FAK2_HUMAN	L G E G V A V M E L Y G E R L D
sp:P70600:FAK2_RAT	L G E G V A V M E L Y G E R L D
tr:E9Q2A6:E9Q2A6_MOUSE	L G E G V A V M E L Y G E R L D
sp:Q9QVP9:FAK2_MOUSE	L G E G V A V M E L Y G E R L D
tr:Q3UDE9:Q3UDE9_MOUSE	L G E G V A V M E L Y G E R L D
tr:K7QD41:K7QD41_MOUSE	L G E G V A V M E L Y G E R L D
tr:Q2TGI0:Q2TGI0_DROME	I G V G V A I M E L A G E R L D
tr:Q9U5Y2:Q9U5Y2_DROME	I G V G V A I M E L A G E R L D
tr:E1JGM8:E1JGM8_DROME	I G V G V A I M E L A G E R L D
tr:B8A418:B8A418_DROME	I G V G V A I M E L A G E R L D
tr:Q5BIG9:Q5BIG9_DROME	I G V G V A I M E L A G E R L D
tr:Q0E917:Q0E917_DROME	I G V G V A I M E L A G E R L D
tr:Q9U531:Q9U531_DROME	I G V G V A I M E L A G E R L D
tr:Q9U472:Q9U472_DROME	I G V G V A I M E L A G E R L D
tr:B7YZL9:B7YZL9_DROME	I G V G V A I M E L A G E R L D
tr:Q8T879:Q8T879_CAEEL	I G G G V A I L E L A G E R L D
tr:Q95YD4:Q95YD4_CAEEL	I G G G V A I L E L A G E R L D

protein	whole protein ident	simil	domain-based ident	simil	contact-based ident	simil
PDB:3fzr:chainA:Q14289	1.0	1.0	1.0	1.0	1.0	1.0
tr:E9PBI4:E9PBI4_HUMAN	0.54	0.56	1.0	1.0	1.0	1.0
sp:Q14289:FAK2_HUMAN	1.0	1.0	1.0	1.0	1.0	1.0
sp:P70600:FAK2_RAT	0.96	0.99	0.98	0.99	1.0	1.0
tr:E9Q2A6:E9Q2A6_MOUSE	0.91	0.95	0.98	0.99	1.0	1.0
sp:Q9QVP9:FAK2_MOUSE	0.95	0.99	0.98	0.99	1.0	1.0
tr:Q3UDE9:Q3UDE9_MOUSE	0.95	0.98	0.98	0.99	1.0	1.0
tr:K7QD41:K7QD41_MOUSE	0.95	0.99	0.98	0.99	1.0	1.0
tr:Q2TGI0:Q2TGI0_DROME	0.14	0.22	0.51	0.76	0.75	0.84
tr:Q9U5Y2:Q9U5Y2_DROME	0.25	0.59	0.51	0.76	0.75	0.84
tr:E1JGM8:E1JGM8_DROME	0.25	0.59	0.51	0.76	0.75	0.84
tr:B8A418:B8A418_DROME	0.25	0.59	0.51	0.76	0.75	0.84
tr:Q5BIG9:Q5BIG9_DROME	0.25	0.59	0.5	0.76	0.75	0.84
tr:Q0E917:Q0E917_DROME	0.25	0.59	0.51	0.76	0.75	0.84
tr:Q9U531:Q9U531_DROME	0.25	0.59	0.51	0.76	0.75	0.84
tr:Q9U472:Q9U472_DROME	0.25	0.6	0.51	0.76	0.75	0.84
tr:B7YZL9:B7YZL9_DROME	0.2	0.48	0.51	0.76	0.75	0.84
tr:Q8T879:Q8T879_CAEEL	0.2	0.56	0.46	0.82	0.69	0.85
tr:Q95YD4:Q95YD4_CAEEL	0.21	0.6	0.46	0.82	0.69	0.85

Fak (FBgn0020440) associated phenotypes

neuroanatomy defective, neurophysiology defective

(Information from FlyBase)
