# Supplementary material for: Drug repurposing for aging research using model organisms
Source: Aging Cell. 2017 Jun 16;16(5):1006–15. doi: 10.1111/acel.12626 (PMC5595691; doi:10.1111/acel.12626)
Supplement: Supplementary file 7 — Data S1 Zip‐Archive of all report cards. [file ACEL-16-1006-s007.zip › RC_3O0.pdf]

3O0

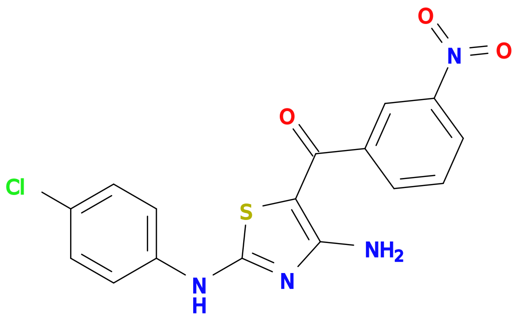

Database identifiers

ChEMBLCompound	CHEMBL1614763
ZINC	ZINC01398640
eMolecules	4909014

Ranking

	Rank	Score
Drosophila	211/697	0.689
C. elegans	144/591	0.287

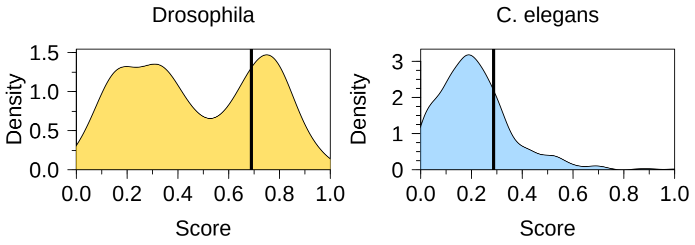

	Ageing implication	Domain conservation	Binding site conservation	Binding affinity	Bioavailability	Lipinski	Promiscuity	Purchasability	Drug approval	**Total**
Drosophila	1.0	0.967	0.928	0.791	(0.9)	-0.05	-0.0	0.1	0.0	0.689
C. elegans	1.0	0.965	0.974	0.791	0.319	-0.05	-0.0	0.1	0.0	0.287

Names

No synonyms found

Roles

ChEBI entry None has no roles

Status

Approved drug (according to ChEMBL)	No
Number of Rule of 5 violations	1
Binding affinity to original target in log units (RF-Score prediction)	6.33
Burns *C. elegans* bioavailability prediction	0.09

Compound Target Characteristics

Cyclin-dependent-like kinase 5

Best gene implication in ageing for this target family came from gene P48609 annotated in UniProt release 2014_02. Annotation GO 8340 (determination of adult lifespan) was Inferred from Mutant Phenotype

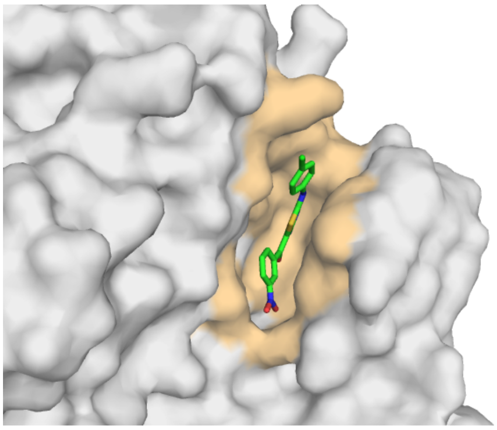

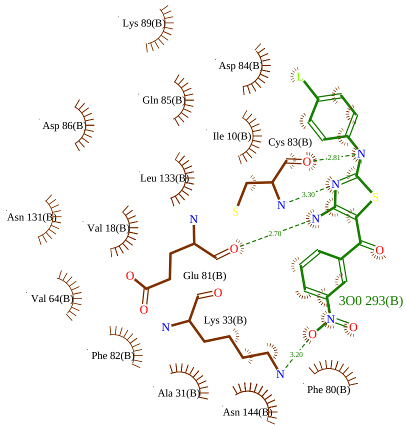

protein	amino acids contacts (binding site)
PDB:3o0g:chainB:Q00535	I V A K V F E F C D Q D K N L N
sp:Q00535:CDK5_HUMAN	I V A K V F E F C D Q D K N L D
sp:Q03114:CDK5_RAT	I V A K V F E F C D Q D K N L D
sp:P49615:CDK5_MOUSE	I V A K V F E F C D Q D K N L D
tr:Q543F6:Q543F6_MOUSE	I V A K V F E F C D Q D K N L D
sp:P48609:CDK5_DROME	I V A K V F E H C D Q D K N L D
tr:G5ECH7:G5ECH7_CAEEL	I V A K V F E Y C D Q D K N L D
sp:P17157:PHO85_YEAST	L V A K V F E F M D N D K N L D

	whole protein		domain-based		contact-based	
protein	ident	simil	ident	simil	ident	simil
PDB:3o0g:chainB:Q00535	1.0	1.0	1.0	1.0	1.0	1.0
sp:Q00535:CDK5_HUMAN	1.0	1.0	1.0	1.0	0.94	0.99
sp:Q03114:CDK5_RAT	0.99	1.0	0.99	1.0	0.94	0.99
sp:P49615:CDK5_MOUSE	1.0	1.0	1.0	1.0	0.94	0.99
tr:Q543F6:Q543F6_MOUSE	1.0	1.0	1.0	1.0	0.94	0.99
sp:P48609:CDK5_DROME	0.78	0.93	0.79	0.94	0.88	0.93
tr:G5ECH7:G5ECH7_CAEEL	0.74	0.93	0.75	0.93	0.88	0.97
sp:P17157:PHO85_YEAST	0.54	0.82	0.56	0.83	0.75	0.84

Cdk5 (FBgn0013762) associated phenotypes
neuroanatomy defective, short lived

(Information from FlyBase)

Cdk5 (UniProt:P48609) annotation
Function: Probably involved in the control of the cell cycle. Interacts with D1 and D3-type G1 cyclins. Possible regulator of neuronal differentiation and/or development (By similarity).
Tissue specificity: Abundantly expressed in all adult tissues. Lower levels found in larvae and early embryos. Barely detectable in late embryos.

(Information from UniProt)

cdk-5 (WBGene00000407) associated phenotypes
aldicarb resistant

(Information from WormBase)

cdk-5 (UniProt:G5ECH7) annotation

Function: Proline-directed serine/threonine-protein kinase which, in several motor neurons, promotes the polarized trafficking of synaptic vesicles and dense-core vesicles (DCV). In the ventral nerve cord, phosphorylates lin-10 and thereby prevents lin-10- mediated anterograde trafficking of the glutamate receptor glr-1 (PubMed:17671168, PubMed:21609829). Involved in the inhibition of glr-1 trafficking in hypoxic conditions (PubMed:22252129). In DA motor neurons but not in DB motor neurons, regulates axonal transport of synaptic vesicle precursors by inhibiting dynein-mediated retrograde transport (PubMed:20510931). Regulates the trafficking of dense-core vesicles in DA and DB motor neurons by promoting anterograde trafficking to the axon and preventing dynein-dependent trafficking to the dendrite (PubMed:22699897). May regulate these processes in association with cdka-1/p35 (PubMed:17671168, PubMed:20510931). Activity may be regulated by cyy-1 (PubMed:20510931). Involved in synapse formation during DD motor neuron remodeling by regulating transport of disassembled synaptic material to the new synaptic sites probably by activating the motor protein unc-104/kinesin-3 (PubMed:21609829). Regulates microtubule polarity in the dendrite of DB motor neurons (PubMed:22699897). (PubMed:17671168, PubMed:20510931, PubMed:21609829, PubMed:22252129, PubMed:22699897).

Cofactor: Mg(2+) Evidence=(PubMed:20510931); Note=Binds 2 Mg(2+) ions. (UniProtKB:P24941);

Subunit: Heterodimer composed of a catalytic subunit cdk-5 and a regulatory subunit cdka-1. Interaction with cdka-1 is required for cdk-5 activation. (PubMed:22699897).

Subcellular location: Cytoplasm (PubMed:20510931). Cell projection, dendrite (PubMed:20510931). Note=Localizes predominantly to presynaptic sites and in dendrites as faint puncta. (PubMed:20510931).

Disruption phenotype: Several glr-1-dependent behaviors are affected including an absence of backward locomotion after nose- touching stimuli and a reduction in reverse locomotion (PubMed:17671168). In L4 mutants, incomplete elimination of ventral rab-3-positive synaptic vesicles associated with a delay in the formation of dorsal rab-3-positive synaptic vesicles in DD motor neurons. Normal formation of ventral synapses in DD motor neurons at the L1 stage (PubMed:21609829). In addition, mutants have an increase in anterograde dense-core vesicle trafficking and in the number of plus-end-out microtubules in DB motor neuron dendrites (PubMed:22699897). Reduced sensitivity to the acetylcholine esterase inhibitor aldicarb (PubMed:22699897). (PubMed:17671168, PubMed:21609829, PubMed:22699897).

(Information from UniProt)
