# Supplementary material for: Drug repurposing for aging research using model organisms
Source: Aging Cell. 2017 Jun 16;16(5):1006–15. doi: 10.1111/acel.12626 (PMC5595691; doi:10.1111/acel.12626)
Supplement: Supplementary file 7 — Data S1 Zip‐Archive of all report cards. [file ACEL-16-1006-s007.zip › RC_3RD.pdf]

3RD

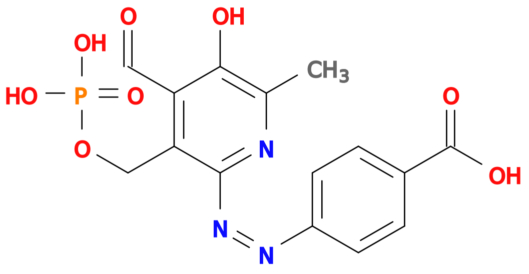

Database identifiers
ChEMBLCompound CHEMBL119235

Ranking

	Rank	Score
Drosophila	533/697	0.223
C. elegans	560/591	0.019

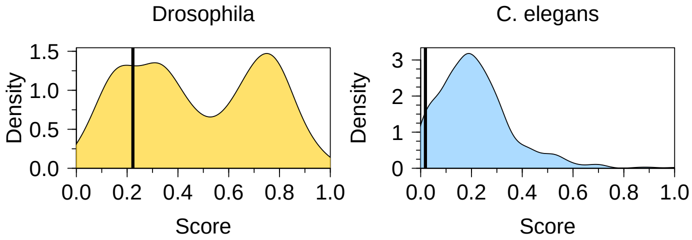

	Ageing implication	Domain conservation	Binding site conservation	Binding affinity	Bioavailability	Lipinski	Promiscuity	Purchasability	Drug approval	**Total**
Drosophila	0.81	0.97	1.0	0.386	(0.9)	-0.05	-0.0	0.0	0.0	0.223
C. elegans	0.81	0.971	1.0	0.386	0.228	-0.05	-0.0	0.0	0.0	0.019

Names

No synonyms found

Roles

ChEBI entry None has no roles

Status

Approved drug (according to ChEMBL)	No
Number of Rule of 5 violations	1
Binding affinity to original target in log units (RF-Score prediction)	4.54
Burns *C. elegans* bioavailability prediction	-3.49

Compound Target Characteristics

14-3-3 protein zeta/delta

Best gene implication in ageing for this target family came from gene P41932 via mapping the annotation from WormBase WBGene00003920 annotated in WormBase 2014-03-11. Annotation GO 8340 (determination of adult lifespan) was Inferred from Mutant Phenotype

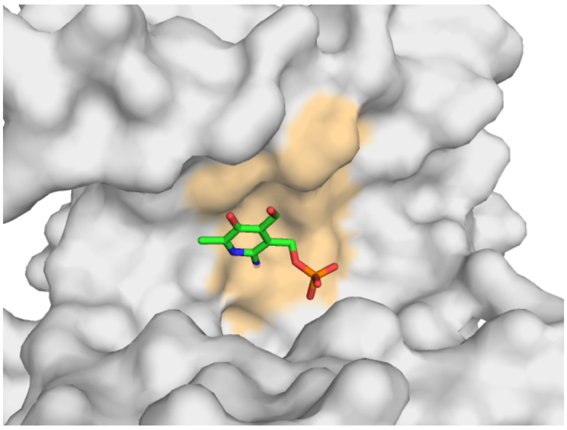

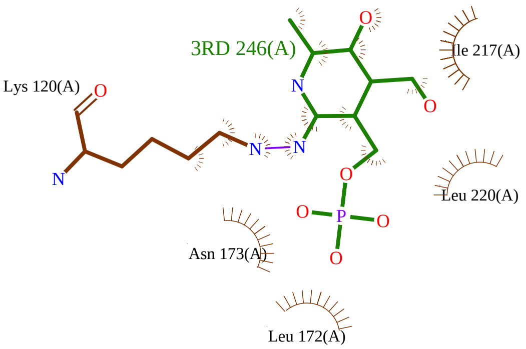

protein	amino acids contacts (binding site)
PDB:3rdh:chainA:P63104	K G L N I L
tr:H0YB80:H0YB80_HUMAN	K G L N I L
tr:B0AZS6:B0AZS6_HUMAN	K G L N I L
tr:E7EX29:E7EX29_HUMAN	K G L N I L
sp:P63104:1433Z_HUMAN	K G L N I L
tr:D0PNI1:D0PNI1_HUMAN	K G L N I L
sp:P63102:1433Z_RAT	K G L N I L
sp:P63101:1433Z_MOUSE	K G L N I L
sp:P29310:1433Z_DROME	K G L N I L
sp:P41932:14331_CAEEL	K G L N I L
sp:Q20655:14332_CAEEL	K G L N I L

	whole protein		domain-based		contact-based	
protein	ident	simil	ident	simil	ident	simil
PDB:3rdh:chainA:P63104	0.99	0.99	1.0	1.0	1.0	1.0
tr:H0YB80:H0YB80_HUMAN	0.53	0.53	0.5	0.5	1.0	1.0
tr:B0AZS6:B0AZS6_HUMAN	0.69	0.69	0.67	0.67	1.0	1.0
tr:E7EX29:E7EX29_HUMAN	0.94	0.94	0.97	0.97	1.0	1.0
sp:P63104:1433Z_HUMAN	1.0	1.0	1.0	1.0	1.0	1.0
tr:D0PNI1:D0PNI1_HUMAN	1.0	1.0	1.0	1.0	1.0	1.0
sp:P63102:1433Z_RAT	1.0	1.0	1.0	1.0	1.0	1.0
sp:P63101:1433Z_MOUSE	1.0	1.0	1.0	1.0	1.0	1.0
sp:P29310:1433Z_DROME	0.8	0.94	0.82	0.95	1.0	1.0
sp:P41932:14331_CAEEL	0.76	0.91	0.8	0.94	1.0	1.0
sp:Q20655:14332_CAEEL	0.79	0.92	0.83	0.95	1.0	1.0

14-3-3zeta (FBgn0004907) associated phenotypes

learning defective, maternal effect, memory defective, mitotic cell cycle defective, neurophysiology defective

(Information from FlyBase)

14-3-3zeta (UniProt:P29310) annotation

Function: Required in Raf-dependent cell proliferation and photoreceptor differentiation during eye development. Acts upstream of Raf and downstream of Ras, and is essential for viability. Acts as a negative regulator by decreasing its voltage sensitivity. Inhibits yki activity by restricting its nuclear localization. (PubMed:10230800, PubMed:19900439).

Subunit: Homodimer; homodimerization is not essential for modulating the activity of Slo. Inter-

acts with phosphorylated Slob; the interaction with Slob mediates an indirect interaction with Slo. Interacts with phosphorylated yki. (PubMed:10230800, PubMed:18256197, PubMed:19900439).
Subcellular location: Cytoplasm ECO:0000250.
Tissue specificity: Predominantly expressed in the ventral nerve cord of the embryo, and in the neural tissues of the head. Also found in the region posterior to the morphogenetic furrow of the eye imaginal disk where cells differentiate as photoreceptors.
Developmental stage: Expressed throughout all stages of embryonic and larval development.

(Information from UniProt)

par-5 (WBGene00003920) associated phenotypes

P granule localization defective, P0 spindle position defective early emb, antibody staining reduced, aster defective early emb, asymmetric cell division defective early emb, cell cleavage variant emb, embryonic polarity variant, gut granule biogenesis reduced, hermaphrodite fertility reduced, maternal effect lethal emb, maternal effect sterile, no Intestine, pharyngeal development variant, pronuclei meet centrally early emb, protein expression reduced, protein subcellular localization variant, pseudocleavage exaggerated early emb, relative cell cycle timing defective early emb, spindle orientation defective early emb, spindle orientation variant AB or P1 early emb, synchronous second division early emb

(Information from WormBase)

par-5 (UniProt:P41932) annotation

Subunit: Interacts with daf-16 and sir-2.1. (PubMed:16777605).
Subcellular location: Cytoplasm ECO:0000269—PubMed:16777605. Nucleus (PubMed:16777605).

(Information from UniProt)

ftt-2 (WBGene00001502) associated phenotypes

lethal, sterile

(Information from WormBase)

ftt-2 (UniProt:Q20655) annotation

Function: Required for extension of life-span by sir-2.1 (PubMed:16777605). Promotes nuclear export of yap-1 (PubMed:23396260). (PubMed:16777605, PubMed:23396260).
Subunit: Interacts with daf-16 and sir-2.1. (PubMed:16777605).
Subcellular location: Cytoplasm ECO:0000269—PubMed:16777605. Nucleus (PubMed:16777605).

(Information from UniProt)
