# Supplementary material for: Drug repurposing for aging research using model organisms
Source: Aging Cell. 2017 Jun 16;16(5):1006–15. doi: 10.1111/acel.12626 (PMC5595691; doi:10.1111/acel.12626)
Supplement: Supplementary file 7 — Data S1 Zip‐Archive of all report cards. [file ACEL-16-1006-s007.zip › RC_47X.pdf]

47X

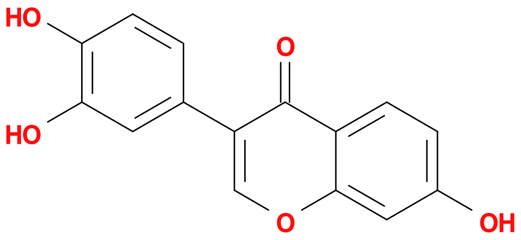

Database identifiers

ChEMBLCompound	CHEMBL13486
CHEBI	50399
ZINC	ZINC00391976
eMolecules	532752

Ranking

	Rank	Score
Drosophila	NA	NA
C. elegans	21/591	0.532

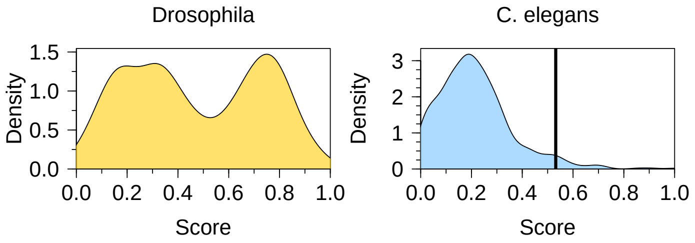

	Ageing implication	Domain conservation	Binding site conservation	Binding affinity	Bioavailability	Lipinski	Promiscuity	Purchasability	Drug approval	**Total**
Drosophila	NA	NA	NA	NA	NA	NA	NA	NA	NA	NA
C. elegans	0.81	0.853	0.929	0.842	0.8	0.0	-0.0	0.1	0.0	0.532

Names

- **3',4',7-trihydroxyisoflavone**
- 3'-hydroxydaidzein
- 3-(3,4-dihydroxyphenyl)-7-hydroxy-4H-1-benzopyran-4-one

Roles

metabolite, antineoplastic agent, EC 1.3.1.22 [3-oxo-5alpha-steroid 4-dehydrogenase (NADP(+))] inhibitor

Status

Approved drug (according to ChEMBL)	No
Number of Rule of 5 violations	0
Binding affinity to original target in log units (RF-Score prediction)	6.67
Burns *C. elegans* bioavailability prediction	6.47

Compound Target Characteristics

Macrophage migration inhibitory factor

Best gene implication in ageing for this target family came from gene P34884 annotated in UniProt release 2014_02. Annotation GO subterm of 7568 (aging) was Inferred from Mutant Phenotype

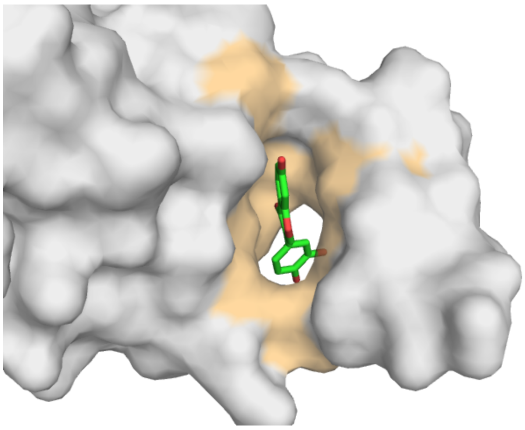

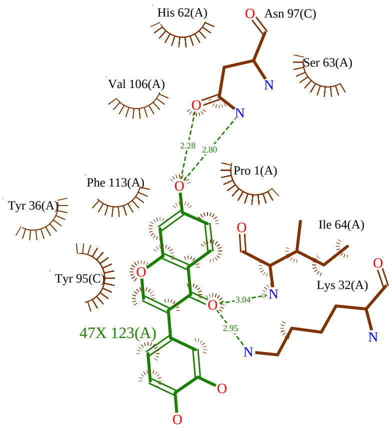

protein	amino acids contacts (binding site)
PDB:3l5r:chainA:P14174	P K Y H S I V F
sp:P14174:MIF_HUMAN	P K Y H S I V F
tr:I4AY87:I4AY87_HUMAN	P K Y H S I V F
sp:P30904:MIF_RAT	P K Y H S I V F
tr:D3ZE63:D3ZE63_RAT	P K Y H S I V F
tr:D4A3P7:D4A3P7_RAT	P K Y H S I V F
sp:P34884:MIF_MOUSE	P K Y H S I V F
tr:Q545F0:Q545F0_MOUSE	P K Y H S I V F
tr:Q9U228:Q9U228_CAEEL	P K Y K S I I F

	whole protein		domain-based		contact-based	
protein	ident	simil	ident	simil	ident	simil
PDB:3l5r:chainA:P14174	1.0	1.0	1.0	1.0	1.0	1.0
sp:P14174:MIF_HUMAN	1.0	1.0	1.0	1.0	1.0	1.0
tr:I4AY87:I4AY87_HUMAN	1.0	1.0	1.0	1.0	1.0	1.0
sp:P30904:MIF_RAT	0.9	0.98	0.9	0.98	1.0	1.0
tr:D3ZE63:D3ZE63_RAT	0.86	0.96	0.86	0.96	1.0	1.0
tr:D4A3P7:D4A3P7_RAT	0.83	0.96	0.83	0.96	1.0	1.0
sp:P34884:MIF_MOUSE	0.9	0.97	0.89	0.97	1.0	1.0
tr:Q545F0:Q545F0_MOUSE	0.9	0.97	0.89	0.97	1.0	1.0
tr:Q9U228:Q9U228_CAEEL	0.33	0.78	0.33	0.78	0.75	0.93
