# Supplementary material for: Drug repurposing for aging research using model organisms
Source: Aging Cell. 2017 Jun 16;16(5):1006–15. doi: 10.1111/acel.12626 (PMC5595691; doi:10.1111/acel.12626)
Supplement: Supplementary file 7 — Data S1 Zip‐Archive of all report cards. [file ACEL-16-1006-s007.zip › RC_530.pdf]

530

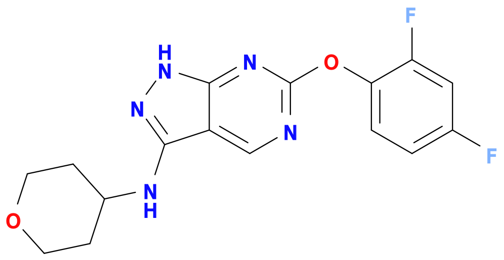

Database identifiers
ChEMBLCompound CHEMBL1230350

Ranking

	Rank	Score
Drosophila	154/697	0.745
C. elegans	336/591	0.176

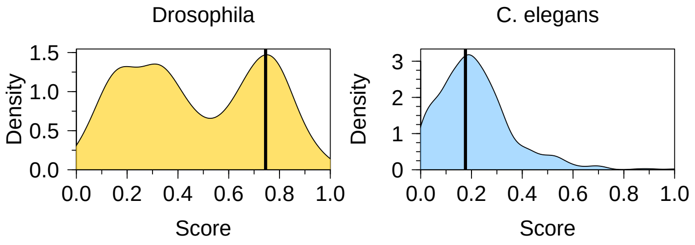

	Ageing implication	Domain conservation	Binding site conservation	Binding affinity	Bioavailability	Lipinski	Promiscuity	Purchasability	Drug approval	**Total**
Drosophila	1.0	0.959	0.986	0.877	(0.9)	0.0	-0.0	0.0	0.0	0.745
C. elegans	1.0	0.96	0.87	0.877	0.242	0.0	-0.0	0.0	0.0	0.176

Names

No synonyms found

Roles

ChEBI entry None has no roles

Status

Approved drug (according to ChEMBL)	No
Number of Rule of 5 violations	0
Binding affinity to original target in log units (RF-Score prediction)	6.96
Burns *C. elegans* bioavailability prediction	-2.53

Compound Target Characteristics

Mitogen-activated protein kinase 14

Best gene implication in ageing for this target family came from gene O62618 annotated in UniProt release 2014_02. Annotation GO 8340 (determination of adult lifespan) was Inferred from Mutant Phenotype

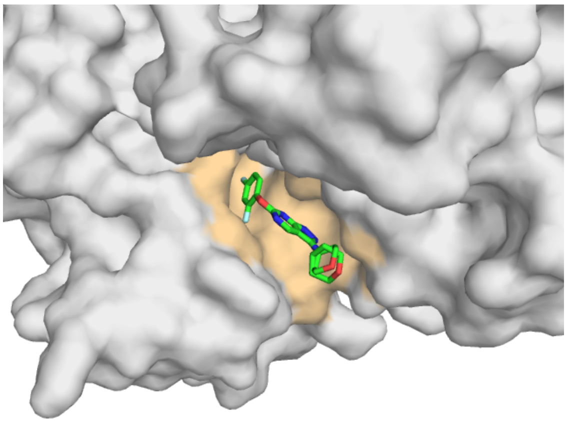

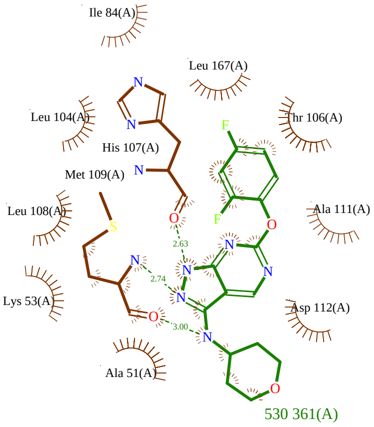

protein	amino acids contacts (binding site)														
PDB:3fmn:chainA:Q16539	V	A	V	K	I	L	L	V	T	H	L	M	A	D	L
tr:L7RSM2:L7RSM2_HUMAN	V	A	V	K	I	L	L	V	T	H	L	M	A	D	L
sp:Q16539:MK14_HUMAN	V	A	V	K	I	L	L	V	T	H	L	M	A	D	L
tr:G3V617:G3V617_RAT	V	A	V	K	I	L	L	V	T	H	L	M	A	D	L
tr:Q56A33:Q56A33_RAT	V	A	V	K	I	L	L	V	T	H	L	M	A	D	L
tr:B2KF34:B2KF34_MOUSE	V	A	V	K	I	L	L	V	T	H	L	M	A	D	L
tr:B2KF35:B2KF35_MOUSE	V	A	V	K	I	L	L	V	T	H	L	M	A	D	L
sp:P47811:MK14_MOUSE	V	A	V	K	I	L	L	V	T	H	L	M	A	D	L
tr:Q5U421:Q5U421_MOUSE	V	A	V	K	I	L	L	V	T	H	L	M	A	D	L
sp:P83100:MK14C_DROME	V	A	M	K	I	L	L	V	T	H	L	M	A	D	L
sp:O62618:MK14A_DROME	V	A	I	K	I	L	L	V	T	H	L	M	A	D	L
tr:E1JIV6:E1JIV6_DROME	V	A	I	K	I	L	L	V	T	H	L	M	A	D	L
sp:Q17446:PMK1_CAEEL	V	A	I	K	I	L	F	V	S	M	L	M	A	D	L
sp:P32485:HOG1_YEAST	V	A	I	K	I	L	F	V	T	E	L	Q	T	D	C

protein	whole protein ident	whole protein simil	domain-based ident	domain-based simil	contact-based ident	contact-based simil
PDB:3fmn:chainA:Q16539	1.0	1.0	1.0	1.0	1.0	1.0
tr:L7RSM2:L7RSM2_HUMAN	0.96	0.99	0.95	0.99	1.0	1.0
sp:Q16539:MK14_HUMAN	1.0	1.0	1.0	1.0	1.0	1.0
tr:G3V617:G3V617_RAT	0.99	1.0	0.99	1.0	1.0	1.0
tr:Q56A33:Q56A33_RAT	0.95	0.99	0.95	0.99	1.0	1.0
tr:B2KF34:B2KF34_MOUSE	0.56	0.56	0.62	0.62	1.0	1.0
tr:B2KF35:B2KF35_MOUSE	0.7	0.71	0.8	0.8	1.0	1.0
sp:P47811:MK14_MOUSE	0.99	1.0	0.99	1.0	1.0	1.0
tr:Q5U421:Q5U421_MOUSE	0.96	0.99	0.95	0.99	1.0	1.0
sp:P83100:MK14C_DROME	0.44	0.79	0.46	0.83	0.93	0.99
sp:O62618:MK14A_DROME	0.67	0.88	0.74	0.91	0.93	0.98
tr:E1JIV6:E1JIV6_DROME	0.67	0.88	0.74	0.91	0.93	0.98
sp:Q17446:PMK1_CAEEL	0.62	0.85	0.71	0.92	0.73	0.87
sp:P32485:HOG1_YEAST	0.41	0.68	0.54	0.85	0.6	0.7

p38c (UniProt:P83100) annotation

Function: Kinase involved in a signal transduction pathway.

Cofactor:Mg(2+)

Ptm: The phosphorylation on Thr-177 activates the enzyme (By similarity). A conserved Tyr, which must also be phosphorylated to activate the enzyme in closely related sequences, is replaced

by His-179 in this sequence.

(Information from UniProt)

Mpk2 (FBgn0015765) associated phenotypes

heat stress response defective, immune response defective, non-enhancer of variegation, non-suppressor of variegation, nutrition conditional, osmotic stress response defective, oxidative stress response defective, partially lethal - majority die, reduced, short lived, starvation stress response defective, stress response defective

(Information from FlyBase)

Mpk2 (UniProt:O62618) annotation

Function: Kinase involved in a signal transduction pathway. May down-regulate insect immunity gene expression after prolonged infection. (PubMed:9417090, PubMed:9584193).
Cofactor:Mg(2+)
Enzyme regulation: Activated by threonine and tyrosine phosphorylation by Mkk3 in response to environmental stress. (PubMed:9417090, PubMed:9584193).
Subcellular location: Nucleus (PubMed:9417090).
Developmental stage: Expressed both maternally and zygotically. Levels are highest at the preblastoderm stage but low levels are present throughout development. (PubMed:9584193).
Domain: The TXY motif contains the threonine and tyrosine residues whose phosphorylation activates the MAP kinases.
Ptm: Dually phosphorylated on Thr-184 and Tyr-186, which activates the enzyme. (PubMed:9417090).

(Information from UniProt)

pmk-1 (WBGene00004055) associated phenotypes

cell stress response variant, drug induced gene expression variant, gene expression level reduced, lethal, life span variant, organism oxidative stress response hypersensitive, pathogen induced gene expression variant, pathogen susceptibility increased, sterile

(Information from WormBase)

pmk-1 (UniProt:Q17446) annotation

Function: Responds to activation by environmental stress and pro- inflammatory cytokines by phosphorylating downstream targets. Functions downstream of the MAPKK sek-1 and the MAPKKK nsy-1 as the MAP kinase required for pathogen resistance. (PubMed:11703092, PubMed:12142542).
Cofactor: Mg(2+) Evidence=(PubMed:11703092);
Enzyme regulation: Activated by phosphorylation on threonine and tyrosine. Inhibited by pyridinyl-imidazole related compounds. (PubMed:11703092).
Domain: The TXY motif contains the threonine and tyrosine residues whose phosphorylation activates the MAP kinases.
Ptm: Dually phosphorylated on Thr-191 and Tyr-193, which activates the enzyme.

(Information from UniProt)
