# Supplementary material for: Drug repurposing for aging research using model organisms
Source: Aging Cell. 2017 Jun 16;16(5):1006–15. doi: 10.1111/acel.12626 (PMC5595691; doi:10.1111/acel.12626)
Supplement: Supplementary file 9 — Data S3 Supplemental Text: PDBbind mimicking files for RF‐Score & Bioavailability Prediction assessment. [file ACEL-16-1006-s009.pdf]

Supplement: Drug repurposing for ageing research using model organisms

Matthias Ziehm[1,2,$], Satwant Kaur[1], Dobril K. Ivanov[1], Pedro J. Ballester[1,%], David Marcus[1], Linda Partridge[2,3], Janet M. Thornton[1]

1 European Molecular Biology Laboratory, European Bioinformatics Institute (EMBL-EBI), The Genome Campus, Hinxton, Cambridge CB10 1SD, UK;
2 Institute of Healthy Ageing, Department of Genetics, Evolution and Environment, University College London, Gower Street, London WC1E 6BT, UK
3 Max Planck Institute for Biology of Ageing, Joseph-Stelzmann-Str. 9b, 50931 Cologne, Germany
$ current address: Max Delbrück Center for Molecular Medicine, Robert-Rössle-Str. 10, 13092 Berlin, Germany
% current address: current address: Cancer Research Center of Marseille, INSERM U1068, F-13009 Marseille, France; Institut Paoli-Calmettes, F-13009 Marseille, France; Aix-Marseille Université, F-13284 Marseille, France; and CNRS UMR7258, F-13009 Marseille, France

PDBbind mimicking files for RF-Score

We used the machine learning-based binding affinity prediction method RF-Score v.2 (Ballester *et al.* 2014) shown to have high prediction accuracy. This machine-learning scoring function was trained on the files from the PDBbind database (Wang *et al.* 2004), and requires structure-ligand complexes in that format. Since most complexes of interest are not in PDBbind and there was no complete conversion script available, we developed a small pipeline for converting PDB files containing a protein with bound ligand into a PDBbind like format, mimicking the original PDBbind as closely as possible.

PDBbind mimicking procedure

For each PDB code to be converted we downloaded the PDB biounit file, if available, otherwise the standard file is used. The first occurrence of the ligand of interest is extracted and all corresponding HETATM lines of the PDB written to a separate PDB file. This is converted into mol2 format and protonated using OpenBabel (O'Boyle *et al.* 2011). All SEQRES, SSBOND, TER and ATOM lines of the PDB files are extracted into a protein PDB file, which is protonated by the CCP4 programme hgen (CCP4 v.4-6.4.0)(Winn *et al.* 2011).

A new protein files, is prefixed by three lines:

- “HEADER " + PDB code + "_PROTEIN"
- "COMPND " + PDB code + "_PROTEIN”
- "REMARK GENERATED BY MZiehm on “ + date and time

This is followed by the content of the hgen output PDB file, with few naming changes necessary to numbered atoms, for example:

- If the atom is called H1 or H1A but not followed by H2 or H2A, respectively, then omit "1"
- else if the atom is called H2G2, it is changed to 2HG2
- else if the atom is called H2A, it is changed to HA2

Then all HETATM of the original PDB file which were not extracted into the ligand file are added with renumbered atom numbers and the resulting PDB files send through XScore v1.2.1 (Wang *et al.* 2002) with option “-fixpdb”. Finally, the resulting “fixed” protein PDB file is used in combination with the ligand mol2 file to create the pocket files using “xscore -preppocket”.

Evaluation

In order to evaluate the impact of the differences of our PDBbind mimicking files compared to the original PDBbind format, we created PDBbind mimicking files for the 92 complexes of the PDBbind database which overlap with our list of complexes of interest. We then predicted the binding affinity for the PDBbind files of the 92 complexes and our PDBbind mimicking files for the same complexes and compared both results with the measured binding affinities provided by PDBbind. The results shown in the figure below demonstrates that our PDBbind mimicking files production pipeline works well and RF-Score produces high quality predictions for these files.

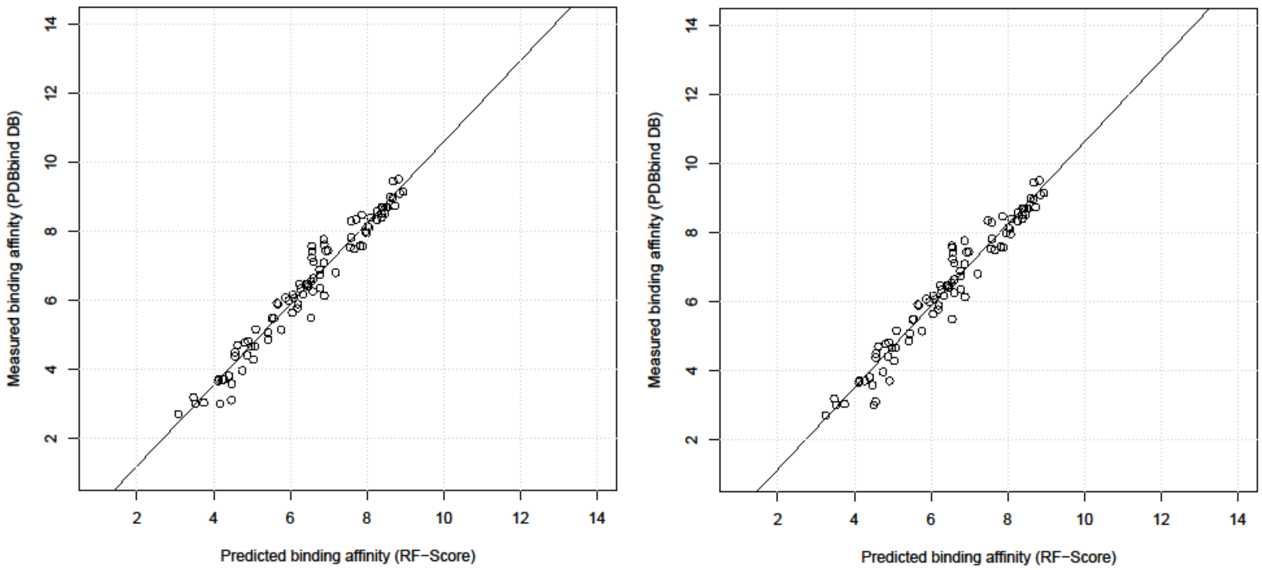

(A) On the original PDBbind files of these 92 complexes RF-Score predictions correlated extremely well with the measured binding affinities reported in PDBbind (Pearson r=0.978). (B) Nearly identical correlation of the RF-Score predictions based on our PDBbind mimicking files with the measured binding affinities from PDBbind (Pearson r=0.974).

Bioavailability Prediction assessment

Here we evaluated the only published bioavailability predictor for *C. elegans* (Burns *et al.* 2010), for its predictive performance. This was particularly important to establish its usefulness, appropriate weighting and transformation of the prediction to include it into our scoring function.

Training set performance

First, we assessed the predictive performance on the training set as a baseline for the further evaluations. For that we converted the 483 compounds of the training set from the SDF format into SMILES using the OpenBabel framework (O'Boyle *et al.* 2011) and predicted the bioavailability using the Pipeline Pilot prediction script. We than compared predicted binary bioavailability with the classification based on the measurements. These results showed a sensitivity of 0.96, specificity of 0.84, and Matthews correlation coefficient of 0.64 of the prediction method on its own training set. Interestingly, the method showed a particularly low positive predictive value of 0.53, indicating that many of the positively predicted compounds are false positives, while the negative predictive value is very high with 0.99, indicating very few compounds predicted as false negative in the training set. This balance chosen by the developers is particularly useful for the application of the prediction method as a filtering or prioritisation method. The overall performance, with an accuracy of 86%, leaves plenty of room for improvement, especially when considering these estimate are based on the data set the method was developed on. Not surprisingly, we found a highly significant enrichment for bioavailable compounds by ranking by bioavailability prediction score (Wilcoxon rank-sum test p-value < 10^{-15}; Kruskal-Wallis rank sum test p-value < 10^{-15}), with enrichments factors between 4 and 6.4 (compared to random) for compounds with prediction values > 2.5. The figure below shows the rate of bioavailable compounds plotted against the bioavailability prediction cut-off, illustrating the overall basic rate of bioavailable compounds of 0.15 in the training set, going up to 100% availability for compounds scoring more than 9.5.

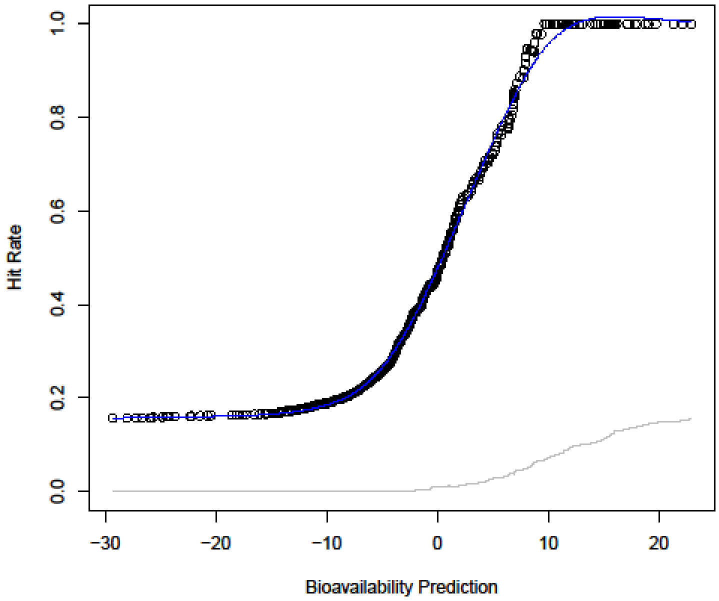

Hit rate by Predicted Bioavailability. In black circles are the empirically observed rates of bioavailable compounds for all possible cut-off of bioavailable prediction score. Dark blue is the cubic spline with 4 degrees of freedom. The empirical hit rate of the lower half of each participation is shown in grey.

External data performance *C. elegans*

For an independent evaluation, we gathered data from two independent whole animal screens of the LOPAC library of 1280 pharmaceutically active compounds (Sigma-Aldrich) in *C. elegans* (Leung *et al.* 2013; Ye *et al.* 2014). While both screens do not explicitly assess bioavailability, they observe phenotypes, which

require bioavailability of the compounds. We thus reason that these data sets can provide evaluation data for bioavailable compounds. The compounds with no effects, however, might not be influencing the phenotype, while still being bioavailable, thus do not provide evaluation data for non-bioavailable compounds.
Leung *et al.* (2013) examined the effect of the compounds on gst-4, a well know target of the SKN-1 transcription factor, using a 1536-well plate fluorescence-based screening approach. They screened the LOPAC library at 5, 10 and 20 µM concentrations each in triplicate with 1.5h exposure time and reported 27 hits which showed more than 40% inhibition in at least one replicate of one of the concentrations. 26 of these compounds could be matched to the LOPAC SD file obtained from Sigma-Aldrich, 1 compound (T-182, Tyrphostin A9) was not found in the LOPAC information from Sigma-Aldrich and thus omitted. Ye *et al.* (2014) examined the effect of the compounds in LOPAC on lifespan. They screened the library in 96-well plates at a compound concentration of 33 µM from day 1 of adulthood continuously until death. Using the provided the p-values for survival difference for each compound, we apply a stringent cut-off $p < 0.01$ to identify significant effects on lifespan, identifying 80 compounds with significant effects.

We combined the lists of compounds with observed phenotypes from the two studies to evaluate the bioavailability prediction. We converted the SD file of the LOPAC library to SMILES using OpenBabel, and predicted the bioavailability for all compounds using the Burns Script with Pipeline Pilot. We then tested whether there was enrichment in the compounds with the observed phenotype when ranked by predicted bioavailability.

We found a significant enrichment (Wilcoxon rank-sum test p-value = 0.002; Kruskal-Wallis rank sum test p-value = 0.002), with enrichment factors between 2.7 and 5.9 compared to random for compounds with prediction values > 2.5. This shows that, while the method still performs a significant enrichment on a different set of compounds, the discriminatory power is even lower. The figure below shows the hit rate plotted against the bioavailability prediction cut-off, illustrating the overall basic hit-rate of 0.08 for this data set, which goes up to 0.4 to 0.5 when only looking at the compounds with the highest predicted bioavailability.

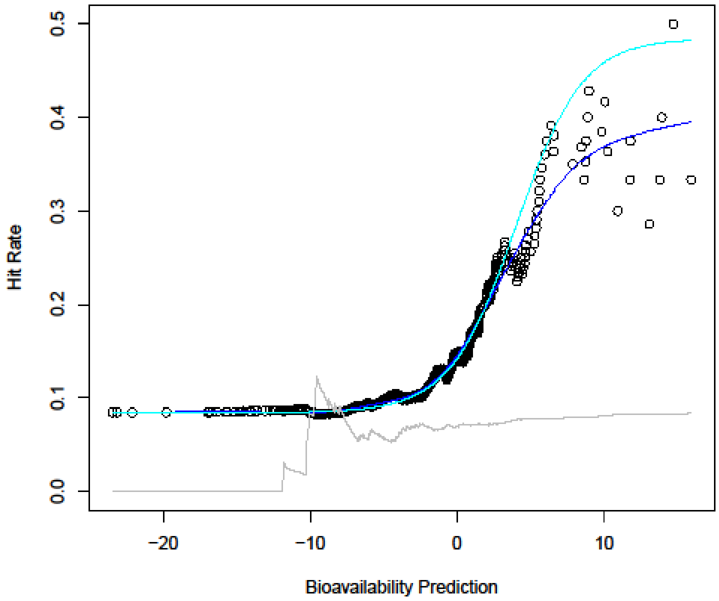

Hit rate by Predicted Bioavailability. In black circles are the empirically observed hit rates for all possible cut-off of bioavailable prediction score. Dark blue is the cubic spline with 4 degrees of freedom of the empirical hit rate, and in light blue an approximation by a logistic transformed bioavailability prediction score. The empirical hit rate of the lower half of each participation is shown in grey. Please note that there are only 20 compounds with bioavailability prediction score >7, and thus hit rate estimation is unstable.

C. elegans bioavailability ranking score

Based on these hit rate estimates, and the logistic shape of improvement, we incorporated a logistically transformed Burns bioavailability prediction score with the same location and steepness parameters as shown into our scoring function. However, we scaled the transformation to be between 0.2 and 1 instead of the observed 0.1 and 0.5, to be on the same scale as the other factors and to allow for the fact that the true bioavailability is overall likely to be significantly higher, than the estimate from those also showing one of the two considered phenotypes.

Evaluation for *D. melanogaster*

Finally, we wanted to evaluate if the bioavailability prediction method by Burns *et al.* (2010), which was developed for *C. elegans*, is transferable to *D. melanogaster* as there is no tailored prediction method available to our knowledge. For this we searched the literature for unbiased whole animal screens of compound libraries in *D. melanogaster* and found two relevant publications (Chang *et al.* 2008; Nall & Sehgal 2013).

Chang *et al.* (2008) examined the effects of 2000 compounds from the Spectrum library (MicroSource Discovery Systems Inc.) on a *D. melanogaster* model of the fragile X syndrome. They did so by screening the library at 40 µM in 96-well plates with 12-embroys per well for rescuing developmental lethality of *Frm1* mutants and found 61 hits. 42 of these hits could be matched to entries in the SD file provided by MicroSource Discovery Systems Inc. for the Spectrum library. We converted the SD file of the Spectrum library to SMILES using OpenBabel, and predicted the bioavailability for all compounds using the Burns Script with Pipeline Pilot. Three compounds (01502501, 01505308, 01506152) were excluded as their structures provided in the SD file contained incorrect valence electron configurations. We then tested whether there was enrichment of compounds with the observed phenotype when ranked by predicted *C. elegans* bioavailability. We found no significant enrichment (Willcoxon rank-sum test p-value = 0.4; Kruskal-Wallis rank sum test p-value = 0.4). While the absence of any detectable enrichment might initially seem surprising, we believe that a likely reason is the screened phenotype (rescue of embryonic lethality). This particular phenotype requires very specific compounds for an observable effect, while many other compounds might be bioavailable, but not rescue the *Frm1* mutant's specific embryonic lethality, thus not show up in the screen. Hence, the data-set might not be suitable for evaluating the predictive qualities for *D. melanogaster* bioavailability. The second publication by Nall and Sehgal (2013), screened the LOPAC library for effects on sleep in wild-type iso31 *D. melanogaster.* Nall and Sehgal (2013) screened the compounds at 20 µM for 1 week in young adult flies and found many compounds which affected sleep in at least one sex, 38 of which affected sleep in both sexes. Unfortunately, the data were not included in the publication and we were not able to obtain them from the author upon request.

We therefore, were unable to successfully validate the usefulness of the Burns predictor for predicting bioavailability in *D. melanogaster*. Since the relevance or suitability of the predictor is unclear, we chose to not use these predictions in the *D. melanogaster* ranking, but rather substitute the bioavailability score with a fixed 0.9 term. This value was chosen to allow bonuses to increase the overall score, rather than being irrelevant because of the upper limit of 1.0. If in the future a bioavailability predictor for *D. melanogaster* is published or the Burns *et al.* (2010) evaluated for *D. melanogaster*, this term can be, after transformation into the range between 0 and 1, readily used into the ranking procedure.

References

Ballester PJ, Schreyer A, Blundell TL (2014). Does a more precise chemical description of protein-ligand complexes lead to more accurate prediction of binding affinity? *J Chem Inf Model*. **54**, 944-955.

Burns AR, Wallace IM, Wildenhain J, Tyers M, Giaever G, Bader GD, Nislow C, Cutler SR, Roy PJ (2010). A predictive model for drug bioaccumulation and bioactivity in Caenorhabditis elegans. *Nat Chem Biol*. **6**, 549-557.

Chang S, Bray SM, Li Z, Zarnescu DC, He C, Jin P, Warren ST (2008). Identification of small molecules rescuing fragile X syndrome phenotypes in Drosophila. *Nat Chem Biol*. **4**, 256-263.

Leung CK, Wang Y, Malany S, Deonarine A, Nguyen K, Vasile S, Choe KP (2013). An ultra high-throughput, whole-animal screen for small molecule modulators of a specific genetic pathway in Caenorhabditis elegans. *PLoS One*. **8**, e62166-e62166.

Nall AH, Sehgal A (2013). Small-molecule screen in adult Drosophila identifies VMAT as a regulator of sleep. *J Neurosci*. **33**, 8534-8540.

O'Boyle NM, Banck M, James CA, Morley C, Vandermeersch T, Hutchison GR (2011). Open Babel: An open chemical toolbox. *J Cheminform*. **3**, 33-33.

Wang R, Fang X, Lu Y, Wang S (2004). The PDBbind database: collection of binding affinities for protein-ligand complexes with known three-dimensional structures. *Journal of medicinal chemistry*. **47**, 2977-2980.

Wang R, Lai L, Wang S (2002). Further development and validation of empirical scoring functions for structure-based binding affinity prediction. *J Comput Aided Mol Des*. **16**, 11-26.

Winn MD, Ballard CC, Cowtan KD, Dodson EJ, Emsley P, Evans PR, Keegan RM, Krissinel EB, Leslie AG, McCoy A, McNicholas SJ, Murshudov GN, Pannu NS, Potterton EA, Powell HR, Read RJ, Vagin A, Wilson KS (2011). Overview of the CCP4 suite and current developments. *Acta crystallographica. Section D, Biological crystallography*. **67**, 235-242.

Ye X, Linton JM, Schork NJ, Buck LB, Petrascheck M (2014). A pharmacological network for lifespan extension in Caenorhabditis elegans. *Aging Cell*. **13**, 206-215.
