# Supplementary material for: Drug repurposing for aging research using model organisms
Source: Aging Cell. 2017 Jun 16;16(5):1006–15. doi: 10.1111/acel.12626 (PMC5595691; doi:10.1111/acel.12626)
Supplement: Supplementary file 7 — Data S1 Zip‐Archive of all report cards. [file RC_2AI.pdf › RC_2AI.pdf]

2AI

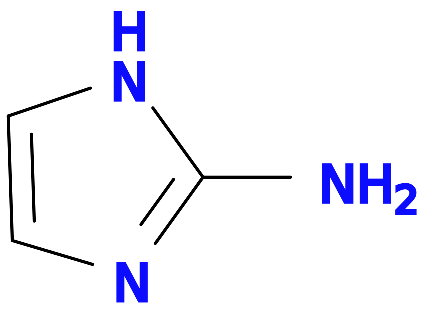

Database identifiers

ChEMBLCompound	CHEMBL291627
ZINC	ZINC17380534
eMolecules	904885

Ranking

	Rank	Score
Drosophila	513/697	0.258
C. elegans	NA	NA

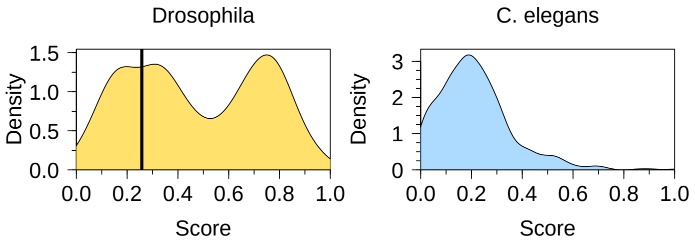

	Ageing implication	Domain conservation	Binding site conservation	Binding affinity	Bioavailability	Lipinski	Promiscuity	Purchasability	Drug approval	**Total**
Drosophila	0.624	0.802	1.0	0.351	(0.9)	0.0	-0.0	0.1	0.0	0.258
C. elegans	NA	NA	NA	NA	NA	NA	NA	NA	NA	NA

Names

No synonyms found

Roles

ChEBI entry None has no roles

Status

Approved drug (according to ChEMBL)	No
Number of Rule of 5 violations	0
Binding affinity to original target in log units (RF-Score prediction)	4.39
Burns *C. elegans* bioavailability prediction	-1.25

Compound Target Characteristics

Arginase-1

Best gene implication in ageing for this target family came from gene P07824 via mapping the annotation from RGD 2150 annotated in RGD 2014-03-11. Annotation GO 7568 (aging) was Inferred from Expression Pattern

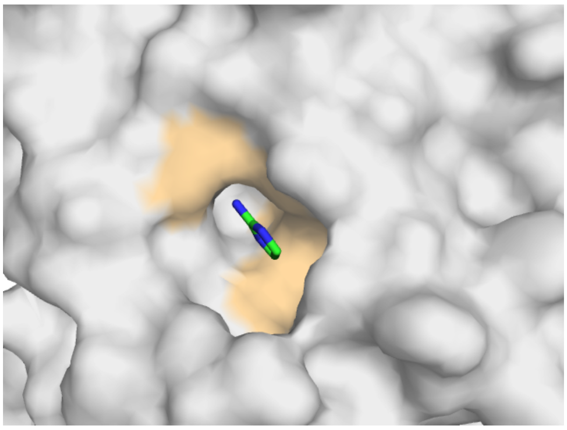

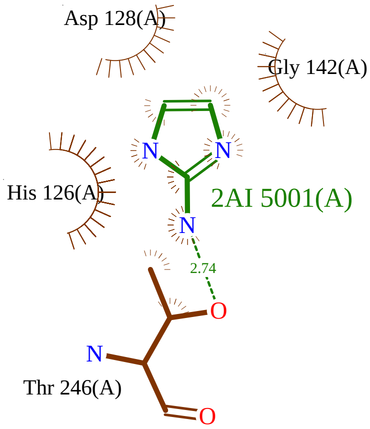

protein	amino acids contacts (binding site)
PDB:3mjl:chainA:P05089	H D G T
sp:P05089:ARGI1_HUMAN	H D G T
sp:P07824:ARGI1_RAT	H D G T
sp:Q61176:ARGI1_MOUSE	H D G T
tr:Q9NHA5:Q9NHA5_DROME	H D G T
tr:O76895:O76895_DROME	H D G T
sp:P00812:ARGI_YEAST	H D G T

	whole protein		domain-based		contact-based	
protein	ident	simil	ident	simil	ident	simil
PDB:3mjl:chainA:P05089	1.0	1.0	1.0	1.0	1.0	1.0
sp:P05089:ARGI1_HUMAN	1.0	1.0	1.0	1.0	1.0	1.0
sp:P07824:ARGI1_RAT	0.87	0.96	0.87	0.96	1.0	1.0
sp:Q61176:ARGI1_MOUSE	0.87	0.96	0.87	0.96	1.0	1.0
tr:Q9NHA5:Q9NHA5_DROME	0.35	0.71	0.36	0.74	1.0	1.0
tr:O76895:O76895_DROME	0.34	0.7	0.36	0.74	1.0	1.0
sp:P00812:ARGI_YEAST	0.39	0.71	0.4	0.71	1.0	1.0

arg (FBgn0023535) associated phenotypes

partially lethal - majority die, some die during pupal stage

(Information from FlyBase)

arg (UniProt:Q9NHA5) annotation

Cofactor: Mn(2+) Evidence=(RuleBase:RU361159); Note=Binds 2 manganese ions per subunit. (RuleBase:RU361159);
Pathway: Nitrogen metabolism; urea cycle; L-ornithine and urea from L-arginine: step 1/1. (RuleBase:RU361159).

(Information from UniProt)

arg (UniProt:O76895) annotation

Cofactor: Mn(2+) Evidence=(RuleBase:RU361159); Note=Binds 2 manganese ions per subunit. (RuleBase:RU361159);
Pathway: Nitrogen metabolism; urea cycle; L-ornithine and urea from L-arginine: step 1/1. (RuleBase:RU361159).

(Information from UniProt)
